# Unveiling the methionine cycle: a key metabolic signature and *NR4A2* as a methionine-responsive oncogene in esophageal squamous cell carcinoma

**DOI:** 10.1038/s41418-024-01285-7

**Published:** 2024-04-03

**Authors:** Xing Jin, Lei Liu, Dan Liu, Jia Wu, Congcong Wang, Siliang Wang, Fengying Wang, Guanzhen Yu, Xiaoxia Jin, Yu-Wen Xue, Dan Jiang, Yan Ni, Xi Yang, Ming-Song Wang, Zhi-Wei Wang, Yuriy L. Orlov, Wei Jia, Gerry Melino, Ji-Bin Liu, Wen-Lian Chen

**Affiliations:** 1 Cancer Institute, Longhua Hospital, Shanghai University of Traditional Chinese Medicine, Shanghai, 200032 China; 2 Shanghai Frontiers Science Center of Disease and Syndrome Biology of Inflammatory Cancer Transformation, Shanghai, 200032 China; 3 Department of Thoracic Surgery, The Affiliated Tumor Hospital of Nantong University, Nantong, 226300 China; 4 School of Medicine, Southeast University, Nanjing, 210009 China; 5 Department of Oncology, Longhua Hospital, Shanghai University of Traditional Chinese Medicine, Shanghai, 200032 China; 6 Laboratory of Digital Health and Artificial Intelligence, Zhejiang Digital Content Research Institute, Shaoxing, 312000 China; 7 Department of Pathology, The Affiliated Tumor Hospital of Nantong University, Nantong, 226300 China; 8 Pathology department, Longhua Hospital, Shanghai University of Traditional Chinese Medicine, Shanghai, 200032 China; 9 The Children’s Hospital, Zhejiang University School of Medicine, National Clinical Research Center for Child Health, Hangzhou, 310029 China; 10 Department of Oncology, Shanxi Provincial Hospital of Traditional Chinese Medicine, Shanxi, 030001 China; 11 Department of Thoracic Surgery, Shanghai Ninth People’s Hospital, Shanghai Jiao Tong University School of Medicine, Shanghai, 200011 China; 12 Department of Breast, The International Peace Maternity and Child Health Hospital, School of Medicine, Shanghai Jiao Tong University, Shanghai, 200030 China; 13 The Digital Health Institute, I.M. Sechenov First Moscow State Medical University of the Ministry of Health of the Russian Federation (Sechenov University), Moscow, 119991 Russia; 14 Institute of Cytology and Genetics, Siberian Branch of the Russian Academy of Sciences, 630090 Novosibirsk, Russia; 15 Life Sciences Department, Novosibirsk State University, Novosibirsk, 630090 Russia; 16 Institute of Life Sciences and Biomedicine, Far Eastern Federal University, Vladivostok, 690922 Russia; 17 Agrarian and Technological Institute, Peoples’ Friendship University of Russia, Moscow, 117198 Russia; 18 Department of Pharmacology and Pharmacy, Faculty of Medicine, University of Hong Kong, Hong Kong, China; 19 Department of Experimental Medicine, University of Rome “Tor Vergata”, 00133 Rome, Italy; 20 Cancer Institute, The Affiliated Tumor Hospital of Nantong University, Nantong, 226361 China

**Keywords:** Cancer metabolism, Oncogenes

## Abstract

Esophageal squamous cell carcinoma (ESCC) is a deadly malignancy with notable metabolic reprogramming, yet the pivotal metabolic feature driving ESCC progression remains elusive. Here, we show that methionine cycle exhibits robust activation in ESCC and is reversely associated with patient survival. ESCC cells readily harness exogenous methionine to generate S-adenosyl-methionine (SAM), thus promoting cell proliferation. Mechanistically, methionine augments METTL3-mediated RNA m^6^A methylation through SAM and revises gene expression. Integrative omics analysis highlights the potent influence of methionine/SAM on NR4A2 expression in a tumor-specific manner, mediated by the IGF2BP2-dependent stabilization of methylated *NR4A2* mRNA. We demonstrate that NR4A2 facilitates ESCC growth and negatively impacts patient survival. We further identify celecoxib as an effective inhibitor of NR4A2, offering promise as a new anti-ESCC agent. In summary, our findings underscore the active methionine cycle as a critical metabolic characteristic in ESCC, and pinpoint *NR4A2* as a novel methionine-responsive oncogene, thereby presenting a compelling target potentially superior to methionine restriction.

## Introduction

Esophageal squamous cell carcinoma (ESCC), a major histological type of esophageal cancer, exhibits an inferior prognosis as characterized by a median overall survival (OS) ranging from 7 to 14 months and a 5-year OS rate below 20% [1]. To better treat this disorder, it is imperative to comprehensively unveil its underlying pathobiological characteristics. As we and others demonstrate that metabolic reprogramming has emerged as a critical factor in tumorigenesis and tumor progression, producing new and valuable insights into cancer pathobiology [2–6]. Previous investigations have revealed the significant upregulation of various metabolic pathways in ESCC, such as proline/fatty acid/polyamine biosynthesis and glutamine/histidine/uridine metabolism [7–9]. Nevertheless, the key metabolic signature of ESCC in clinical tissue samples remains elusive. In our recent studies utilizing tissue and serum samples from patients with ESCC, we conducted comprehensive metabolomic analyses to dissect the metabolic features of this disease [10, 11]. However, in these studies, we focus on the perturbations but not the activity of enriched metabolic pathways in ESCC. In the current study, we re-analyzed the tissue metabolic data derived from our previous study [10] from the perspective of pathway activity and discovered a significant activation of the methionine cycle in clinical ESCC tissues. Nevertheless, a nutritional epidemiology study shows that higher intake of methionine is not associated with ESCC risk [12]. This finding confounds the role of the methionine in ESCC.

Clinical observations using positron emission tomography imaging have provided compelling evidence of an overt increase in intratumoral methionine uptake in patients with brain tumor, glioma, or multiple myeloma [13–15], indicating a pronounced reliance on this essential amino acid by human tumors. Methionine metabolism primarily involves in three key metabolic pathways: the methionine cycle, transsulfuration pathway, and methionine salvage pathway [16–18]. By regulating multiple cellular processes, methionine metabolism exerts control over cancer traits, including cancer growth, stemness, and therapeutic sensitivity [16–19]. For instance, methionine metabolism is tightly coupled to one-carbon metabolic network through the remethylation of homocysteine, thereby amplifying nucleotide synthesis [20]. Furthermore, methionine plays a crucial role in maintaining cellular redox homeostasis by providing homocysteine to fuel the transsulfuration pathway and generate the essential antioxidant glutathione [16, 18]. Additionally, methionine cycle is involved in chromatin and nucleic acid methylation through the supply of S-adenosyl-methionine (SAM), a universal methyl donor [21, 22]. The addiction of cancer cells to methionine has prompted investigations of methionine-restricted diets in combination with chemotherapy in both preclinical models and clinical trials, demonstrating promising anti-cancer efficacy [23–25]. However, concerns regarding the appropriate level and duration of methionine restriction (MR) have arisen, given the amino acid’s importance in maintaining muscle mass, neuronal functions, and redox balance [16, 18, 24, 26]. To maximize the anti-cancer potential of MR while minimizing latent adverse effect, it is imperative to identify key downstream players in methionine metabolism that contribute to tumor progression.

It is worth noting that the methionine cycle is intimately linked to intracellular RNA methylation by providing SAM [27, 28]. Among the various modifications of messenger RNA (mRNA), *N*^6^-methyladenosine (m^6^A) is the most prevalent and it significantly impacts multiple mRNA processes, including alternative splicing, nuclear exportation, decay, stabilization, and translation [29–31]. The regulation of intracellular mRNA m^6^A methylation is orchestrated by a complex machinery consisting of writer, reader, and eraser proteins [29, 30, 32]. The core subunits of the writer complex comprise methyltransferase-like 3 (METTL3), methyltransferase-like 14 (METTL14), methyltransferase-like 16 (METTL16), while well-known eraser proteins are fat mass and obesity-associated protein (FTO) and alkB homolog 5 (ALKBH5). Reader proteins involved in this process include YT521-B homology domain-containing family proteins (YTHDFs) and insulin-like growth factor 2 mRNA-binding proteins (IGF2BPs) [30–33]. Increasing lines of evidence highlight the importance of mRNA m^6^A methylation in cancer initiation and progression [28, 34, 35]. For instance, METTL3-mediated mRNA m^6^A modification, followed by YTHDF2-dependent degradation of the m^6^A-methylated mRNA of suppressor of cytokine signaling 2, a tumor suppressor, promotes liver cancer growth and migration [33].

Based on our preliminary findings demonstrating the ectopic activation of the methionine cycle in ESCC, we have formulated a hypothesis suggesting that this metabolic pathway can generate an abundant supply of SAM. Consequently, we propose that this elevated SAM level can perturb the RNA m^6^A pattern and induce changes in the expression of specific genes important for ESCC development. This study aims to thoroughly investigate and validate this hypothesis, therefore elucidating the importance of methionine addiction for ESCC and identifying methionine-responsive genes that could serve as new therapeutic targets for ESCC treatment.

## Results

### Methionine cycle is hyperactive in clinical ESCC tissues and predicts unfavorable patient survival

**Fig. 1:**
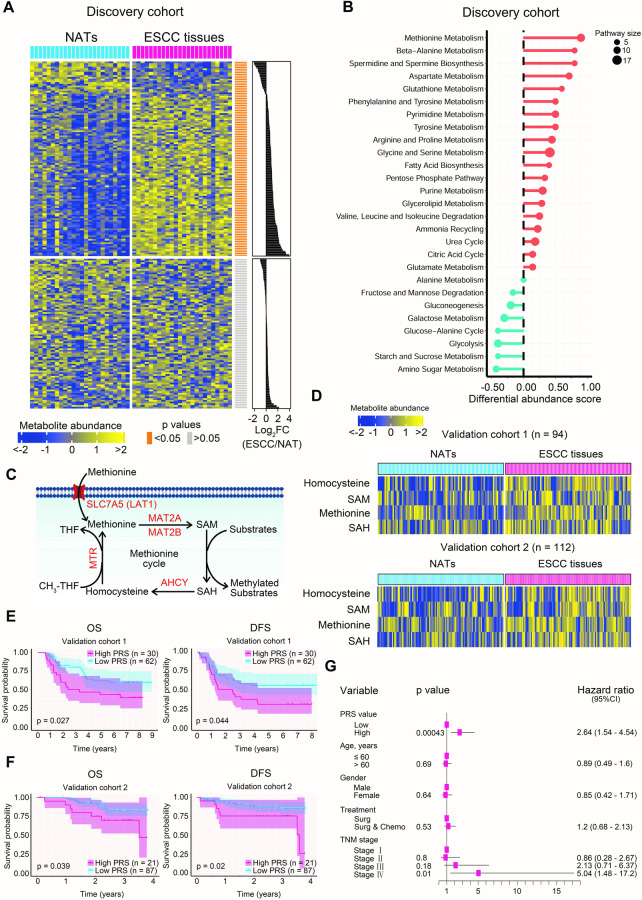
Provoked methionine cycle in clinical ESCC tissues. **A** Heatmap showing all measured metabolites in clinical ESCC tissues and paired NATs from the discovery cohort. The upper and lower panels represent differential (56.50%, 113/200) and non-differential (43.50%, 87/200) metabolites in tumor tissues, respectively. **B** Metabolic pathway enrichment analysis of clinical ESCC tissues from the discovery cohort. The differential abundance score represents the average change of measured metabolites in a pathway in ESCC tissues relative to paired NATs. Upregulation and downregulation of all measured metabolites in a pathway are indicated by red and cyan bars, respectively. Pathway size is defined by the quantity of measured metabolites participating in a particular metabolic pathway. **C** Schematic of the methionine cycle, highlighting the amino acid transporter and metabolic enzymes involved in this pathway (shown in red). **D** Differential metabolites of the methionine cycle between clinical ESCC tissues and paired NATs in validation cohorts 1 and 2. OS and DFS curves of patients with high and low PRS in validation cohort 1 (**E**) and validation cohort 2 (**F**). The cut-off value for PRS is determined in validation cohort 1 using the 70^th^ percentile. The 95% confidence limits of each survival curve are represented by shadows. **G** Forest plot displaying OS hazard ratios of PRS and several well-known clinical parameters derived from a multivariate Cox regression model fitted using the combined validation cohort 1 and validation cohort 2.

In our previous study for the metabolic features in clinical ESCC tissues [10], we concentrate on the perturbations of enriched metabolic pathways using the algorithm of metabolite set enrichment analysis [36, 37]. To assess the changes in the activity of metabolic pathways in clinical ESCC tissues, we used the reported ESCC cohort as a discovery cohort (*n* = 24, Supplementary Table S1) [10] and conducted a reanalysis of tissue metabolomic dataset from this cohort. Our analysis revealed significant alterations in 56.50% of measured metabolites, with 83.19% of these differential metabolites upregulated in ESCC tissues compared to paired normal adjacent tissues (NATs) (Fig. 1A). Using Kyoto Encyclopedia of Genes and Genomes (KEGG) pathway-based differential abundance analysis, we identified 27 enriched metabolic pathways, with nine of these pathways exhibiting upregulation (≥0.5 differential abundance score) in the tumor tissues (Fig. 1B). Notably, methionine metabolism was ranked first among these nine pathways (Fig. 1B), with elevated levels for nine out of 10 measured metabolites in this pathway (Supplementary Fig. S1A), indicating the aberrant activation of methionine metabolism in ESCC tissues. As previously described [18], the methionine cycle, a pathway in methionine metabolism, generates SAM to provide methyl groups for the methylation of diverse biological macromolecules (Fig. 1C). Our findings suggested the potential stimulation of this metabolic pathway in clinical ESCC tissues, as evidenced by increased levels of two out of four metabolites involved in the pathway, namely methionine and homocysteine (Hcy) (Supplementary Fig. S1A).

Next, we enrolled two independent validation cohorts (*n* = 94 and *n* = 112, respectively; Supplementary Tables S2 and S3) to corroborate the activation of methionine cycle in clinical ESCC tissues. Unsupervised principal component analysis (PCA) of the tissue metabolomic data from these cohorts demonstrated low dispersion of quality control samples, indicating minimal systemic measurement variability (Supplementary Fig. S1B, C). Consistent with the findings in the discovery cohort, the levels of four metabolites involved in the methionine cycle (methionine, SAM, S-adenosyl-homocysteine (SAH), and Hcy) were markedly upregulated in clinical ESCC tissues compared to paired NATs in both validation cohorts (Fig. 1D), providing further confirmation of the overactivation of this pathway in ESCC. Subsequently, we investigated whether the activity of the methionine cycle in ESCC tissues correlated with patient survival. Using four intratumoral metabolites involved in the methionine cycle (illustrated in Fig. 1D), we developed a predictive PCA model for validation cohort 1 as described in the Materials and Methods section. This model was utilized to generate prognosis-risk score (PRS), determined by the first component value, for each patient in validation cohort 1, and to predict the PRS for each case in validation cohort 2. High PRS values predicted inferior overall survival (OS) and disease-free survival (DFS) in patients with ESCC in both validation cohorts (Fig. 1E, F). Multivariate Cox regression analysis demonstrated that high PRS values predicted poor OS and DFS in the combined validation cohort 1 and validation cohort 2, independent of well-established prognostic factors such as age, gender, treatment protocol, and TNM stage (Fig. 1G and Supplementary Fig. S1D). Notably, high PRS values were correlated with increased levels of methionine cycle metabolites (Supplementary Fig. S1E,F). These results highlighted that the significance of the upregulated methionine cycle as a new and independent predictive biomarker for unfavorable survival in patients with ESCC.

To further verify the aberrant activation of the methionine cycle in ESCC, we examined the expression patterns of the transporter and metabolic enzymes involved in this pathway, as depicted in Fig. 1C. First, we reanalyzed the tissue proteomic dataset from the previously reported discovery cohort [10] and observed a significant increase in the intratumoral levels of SLC7A5, a transporter responsible for methionine uptake, as well as three key metabolic enzymes involved in the methionine cycle: methionine adenosyltransferase 2A (MAT2A), methionine adenosyltransferase 2B (MAT2B), and adenosylhomocysteinase (AHCY) (Supplementary Fig. S2A). Second, we obtained a public gene expression dataset, GSE23400 [38], which consisted of 53 pairs of clinical ESCC tissues and matched NATs, to serve as an external validation dataset. Analysis of this dataset revealed upregulation of SLC7A5 and the three metabolic enzymes mentioned above at the transcriptional level in ESCC tissues (Supplementary Fig. S2B). To further verify our findings, we performed validation assay in the aforementioned two validation cohorts. As some tissue samples in validation cohort 1 and validation cohort 2 were depleted, we enrolled a new validation cohort 3 (*n* = 41, Supplementary Table S4). Immunohistochemistry (IHC) staining of tissue sections demonstrated an overt elevation in the protein levels of SLC7A5, MAT2A, MAT2B, and AHCY in clinical ESCC tissues as relative to NATs in all three validation cohorts (Supplementary Fig. S2C–H). Collectively, our results provided comprehensive evidence of the ectopic activation of the methionine cycle at both the metabolite and metabolic enzyme levels. Furthermore, we established a reverse association between this metabolic pathway and the prognosis of patients with ESCC.

### High methionine promotes ESCC growth via SAM but low methionine causes adverse effect

**Fig. 2:**
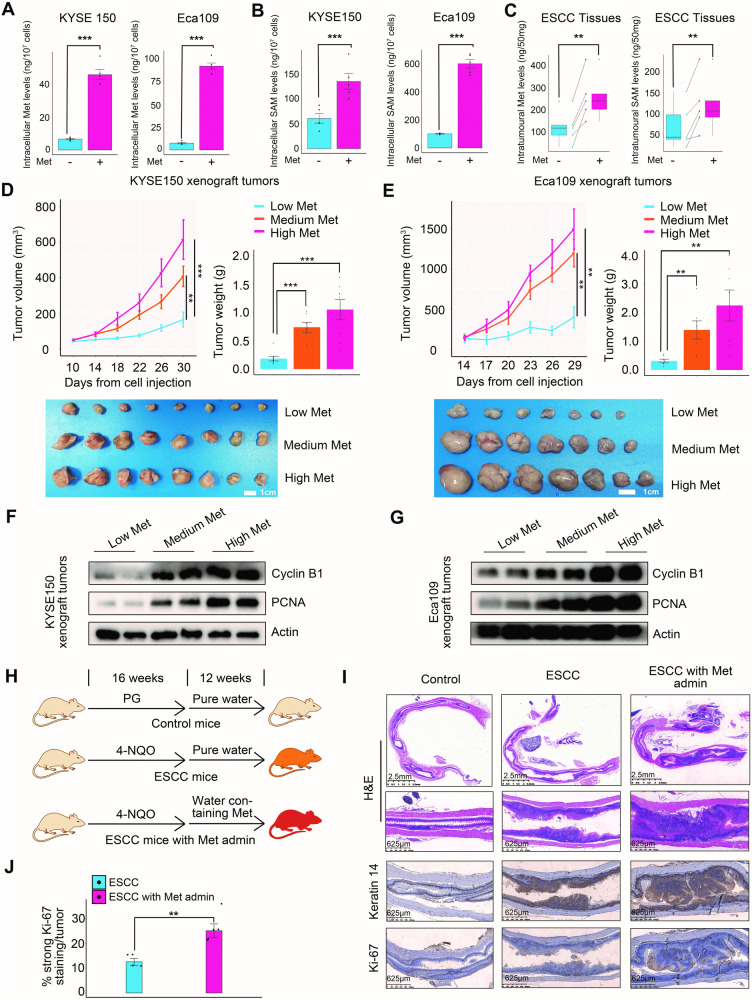
Requirement of methionine and its downstream metabolite SAM for ESCC growth. Comparison of intracellular methionine (**A**) and its downstream metabolite SAM (**B**) levels between ESCC cells cultured in medium with 10 μM methionine and methionine-free medium for 24 h. **C** Comparison of intratumoral methionine and SAM levels between primary fresh ESCC tissues cultured in medium with 10 μM methionine and methionine-free medium for 24 h. Five ESCC tissues from five patients were collected and cultured accordingly. Each ESCC tissue was halved and the acquired segments were cultured in methionine-containing and methionine-free media, respectively. Influence of dietary methionine on the growth of heterotopic ESCC tumors (**D** and **E**) and intratumoral expression of cyclin B1 and PCNA (**F** and **G**) in the subcutaneous xenograft mouse model. **H** Establishment of 4-NQO-induced orthotopic ESCC mouse model: Following a 16-week induction period, ESCC mice were randomly divided into two groups and administered pure water or water containing 10 mg/mL methionine. Propylene glycol (PG) solution, dissolved in water at a ratio of 1:60 (vol:vol), was used as vehicle. **I** Representative hematoxylin and eosin (H&E) and IHC staining of the esophagi from three groups of mice derived from the model described in **H**. Keratin 14 is used as a marker of esophageal epithelial squamous cells. **J** Quantification of the percentage of neoplastic cells with strong Ki-67 staining in esophageal tumors derived from 4-NQO-induced ESCC mice with or without methionine administration. Error bars represent mean ± standard error of the mean (SEM). ^*^p < 0.05, ^**^p < 0.01, ^***^p < 0.001 (Student’s *t* test).

As methionine is a well-known essential amino acid for human body, an important question arises regarding whether the active methionine cycle observed in clinical ESCC tissues signifies an avid uptake and utilization of exogenous methionine by ESCC cells. To address this question, we initially selected two representative ESCC cell lines, KYSE150 and Eca109, for in vitro assay. When compared to the cells cultured in a methionine-free medium, those cultured in a methionine-containing medium revealed increased levels of intracellular methionine and its downstream metabolite, SAM (Fig. 2A, B). Subsequently, we conducted ex vivo assays using freshly harvested ESCC tissues from five patients. Similar to the in vitro findings, the ESCC tissues cultured in a complete medium exhibited significantly higher levels of intratumoral methionine and SAM compared to the matched ESCC tissues cultured in a methionine-free medium (Fig. 2C). These results demonstrated that both the ESCC cell lines and primary fresh ESCC tissues from patients effectively consumed exogenous methionine to generate SAM. Moreover, ESCC cells cultivated in media enriched with SAH or Hcy displayed markedly elevated levels of intracellular SAH or Hcy compared to their counterparts cultured in methionine, SAH, and Hcy-free medium (Supplementary Fig. S3A, B). This observation suggested that ESCC cells were capable of assimilating exogenous SAH and Hcy.

We then sought to decipher the significance of methionine and SAM in the malignance of ESCC. First, in vitro assays were conducted using four ESCC cell lines, including KYSE150, Eca109, KYSE450, and KYSE30. The results demonstrated that methionine and SAM, but not SAH and Hcy, fostered the proliferation of ESCC cells in a dose-dependent manner (Supplementary Fig. S3C–F). The impact of exogenous methionine and SAM on the growth of ESCC cells may be confounded by the presence of diverse amino acids in standard fetal bovine serum (FBS) incorporated into the culture medium. To address this potential interference, a methionine-free medium supplemented with 10% dialyzed FBS was employed for validation purposes. The results validated that both exogenous methionine and SAM indeed boosted the growth of ESCC cells in a dose-dependent manner in the dialyzed medium (Supplementary Fig. S3G,H). Furthermore, methionine and SAM promoted G2/M phase progression and upregulated the expression of a G2/M progression marker cyclin B1 and a cell proliferation marker proliferating cell nuclear antigen (PCNA) in ESCC cells (Supplementary Fig. S3I–K). Second, in vivo investigations were carried out using a subcutaneous xenograft mouse model. It was observed that dietary methionine dramatically expedited heterotopic ESCC tumor growth in a dose-dependent manner, accompanied by enhanced intratumoral expression of cyclin B1 and PCNA (Fig. 2D–G). Mouse diets with distinct levels of methionine (Supplementary Table S5) were prepared as previously depicted [39]. Third, in vivo studies were performed using a 4-nitroquinoline 1-oxide (4-NQO)-induced ESCC mouse model. Oral administration of methionine via drinking water remarkably accelerated orthotopic ESCC tumor growth and upregulated the intratumoral expression of another cell proliferation marker, Ki-67 (Fig. 2H–J). Together, these findings demonstrated that exogenous methionine and its downstream metabolite SAM were essential for ESCC growth.

In line with previous findings showing the anti-cancer potency of MR against gastric cancer, colorectal cancer, and soft-tissue sarcoma [23–25], low methionine markedly hindered ESCC cell growth in vitro and in vivo (Fig. 2D–J and Supplementary Fig. S3C). Of note, as compared to high methionine diet, low methionine diet caused a substantial 11.6–14.7% body weight loss, a typical symptom of cancer-associated cachexia [40], in mice harboring ESCC xenograft tumors after 31 days of tumor transplantation (Supplementary Fig. S3L), indicating a considerable adverse effect of MR. This raised the necessity of identifying methionine-responsive oncogenes instead of MR as new therapeutic targets against ESCC.

### RNA m^6^A methylation of ESCC is augmented through a methionine-SAM-MELLT3 cascade

**Fig. 3:**
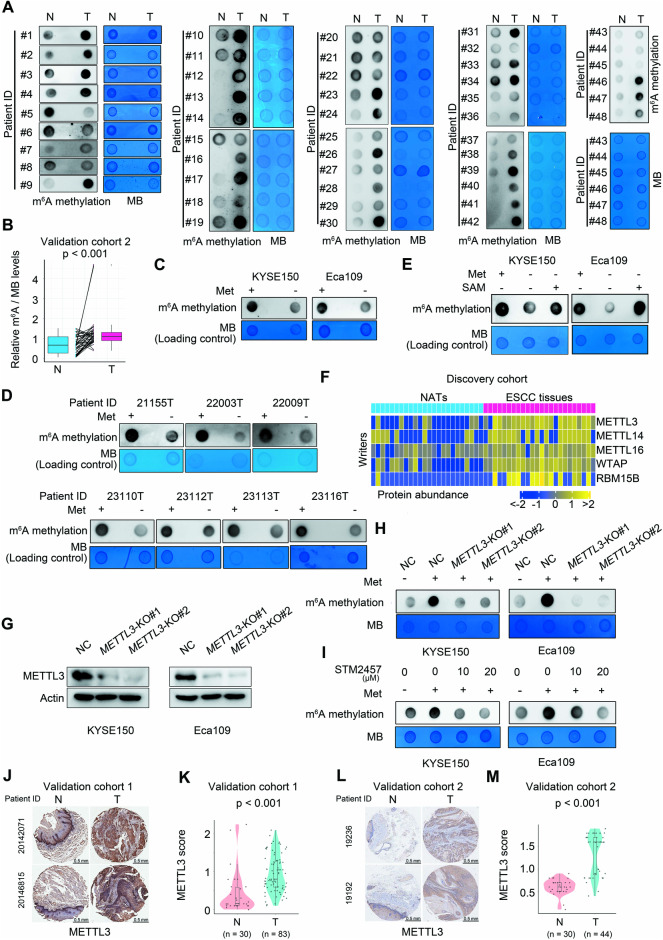
Intensified RNA m^6^A methylation in ESCC elicited by the methionine-SAM-METTL3 cascade. **A** Dot blot assay comparing RNA m^6^A content between clinical ESCC tissues and paired NATs from validation cohort 2 (*n* = 48). **B** Quantification of RNA m^6^A levels of paired tissues described in **A**, with *p* value calculated using paired Wilcoxon rank-sum test. Impact of methionine removal from culture medium on RNA m^6^A abundance in ESCC cell lines (**C**) and primary fresh ESCC tissues from patients (**D**) after 24 h of in vitro culture. **E** Recovery of intracellular RNA m^6^A content in the absence of methionine through supplementation with 100 μM SAM for 24 h. **F** Proteomic analysis of the expression of three writer enzymes and two cofactors in the RNA m^6^A machinery between clinical ESCC tissues and paired NATs from discovery cohort. **G**, **H** Influence of *METTL3* abrogation on the intracellular RNA m^6^A intensity in ESCC cells in the presence of methionine. **I** Influence of STM2457 treatment on the intracellular RNA m^6^A abundance in ESCC cells in the presence of methionine. Comparison of METTL3 expression between ESCC tissues and NATs from validation cohort 1 (**J** and **K**) and validation cohort 2 (**L** and **M**). Representative IHC images (**J** and **L**) and statistical violin-box-scatter plots (**K** and **M**) are presented, with *p* values calculated using Wilcoxon rank-sum test. NC non-target control.

Given the known role of methionine and its downstream metabolite SAM in promoting RNA m^6^A methylation [27, 28], and the importance of RNA m^6^A methylation in modulating gene expression [29, 30], we speculated that methionine would revise the expression of certain oncogenes via altering RNA m^6^A modification to foster ESCC growth. First, we sought to ascertain whether the m^6^A levels were elevated in intratumoral RNAs of clinical ESCC tissues. To address this, we extracted RNA samples from 48 ESCC tissues and their matched NATs derived from patient cohort 2 and examined the m^6^A intensity of these specimens using a dot blot assay. The result revealed a substantial increase in RNA m^6^A methylation in clinical ESCC tissues as relative to paired NATs (Fig. 3A, B). Of importance, upon removal of methionine from the culture medium, both ESCC cell lines and primary fresh ESCC tissues from patients exhibited an evident abatement of RNA m^6^A methylation (Fig. 3C, D). Notably, SAM supplementation dramatically restored this RNA modification (Fig. 3E). These findings conclusively demonstrated the essential role of methionine and its metabolite SAM in the maintenance of RNA m^6^A methylation in ESCC.

Subsequently, we aimed to uncover the underlying mechanism responsible for the installation of m^6^A in RNA in ESCC cells. Reanalysis of the proteomic data from the discovery cohort of our previously published study [10] manifested a significant upregulation of three m^6^A writer enzymes, namely METTL3, METTL14, and METTL16, along with two cofactors, WTAP and RBM15B, in clinical ESCC tissues (Fig. 3F). Among these writer enzymes, METTL3 has been reported to enhance the m^6^A modification in RNA transcripts in ESCC cells [41]. Therefore, we hypothesized that METTL3 mediated the methionine-induced RNA m^6^A methylation in ESCC cells. Indeed, the deletion of *METTL3* severely impaired the methionine-elicited RNA m^6^A modification in ESCC cells (Fig. 3G, H). Consistently, treatment with a well-established METTL3 inhibitor, STM2457 [42], also remarkably restrained the methionine-induced RNA m^6^A signal in ESCC cells (Fig. 3I). Moreover, analysis of clinical samples revealed that the expression of METTL3 was prominently increased in clinical ESCC tissues from three validation cohorts as compared to NATs (Fig. 3J–M and Supplementary Fig. S4).

Collectively, our findings demonstrated that RNA m^6^A modification was notably upregulated in clinical ESCC tissues through a methionine-SAM-METTLE3 cascade.

### Integrative omics analysis reveals a positive linkage between the expression of *NR4A2* and the m^6^A intensity in its mRNA under methionine stimulation

**Fig. 4:**
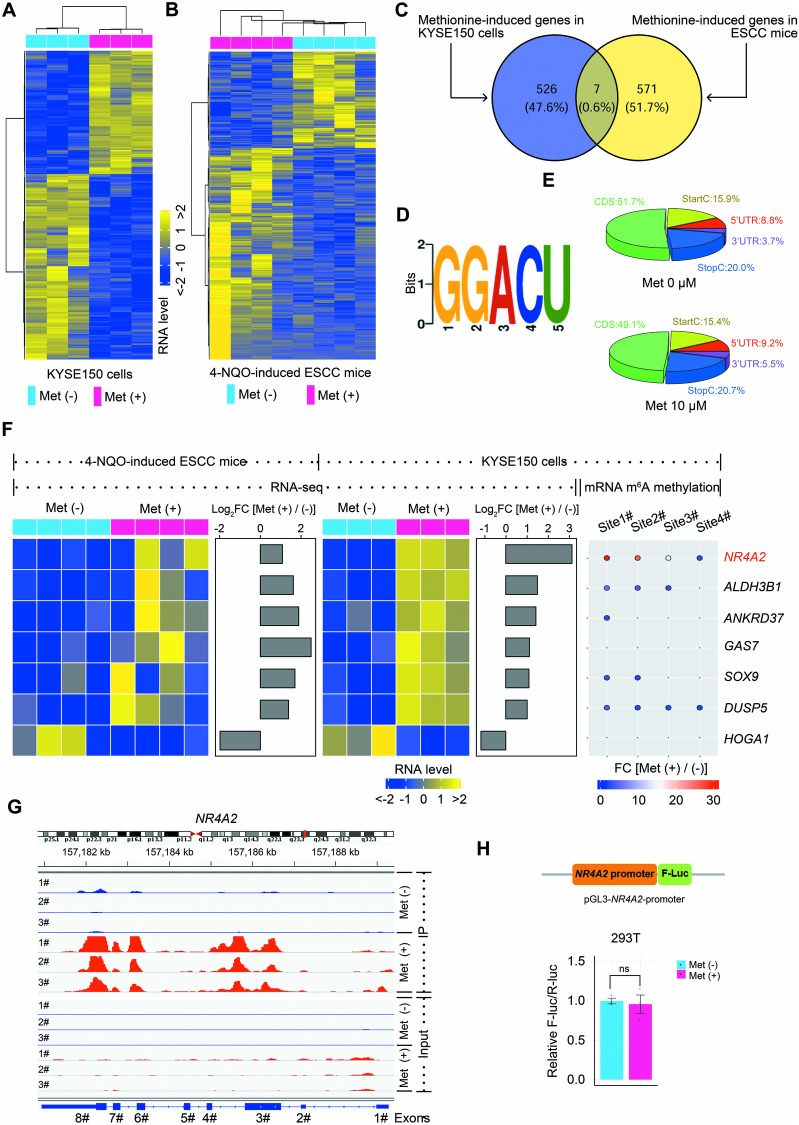
*NR4A2* as a prominent downstream gene of methionine identified by integrative RNA-seq and MeRIP-seq analysis. **A** Hierarchically clustered heatmap showing differentially expressed genes between KYSE150 cells cultured in medium with 10 μM methionine and methionine-free medium. **B** Hierarchically clustered heatmap displaying differentially expressed genes in neoplastic esophagi of 4-NQO-induced ESCC mice fed with methionine-containing water and pure water. **C** Venn diagram showing seven genes consistently modulated by methionine in both KYSE150 cells in vitro and 4-NQO-induced ESCC mice in vivo. **D** Predominant consensus motif, GGACU, identified from the top 1000 m^6^A peaks in KYSE150 cells. **E** Pie charts illustrating the distribution of m^6^A peaks in different regions across the entire set of mRNA transcripts of KYSE150 cells treated with or without methionine. Methionine-elicited m^6^A peaks are predominantly enriched in 3′-boundaries and 3′-UTRs of mRNAs. **F** Integrative analysis of methionine-elicited expression changes and m^6^A peaks for seven methionine-modulated genes in **C** in esophageal tissues of 4-NQO-induced mice in vivo and KYSE150 cells in vitro. **G** Analysis of m^6^A peaks in *NR4A2* mRNAs between KYSE150 cells cultured in medium with 10 μM methionine and methionine-free medium, identifying four strongly methionine-provoked m^6^A peaks. **H** The fusion of *NR4A2* promoter sequence with firefly luciferase reporter and the influence of methionine supplement on the relative F-luc mRNA abundance of *NR4A2* promoter reporter in HEK 293T cells. UTR untranslated regions, CDS coding sequence.

Methionine metabolism has been shown to impact RNA m^6^A methylation by providing the universal methyl donor SAM, consequently modifying gene expression [30, 43]. Based on our findings, we hypothesized that methionine would alter intracellular RNA m^6^A patterns and gene expression profiles to promote ESCC progression. To investigate this, we conducted RNA-sequencing (RNA-seq) analyses using KYSE150 cells cultured in vitro and esophageal tissues from 4-NQO-induced ESCC mice to identify methionine-regulated genes. There were 8,527 genes to be identified in both in vitro and in vivo models. Gene-wise (inter-sample) correlation analysis revealed a striking discrepancy between the in vitro and in vivo conditions regarding the impact of methionine administration on gene expression, as shown by a median gene-wise correlation value of −0.024 and a low percentage (4.2%) of genes with significant positive Spearman correlations (Supplementary Fig. S5A). Subsequently, we observed significant perturbations in the expression of 533 genes in the in vitro model and 578 genes in the in vivo model upon methionine treatment (Benjamini–Hochberg adjusted *p* < 0.05, fold change (FC) > 2 or <−2) (Fig. 4A, B). Importantly, Venn diagram revealed seven genes consistently modulated by methionine in both in vitro and in vivo settings, including nuclear receptor subfamily 4 group A member 2 (*NR4A2*), aldehyde dehydrogenase 3 family member B1 (*ALDH3B1*), ankyrin repeat domain 37 (*ANKRD37*), growth arrest specific 7 (*GAS7*), SRY-box transcription factor 9 (*SOX9*), dual specificity phosphatase 5 (*DUSP5*), and 4-hydroxy-2-oxoglutarate aldolase 1 (*HOGA1*) (Fig. 4C).

Subsequently, we employed methylated RNA immunoprecipitation sequencing (MeRIP-seq) to investigate the influence of methionine on RNA m^6^A signature in KYSE150 cells. Consistent with previous study [44], the consensus m^6^A motif of GGACU was highly enriched in the immunopurified mRNAs of KYSE150 cells (Fig. 4D). Of note, the methionine-induced m^6^A peaks were predominantly located near 3′-boundaries and in 3′-untranslated regions (UTRs) of mRNAs (Fig. 4E, Supplementary Fig. S5B, C). Further analysis revealed that methionine treatment significantly augmented m^6^A modification at 3200 sites while attenuating it at 3,029 sites within RNA transcripts (*p* < 0.005, FC > 2 or < −2) (Supplementary Table S6). Kyoto Encyclopedia of Genes and Genomes (KEGG) pathway enrichment analysis revealed that m^6^A-modified transcripts were enriched in cancer-related pathways (such as pathways in cancer, Notch signaling pathway, and pancreatic cancer pathway) and biological processes (such as cell cycle and gap junction pathways, and AGE-RAGE signaling pathway in diabetic complications) (Supplementary Fig. S5D).

We then performed an integrative analysis of the seven genes consistently regulated by methionine in vitro and in vivo, using RNA-seq and MeRIP-seq data. Among these genes, *NR4A2*, *ALDH3B1*, *ANKRD37*, *GAS7*, *SOX9*, and *DUSP5* exhibited upregulation, while *HOGA1* showed downregulation upon methionine treatment. Notably, m^6^A peaks were observed only in *NR4A2*, *ALDH3B1*, *ANKRD37*, *SOX9*, and *DUSP5* (Fig. 4F). This finding suggested that methionine may elicit gene expression changes through m^6^A modification. We noticed that, in the presence of methionine, there were four major m^6^A peaks in both *NR4A2* and *DUSP5* mRNAs, and three m^6^A peaks in the *NR4A2* mRNA had FC values > 10 (Fig. 4F and Supplementary Fig. S5E–J, and Supplementary Table S6). Therefore, we focused on NR4A2 for further investigation. Of note, four major m^6^A peaks in the *NR4A2* mRNA elicited by methionine were located in the exon 3, exon 6, exon 8, and 3′-UTR (Fig. 4G). Therefore, methionine-induced m^6^A methylation in the *NR4A2* mRNA was closely linked to the upregulation of *NR4A2* expression in the presence of methionine. Furthermore, we constructed a reporter minigene harboring the promoter sequence of *NR4A2*. The supplement of methionine did not perturb the expression of the *NR4A2* promoter (Fig. 4H), suggesting that methionine treatment increased the expression of *NR4A2* not via upregulating its transcription. Taken together, our results demonstrated that methionine administration remarkably perturbed the patterns of gene expression and RNA m^6^A methylation in ESCC cells, particularly inducing the expression and m^6^A modification of *NR4A2*.

### Methionine stimulates *NR4A2* expression by enhancing its mRNA stability through a SAM-METTL3-m^6^A-IGF2BP2 cascade

**Fig. 5:**
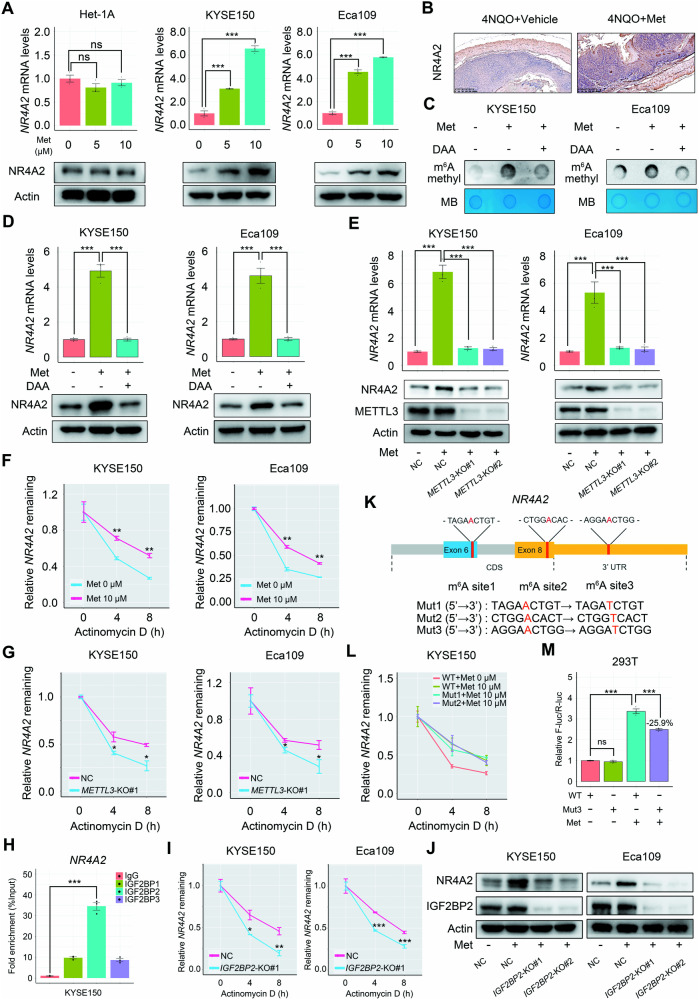
Methionine inducing *NR4A2* expression via the METTL3-m^6^A-IGF2BP2 cascade. **A**, **B** Influence of methionine on NR4A2 expression at mRNA and protein levels in ESCC cells and the non-transformed esophageal epithelial cell line Het-1A. Cells were cultured for 24 h. **B** Effect of methionine administration via drinking water on the protein abundance of NR4A2 in the neoplastic cells of esophagi in the 4-NQO-induced ESCC mouse model. Treatment with the methylation inhibitor DAA at 10 μM for 24 h affecting methionine-elicited RNA m^6^A methylation (**C**) and NR4A2 expression at mRNA and protein levels (**D**) in ESCC cells. **E** Perturbation of methionine-elicited NR4A2 expression at both mRNA and protein levels in ESCC cells by *METTL3* ablation. **F** *NR4A2* mRNA degradation curves over time in ESCC cells treated with or without methionine in the presence of the transcription inhibitor actinomycin D. **G** mRNA degradation curves of *NR4A2* over time in ESCC cells with or without *METTL3* deletion under the treatment of actinomycin D. Cells were cultured in complete medium. **H** RIP-qPCR revealing the association of *NR4A2* mRNA with IGF2BPs in KYSE150 cells. **I** mRNA degradation curves of *NR4A2* over time in ESCC cells with or without *IGF2BP2* deletion under the treatment of actinomycin D. Cells were cultured in complete medium. **J** Disturbance of NR4A2 protein levels in ESCC cells by *IGF2BP2* deletion. Cells were cultured in complete medium. **K** Schematic diagram showing three predicted m^6^A motifs within *NR4A2* mRNA with high confidence, and construction of three *NR4A2* mutants containing these motifs. Mut1 and Mut2 represent full-length coding sequence mutants of *NR4A2* with A-T substitutions (shown in red) within m^6^A consensus. Mut3 represents a 715-bp fragment of the 3′-UTR of *NR4A2* with an A-T substitution (shown in red) within m^6^A consensus. **L** mRNA degradation curves of wild-type *NR4A2* (*NR4A2*-WT), *NR4A2* Mutant 1 (*NR4A2*-Mut1), and *NR4A2* Mutant 2 (*NR4A2*-Mut2) in the presence of methionine, along with *NR4A2*-WT in the absence of methionine, under the treatment of actinomycin D. **M** Relative F-luc mRNA levels of *NR4A2*-WT or *NR4A2* Mutant 3 (*NR4A2*-Mut3) reporters in HEK 293T cells with or without methionine treatment. NC non-target control, WT wild-type, Mut mutant, F-luc firefly luciferase, R-luc renilla luciferase. Error bars represent mean ± SEM. ^*^p < 0.05, ^**^p < 0.01, ^***^p < 0.001 (Student’s *t* test).

Our analyses using RNA-seq, quantitative reverse transcription polymerase chain reaction (qRT-PCR), western blot and IHC assays revealed that methionine induced *NR4A2* expression in ESCC at both the mRNA and protein levels in both in vitro and in vivo models (Figs. 4F and 5A, B). Importantly, methionine specifically elicited *NR4A2* expression in tumor cells, as there was no change in NR4A2 expression in the presence of methionine in a non-transformed esophageal epithelial cell line, Het-1A (Fig. 5A). Furthermore, SAM also stimulated *NR4A2* expression in ESCC cells at both the mRNA and protein levels (Supplementary Fig. S6A), indicating that methionine-elicited *NR4A2* expression occurred through its downstream metabolite SAM.

To understand how methionine and SAM triggered *NR4A2* expression in ESCC cells, we hypothesized that these metabolites activated *NR4A2* expression through METTL3-mediated mRNA m^6^A methylation. Indeed, treatment of ESCC cells with a global methylation inhibitor, 3-deazaadenosine (DAA), substantially declined methionine and SAM-induced NR4A2 expression at both the mRNA and protein levels (Fig. 5C, D, Supplementary Fig. S6B, C). Moreover, deletion of *METTL3* in ESCC cells dramatically restrained methionine and SAM-elicited global RNA m^6^A methylation (Fig. 3G, H and Supplementary Fig. S6D, E) and attenuated NR4A2 expression at both the mRNA and protein levels (Fig. 5E). Additionally, treatment with a METTL3 inhibitor STM2457 also downregulated NR4A2 expression (Supplementary Fig. S6F). These findings demonstrated that methionine and SAM upregulated *NR4A2* expression at the RNA level through METTL3-mediated m^6^A methylation.

Previous study has shown that m^6^A modification can promote mRNA stability and storage, thereby increasing gene expression output [31]. In line with this, treatment with the transcription inhibitor actinomycin D revealed that methionine administration overtly retarded *NR4A2* mRNA decay (Fig. 5F). Conversely, abrogation of *METTL3* remarkably expedited the degradation of *NR4A2* mRNA induced by methionine in the presence of actinomycin D (Fig. 5G). Collectively, these findings indicated that methionine-induced *NR4A2* expression in ESCC cells was mediated by METTL3-m^6^A-triggered mRNA stabilization.

Next, we explored which reader protein was responsible for interpreting the m^6^A modification in *NR4A2* mRNA. Insulin-like growth factor 2 mRNA-binding proteins (IGF2BPs), including IGF2BP1/2/3, are well-established m^6^A readers that recognize the consensus GG(m^6^A)C sequence, primarily located near stop codons and in 3′-UTRs, thereby promoting the stability and storage of target mRNA transcripts [31]. Given one of the four m^6^A peaks in *NR4A2* mRNA induced by methionine was located in the 3′-UTR, we speculated that IGF2BPs would recognize this m^6^A peak and promote mRNA stability. To investigate this, we performed RNA immunoprecipitation-quantitative polymerase chain reaction (RIP-qPCR) analysis and found that IGF2BP2 exhibited the highest affinity for *NR4A2* mRNA in KYSE150 cells (Fig. 5H). We hypothesized that IGF2BP2 recognized the m^6^A sites in *NR4A2* mRNA and stabilized it. Indeed, deletion of *IGF2BP2* remarkably accelerated *NR4A2* mRNA decay and reduced the protein abundance of NR4A2 in ESCC cells (Fig. 5I, J). Thus, methionine could intensify the stability of *NR4A2* mRNA and increase its expression output through a SAM-MELLT3-m^6^A-IGF2BP2 cascade.

Finally, we aimed to determine which m^6^A in *NR4A2* mRNA was required for methionine-induced mRNA stabilization. Based on the MeRIP-seq data, we predicted three m^6^A consensus sequences within *NR4A2* mRNA with high confidence, located in exon 6, exon 8, and 3′-UTR, respectively (Fig. 5K). Consequently, we constructed two mutants containing the full-length coding sequence of *NR4A2* with A-T substitutions in m^6^A consensus sequences in exon 6 or exon 8 (Fig. 5K). In the presence of methionine, these two mutants did not alter *NR4A2* mRNA decay rate compared to the wild-type *NR4A2*, as shown by qRT-PCR results (Fig. 5L). Furthermore, we generated reporter minigenes containing wild-type or mutant 3′-UTR fragments of *NR4A2* (Fig. 5K and Supplementary Fig. S6G). Mutation of the m^6^A consensus sequence in the 3′-UTR led to a 25.9% decrease in methionine-induced NR4A2 expression compared to the wild-type 3′-UTR fragment of NR4A2 (Fig. 5M). Together, these results demonstrated that m^6^A modification in the 3′-UTR of *NR4A2* was required for the methionine-elicited stabilization of *NR4A2* mRNA.

### *NR4A2* facilitates ESCC growth both in vitro and in vivo

**Fig. 6:**
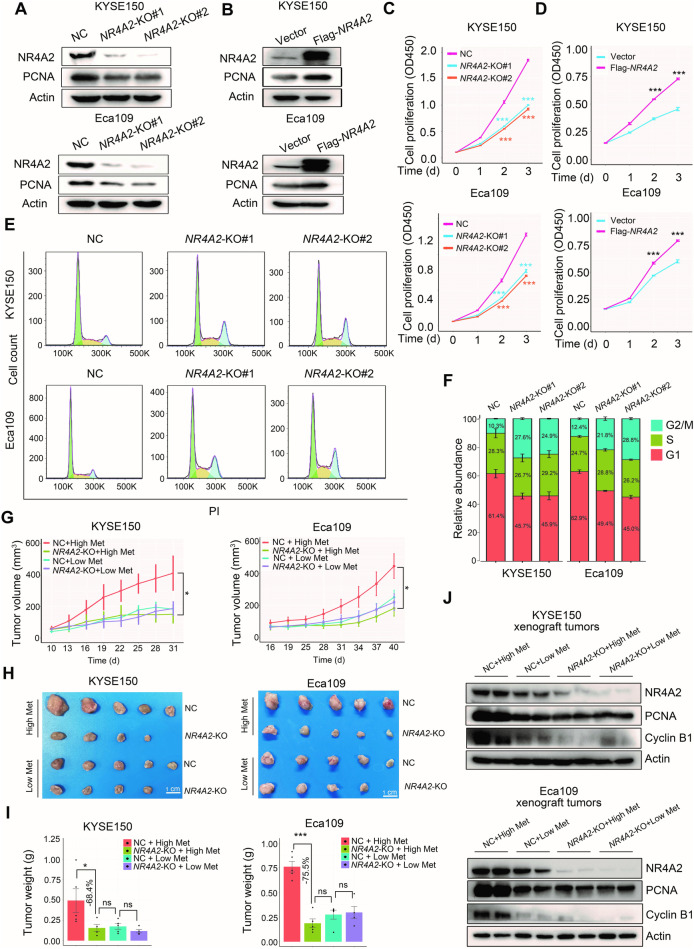
A critical role of NR4A2 in ESCC growth. **A** Western blot assay confirming *NR4A2* deletion in ESCC cells using the CRISPR-Cas9 approach and the influence of *NR4A2* ablation on PCNA expression. **B** Western blot assay confirming NR4A2 overexpression of in ESCC cells and the effect of enforced NR4A2 expression on PCNA levels. Cell proliferation curves over time showing the impact of *NR4A2* deletion (**C**) and NR4A2 overexpression (**D**) on ESCC cell propagation in complete medium. *P* values at each time point were calculated by comparing with the NC cells or cells transfected with blank vector. **E**, **F** Flow cytometric analysis revealing G2/M phase arrest of ESCC cells with *NR4A2* deletion. Cells were cultured in complete medium. Three biological replicates were performed. **G**–**I** Influence of *NR4A2* abrogation on ESCC xenograft tumor growth under conditions of high and low dietary methionine. Tumor growth curves, tumor image, and statistical analysis of tumor weight are presented in **G**, **H**, and **I**, respectively. **J** Western blot analysis revealing the effect of *NR4A2* knockout on intratumoral expression of PCNA and cyclin B1 under conditions of high and low dietary methionine. NC non-target control. Error bars represent mean ± SEM. ^*^p < 0.05, ^***^p < 0.001 (Student’s *t* test).

After identifying *NR4A2* as a methionine-responsive gene, we studied its biological roles. We observed that *NR4A2* deletion hindered ESCC cell proliferation, while enforced NR4A2 expression augmented ESCC cell multiplication (Fig. 6A–D). Furthermore, *NR4A2* ablation led to cell cycle arrest at G2/M phase (Fig. 6E, F), indicating that *NR4A2* was vital for ESCC cell propagation in vitro.

We utilized a xenograft tumor mouse model to verify the oncogenic role of *NR4A2* in ESCC. High dietary methionine dramatically expedited the tumor growth of implanted control ESCC cells but not ESCC cells lacking *NR4A2* (Fig. 6G–I). Consistently, in the presence of high dietary methionine, intratumoral expression of PCNA and Cyclin B1 was remarkably repressed by *NR4A2* deletion (Fig. 6J). Additionally, under low dietary methionine conditions, *NR4A2* abrogation did not further impede ESCC tumor growth and decline intratumoral expression of PCNA and Cyclin B1 (Fig. 6I–J). These results demonstrated that *NR4A2* expression was crucial for methionine-induced ESCC tumor amplification in vivo. Notably, *NR4A2* abrogation generated an anti-ECCC effect in the presence of high dietary methionine, comparable to the effect of a low methionine diet (Fig. 6I), indicating that NR4A2 inhibition could serve as a promising alternative to MR.

### *NR4A2* is highly expressed in ESCC and reversely linked to patient prognosis

**Fig. 7:**
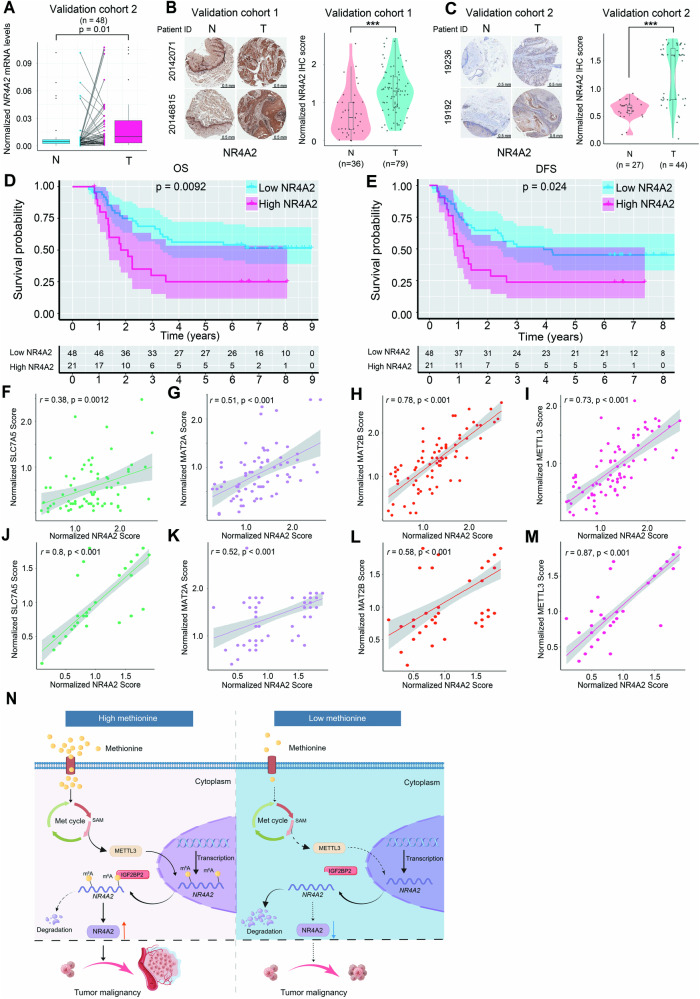
The clinical relevance of NR4A2 in patients with ESCC. **A** qRT-PCR examination comparing *NR4A2* mRNA levels between clinical ESCC tissues and paired NATs derived from validation cohort 2 (*n* = 48). *P* values were computed using the paired Wilcoxon rank-sum test. IHC staining analysis comparing NR4A2 protein levels between clinical ESCC tissues and NATs derived from validation cohort 1 (**B**) and validation cohort 2 (**C**). Representative IHC images and statistical violin-box-scatter plots are shown. *P* values were calculated using the Wilcoxon rank-sum test. OS (**D**) and DFS (**E**) curves of patients with high and low NR4A2 in validation cohort 1. The 70^th^ percentile of NR4A2 IHC scores is used as the cut-off value. The 95% confidence limits of each survival curve are displayed by shadows. **F**–**M** Correlation analysis between NR4A2 and SLC7A5, MAT2A, MAT2B, or METTL3 in clinical ESCC tissues derived from validation cohort 1 (**F**–**I**) or combined validation cohort 2 and validation cohort 3 (**J**–**M**). Correlation coefficient (r) values and statistical *p* values were acquired by Spearman correlation analysis. Confidential intervals of regression lines fitted by a linear model are shown by shadows. **N** A new tumor-promoting mechanism of methionine in ESCC unveiled by this study. When compared to low methionine, high methionine stimulated methionine cycle and accelerated intracellular SAM production to intensify m^6^A methylation in *NR4A2* mRNA in a METTL3-dependent manner. Next, a reader protein IGF2BP2 recognized a specific m^6^A site in 3′-UTR of *NR4A2* mRNA, promoting its stability and storage. Consequently, this resulted in an increase in NR4A2 expression, therefore boosting ESCC growth.

Finally, we examined the expression of *NR4A2* in clinical ESCC tissues and assessed its clinical significance. First, qRT-PCR analysis of tissue samples from validation cohort 2 revealed significantly elevated *NR4A2* mRNA levels in ESCC tissues compared to paired NATs (Fig. 7A). Second, IHC analysis of tissue samples from validation cohorts 1, 2, and 3 showed a substantial increase in NR4A2 protein abundance in ESCC tissues as relative to NATs (Fig. 7B, C, Supplementary Fig. S7A, B). Third, western blot analysis of tissue samples from validation cohort 2 demonstrated a marked upregulation of NR4A2 protein content in ESCC tissues compared to paired NATs (Supplementary Fig. S7C, D). Collectively, these results indicated that NR4A2 expression was elevated in clinical ESCC tissues at both the mRNA and protein levels.

Prognostic analysis using IHC data from validation cohort 1 revealed that high NR4A2 expression in ESCC tissues predicted unfavorable OS and DFS of patients (Fig. 7D, E). As depicted above, increased activity of methionine cycle predicted inferior prognosis in patients with ESCC (Fig. 1E, F). These findings suggested the potential involvement of the methionine cycle-NR4A2 axis in the disease progression of ESCC patients.

Furthermore, the IHC staining data of clinical ESCC tissues from validation cohorts 1, 2, and 3 showed an intimate and positive correlation between the IHC scores of NR4A2 and those of SLC7A5, MAT2A, MAT2B, and METTL3, respectively (Fig. 7F–M). This underscored the existence of a methionine cycle-METTL3-mRNA m^6^A-NR4A2 cascade in ESCC.

In summary, the current study presented a new tumor-promoting mechanism of methionine in ESCC (Fig. 7N). When compared to low methionine, high methionine provoked methionine cycle and expedited intracellular SAM generation in ESCC cells. SAM supplied active methyl group and enhanced m^6^A methylation in *NR4A2* mRNA, primarily in a METTL3-dependent manner. Subsequently, a reader protein IGF2BP2 bound to a specific m^6^A site in 3′-UTR of *NR4A2* mRNA, promoting its stability and storage. Ultimately, this led to a significant increase in gene expression output of NR4A2, thereby facilitating ESCC growth.

### Celecoxib is a NR4A2 inhibitor with evident anti-ESCC efficacy

**Fig. 8:**
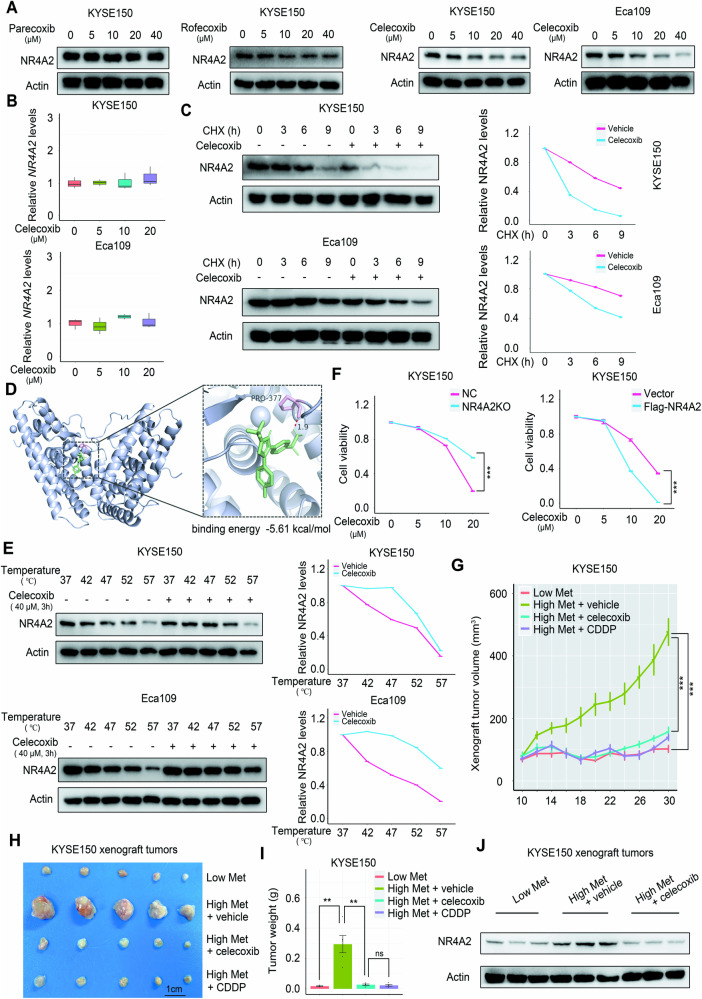
Celecoxib as a NR4A2 inhibitor with anti-ESCC potency. **A** Influence of well-known COX-2 inhibitors at distinct concentrations on intracellular NR4A2 protein abundance of ESCC cells. **B** Effect of celecoxib at different concentrations on intracellular *NR4A2* mRNA levels of ESCC cells. **C** Influence of celecoxib at 40 μM on intracellular NR4A2 protein abundance of ESCC cells over time under the condition of CHX treatment. The curves on the right side of the western blot images indicate the quantification of protein levels. **D** Celecoxib (green) interacting with NR4A2 motifs in the docking model. The hydrogen bond between this compound and NR4A2 was highlighted by red dashed line. **E** Western blot assays showing intracellular NR4A2 protein abundance of ESCC cells treated with or without celecoxib in the CETSA experiments. The curves on the right side of the western blot images indicate the quantification of protein levels. **F** Impact of *NR4A2* deletion or NR4A2 overexpression on the sensitivity of KYSE150 cells to celecoxib treatment. **G**–**I** Influence of celecoxib and CDDP on KYSE150 xenograft tumor growth under high dietary methionine condition. Tumor growth curves, tumor image, and statistical analysis of tumor weight are presented in **G**, **H**, and **I**, respectively. **J** Western blot analysis displaying the influence of celecoxib administration on intratumoral NR4A2 expression under high dietary methionine condition. Error bars represent mean ± SEM. ^*^p < 0.05, ^***^p < 0.001 (Student’s *t* test).

Previous studies find that the transcription of NR4A2 can be inhibited by cyclooxygenase-2 (COX-2) inhibitors, including parecoxib and celecoxib [45, 46]. Thus, we performed a screen using parecoxib, rofecoxib, and celecoxib, and found that only celecoxib obviously downregulated the protein abundance of NR4A2 in ESCC cells in a dose-dependent manner (Fig. 8A). However, celecoxib treatment did not perturb *NR4A2* transcription in ESCC cells (Fig. 8B). Of note, in the presence of cycloheximide (CHX), an agent blocking the elongation phase of eukaryotic protein translation, supplement of celecoxib dramatically accelerated the degradation of NR4A2 (Fig. 8C). These findings suggested that celecoxib could enhance the protein stability of NR4A2. Subsequently, we ascertained whether this compound could bind to NR4A2 to exert its function. First, molecular docking analysis revealed that celecoxib could bind to NR4A2 with a binding energy value of −5.61 kcal/mol (Fig. 8D). Second, cellular thermal shift assay (CETSA) demonstrated that celecoxib supplement caused a remarkable thermal shift for NR4A2 protein (Fig. 8E), further indicating a direct interaction between this compound and NR4A2 protein.

Subsequently, we investigated whether celecoxib exerted anti-ESCC efficacy in a NR4A2-dependent manner. First, in vitro surveys showed that *NR4A2* deletion declined the sensitivity of KYSE150 cells to celecoxib, whereas ectopic NR4A2 expression substantially augmented the sensitivity of KYSE150 cells to this compound (Fig. 8F). Second, in vivo study demonstrated that oral administration of celecoxib dramatically suppressed the growth of subcutaneous KYSE150 xenograft tumors and downregulated intratumoral NR4A2 levels under high dietary methionine condition (Fig. 8G–J). Of note, celecoxib administration in vivo yielded an overt anti-ESCC effect in the presence of high dietary methionine, comparable to the effect of a low methionine diet or a well-known chemotherapeutic agent cisplatin (CDDP) under high dietary methionine condition (Fig. 8I).

Finally, we analyzed the safety of celecoxib administration in mice harboring KYSE150 xenograft tumors. When compared to high methionine diet, low methionine diet caused a significant reduction in the weight of the bodies, kidneys, and livers, but not the spleens (Supplementary Fig. S8). Similarly, under high dietary methionine condition, cisplatin (CDDP) administration evidently reduced the weight of the bodies, kidneys, livers, and spleens (Supplementary Fig. S8). However, in the presence of high dietary methionine, celecoxib administration only downregulated the weight of the livers and spleens, but not the bodies and kidneys (Supplementary Fig. S8). Notably, in the presence of high dietary methionine, there was a remarkable reduction in the liver weight in CDDP group as relative to celecoxib group (Supplementary Fig. S8C). Collectively, these results demonstrated that the safety of celecoxib administration in vivo was superior to that of a MR diet or CDDP treatment.

## Discussion

Altered metabolism is a core hallmark of cancer and plays a direct role in cancer initiation and progression [6, 47]. Recent studies have reported the impact of diet on cancer growth and progression by modifying cancer cell metabolism [48–50]. Among these, methionine, an essential amino acid for humans, has been found to be pivotal for the expansion of tumor-initiating cells in lung cancer [19]. Additionally, dietary methionine can impinge upon the sensitivity of certain cancer cells, including colorectal cancer and soft-tissue sarcoma cells, to chemotherapy and radiotherapy [25]. In this study, we conducted a tissue metabolic survey in four patient cohorts, providing strong evidence of the aberrantly provoked methionine cycle in clinical ESCC tissues. Furthermore, we observed an inverse correlation between the activity of this metabolic pathway and patient survival. Importantly, our study demonstrated that dietary methionine was readily utilized by ESCC to generate SAM, thereby facilitating ESCC growth. These findings contribute to the growing body of evidence supporting methionine as a tumor-promoting metabolite in ESCC.

As a well-studied epigenetic event, m^6^A modification in mRNA plays a key role in posttranscriptional gene expression regulation. While it is widely accepted that methionine is readily absorbed by various cancer cells to generate the universal methyl donor SAM [17], whether and how methionine alters the RNA m^6^A methylation pattern of ESCC cells remains unclear. In our study, we demonstrated a remarkable upregulation of m^6^A abundance in RNA samples from clinical ESCC tissues via a methionine-SAM-METTL3 cascade. RNA-seq and MeRIP-seq assays revealed substantial changes in gene expression and mRNA m^6^A methylation signatures upon methionine treatment in ESCC cells. Importantly, we identified *NR4A2* as a prominent downstream gene that was positively regulated by methionine through a SAM-METTL3-RNA m^6^A-IGF2BP2 cascade. Notably, we demonstrate that methionine strongly elicits *NR4A2* expression in ESCC cells, but shows negligible influence on *NR4A2* expression in non-transformed esophageal epithelial cells, potentially suggesting that this amino acid does not involve in the tumorigenesis of ESCC, consistent with the previous finding in a nutritional epidemiology study [12]. Furthermore, we found that m^6^A modification in the 3′-UTR of *NR4A2* is important for IGF2BP2-induced stability of *NR4A2* mRNA, accounting for approximately 25.9% of its stability. Therefore, further investigation is needed to ascertain other m^6^A sites contributing to IGF2BP2-mediated stability of *NR4A2* mRNA. In conclusion, methionine addiction exerts a profound influence on the molecular traits of ESCC cells through intricate epitranscriptional regulation.

NR4A2 is a member of nuclear receptor subfamily 4A and its role in solid tumors is still a subject of debate [51]. For instance, *NR4A2* exhibits oncogenic functions in cervical, prostate, and colon cancers by promoting malignant transformation and refraining intrinsic apoptosis [52, 53], while acting as a tumor suppressor in bladder cancer by eliciting cell apoptosis [54]. In our study, we provide evidence supporting *NR4A2* as an oncogene in ESCC, as it boosts cell growth in the presence of methionine and negatively affects patient survival. Considering the adverse effect of a MR diet, such as severe body weight loss observed in this study, NR4A2, a newly identified methionine-responsive oncogene with specifically high expression in clinical ESCC tissues, represents a compelling target for ESCC treatment in the presence of a normal diet. Indeed, celecoxib, an inhibitor targeting NR4A2, shows overt anti-ESCC growth efficacy and safety in the presence of a methionine-rich diet.

In the end, several important issues raised by our study. Firstly, the impact of methionine/SAM on the methylation patterns of various intracellular biological macromolecules demands thorough exploration. SAM, as the sole source of the universal methyl donor, serves as an indispensable substrate in all methylation reactions, encompassing the methylation of RNA, DNA, histones, and non-histone proteins [18]. Secondly, the influence of methionine/SAM on the m^6^A modification of other RNAs necessitates comprehensive assessment. Previous studies report that m^6^A modification is prevalent across various RNAs, encompassing mRNAs, transfer RNAs, ribosomal RNAs, circular RNAs, micro RNAs, and long non-coding RNAs [29, 55]. Thirdly, the mechanisms by which methionine determines the unique mRNA m^6^A methylation pattern in ESCC cells need to be further explored. While METTL3 has been shown to intensify m^6^A modification in adenomatous polyposis coli (*APC*) mRNA in ESCC cells [41], methionine-induced mRNA transcripts with prominent m^6^A peaks do not include *APC* (Supplementary Table S6). Therefore, the underlying mechanism by which methionine triggers a distinct mRNA m^6^A signature in ESCC requires further investigation. Fourthly, the mechanisms through which methionine regulates gene expression in ESCC should be thoroughly addressed. Although Fig. 4 shows upregulation of *GAS7* and downregulation of *HOGA1* in the presence of methionine, no m^6^A peaks were identified in the mRNAs of these genes. Hence, there must be other unknown mechanisms by which methionine modulates the expression of these genes. Fifthly, it is important to carefully determine whether NR4A2 inhibition is a viable alternative to MR for ESCC therapy. We demonstrate that, in a subcutaneous xenograft mouse model, *NR4A2* deletion leads to a significant anti-ESCC effect in the presence of a methionine-rich diet, similar to the effect of a MR diet. In this mouse model, oral celecoxib administration also yields an evident anti-ESCC effect under high dietary methionine condition, comparable to the effect of a MR diet or CDDP under high dietary methionine condition. Further animal models are necessary to confirm whether NR4A2 inhibition, as a new approach that can bypass the adverse effect of MR, can provide considerable therapeutic benefit for ESCC. Sixthly, the tumor-promoting mechanism of *NR4A2* in ESCC warrants urgent investigation, given its paradoxical roles in cancer as reported in previous studies. Ultimately, it is imperative to unravel the impact of other methionine-induced genes, such as *ALDH3B1*, *ANKRD37*, *GAS7*, *SOX9*, and *DUSP5*, on the phenotypic characteristics of ESCC cells in future investigations. Notably, ALDH3B1 is found to be important for glioma cell proliferation [56]. The heightened expression of SOX9 in clinical cancer tissues promotes neoplastic growth of cancer cells [57]. Furthermore, DUSP5, known for its overexpression in human thyroid carcinoma (TC) tissues, plays a pivotal role in fostering TC cell metastasis and anchorage-independent growth [58]. Given these findings, it is imperative to access the involvement of these methionine-responsive genes in the progression of ESCC, thereby advancing our understanding of ESCC pathogenesis.

## Materials and methods

All materials and methods are available in the Supplementary Information file. All the full and uncropped WB images in this paper are uploaded as Supplementary Information.

### Supplementary information

Supplementary information

Supplementary Table S6

Original WB Data File

## Data Availability

All datasets are available from the corresponding author on reasonable request.
