# Supplementary material for: Unveiling the methionine cycle: a key metabolic signature and NR4A2 as a methionine-responsive oncogene in esophageal squamous cell carcinoma
Source: Cell Death Differ. 2024 Apr 3;31(5):558–73. doi: 10.1038/s41418-024-01285-7 (PMC11094133; doi:10.1038/s41418-024-01285-7)
Supplement: Supplementary file 1 — Supplementary information [file 41418_2024_1285_MOESM1_ESM.pdf]

Supplementary Information for

Unveiling the Methionine Cycle: A Key Metabolic Signature and *NR4A2* as a Methionine-Responsive Oncogene in Esophageal Squamous Cell Carcinoma

Xing Jin[1,2#], Lei Liu[3#], Dan Liu[1,2#], Jia Wu[1,2], Congcong Wang[1,2], Siliang Wang[1,2], Fengying Wang[1,2], Guanzhen Yu[4,5], Xiaoxia Jin[6], Yu-Wen Xue[7], Dan Jiang[7], Yan Ni[8], Xi Yang[9], Ming-Song Wang[10], Zhi-Wei Wang[11], Yuriy Orlov[12-16], Wei Jia[17], Gerry Melino[18], Ji-Bin Liu[19], Wen-Lian Chen[1,2*].

[*]Corresponding author. Email: chenwl8412@shutcm.edu.cn (W.-L.C.)

The supplementary file includes:

Supplementary Materials and Methods.

Figure S1. Related to Figure 1. Activity and prognostic significance of the methionine cycle in clinical ESCC tissues of patients.

Figure S2. Related to Figure 1. Increased expression of methionine transporter and metabolic enzymes in clinical ESCC tissues.

Figure S3. Related to Figure 2. Exogenous SAH and Hcy uptake by ESCC cells, influence of methionine and its downstream metabolites on ESCC cell growth and cell cycle progression, and impact of dietary methionine on the body weight of mice.

Figure S4. Related to Figure 3. Comparison of METTL3 expression between ESCC tissues and NATs from validation cohort 3.

Figure S5. Related to Figure 4. Correlation analysis between the gene expression levels in the two different experimental settings, and the influence of methionine on mRNA m^6A methylation of KYSE150 cells.

Figure S6. Related to Figure 5. SAM eliciting NR4A2 expression in ESCC cells via METTL3-mediated RNA m^6A methylation, and construction of the fusion expression of wild-type or mutant 3'-UTR fragment of *NR4A2* with firefly luciferase reporter.

Figure S7. Related to Figure 7. Increased expression of NR4A2 in clinical ESCC tissues.

Figure S8. Related to Figure 8. Side effect analysis of oral celecoxib administration in mice harboring KYSE150 xenograft tumors.

Table S1. Basic characteristics of the discovery cohort, related to Figure 1.

Table S2. Basic characteristics of the validation cohort 1, related to Figure 1.

Table S3. Basic characteristics of the validation cohort 2, related to Figure 1.

Table S4. Basic characteristics of the validation cohort 3, related to Figure S2.

Table S5. The ingredients of high methionine (0.86%), medium methionine (0.35%), and low methionine (0.17%) diets for mouse, related to Figure 2.

Other Supplementary Materials for this manuscript includes the following:

Table S6. (Excel file) RNA transcripts with modified m^6A peaks induced by methionine, related to Figure 4.

Uncropped WB images

Supplementary Materials and Methods

KEY RESOURCES TABLE

REAGENT or RESOURCE	SOURCE	IDENTIFIER
Cell lines, Chemicals, Peptides, and antibodies		
Eca109	SSRCC	JY334
KYSE150	Stem Cell Bank, Chinese Academy of Sciences	TCHu236
KYSE450	Cobier Bioscience	CBP60458
KYSE30	Ek-Bioscience	CC-Y1314
Het-1A	ATCC	CRL-2692
HEK 293T	ATCC	CRL-3216
4-Nitroquinoline 1-oxide	Sigma	N8141
Methionine	MCE	HY-N0326
S-Adenosyl-L-methionine	MCE	HY-W017770
S-Adenosyl-L-Homocysteine	Solarbio	YZ-1012112
L-Homocysteine	MCE	HY-W010347
1,2-Propanediol	Sigma	57-55-6
Methylene blue	Solarbio	G1300
3-Deazaadenosine	MCE	HY-W013332A
Actinomycin D	MCE	HY-17559
STM2457	MCE	HY-134836
Celecoxib	MCE	HY-14398
Parecoxib	MCE	HY-17474A
Rofecoxib	MCE	HY-17372
Methylcellulose	MCE	HY-125861
Cycloheximide	MCE	HY-12320
Tween 80	MCE	HY-Y1891
Cisplatin	MCE	HY-17394
T-Store® Tissue Storage and Transportation Medium	Life science	TSTORE-P
Trizol	Thermo Fisher Scientific	15596018
Polybrene	Beyotime	C0351
Puromycin	Beyotime	ST551
Lipofectamine 3000	Thermo Fisher Scientific	L3000008
Lipofectamine 2000	Thermo Fisher Scientific	11668019
BsmBI	NEB	R0739S
RPMI 1640 medium	Gibco	11875093
RPMI 1640 medium (methionine-free)	BasalMedia	X102J1
penicillin-streptomycin	Gibco	15140122

REAGENT or RESOURCE	SOURCE	IDENTIFIER
Fetal Bovine Serum	Biological Industries	04-001-1ACS
Hind III	Takara	1060
XbaI	Takara	1093
PrimeSTAR® Max DNA Polymerase	Takara	R045
5×Loading buffer	Bio-rad	1610747
Anti-SLC7A5 antibody	proteintech	28670-1-AP
Anti-MAT2A antibody	proteintech	55309-1-AP
Anti-MAT2B antibody	proteintech	15952-1-AP
Anti-AHCY antibody	proteintech	10757-2-AP
Anti-Keratin 14	proteintech	10143-1-AP
Anti-Ki-67	proteintech	27309-1-AP
Anti-m^6A antibody	proteintech	68055-1-Ig
Anti-NR4A2 antibody	proteintech	10975-2-AP
Anti-METTL3 antibody	Cell Signaling Technology	86132
Anti-IGF2BP1 antibody	proteintech	22803-1-AP
Anti-IGF2BP2 antibody	proteintech	11601-1-AP
Anti-IGF2BP3 antibody	proteintech	14642-1-AP
Anti- rabbit-IgG	Cell Signaling Technology	2729
Anti-Cyclin B1	proteintech	55004-1-AP
Anti-PCNA antibody	Cell Signaling Technology	13110
Anti-β-Actin antibody	proteintech	20536-1-AP
Anti-mouse-IgG-HRP antibody	Cell Signaling Technology	7076
Anti-rabbit-IgG-HRP antibody	Cell Signaling Technology	7074
Oligonucleotides (5’→3’)		
CRISPR guide RNA for non-target control (NC):<br>FWD: AAGAAGAATTGGGGATGATG<br>REV: CATCATCCCCAATTCTTCTT	Addgene	N/A
CRISPR guide RNA #1 for human *METTL3*:<br>FWD: CAGACCAGAGACTAACGAAC<br>REV: GTTCGTTAGTCTCTGGTCTG	Addgene	N/A
CRISPR guide RNA #2 for human *METTL3*:<br>FWD: CTTAGATCTACGGAATCCAG<br>REV: CTGGATTCCGTAGATCTAAG	Addgene	N/A
CRISPR guide RNA #1 for human *NR4A2*:<br>FWD: ATTACCTGTATGCTAATCGA<br>REV: TCGATTAGCATACAGGTAAT	Addgene	N/A
CRISPR guide RNA #2 for human *NR4A2*:<br>FWD: TTGTCCTTCGATTAGCATAC<br>REV: GTATGCTAATCGAAGGACAA	Addgene	N/A
CRISPR guide RNA #1 for Human *IGF2BP2*:	Addgene	N/A

REAGENT or RESOURCE	SOURCE	IDENTIFIER
FWD: ACTGGCTTACCTCCCACTGC<br>REV: GCAGTGGGAGGTAAGCCAGT		
CRISPR guide RNA #2 for Human *IGF2BP2*:<br>FWD: ACATCCCTCCTCACCTGCAG<br>REV: CTGCAGGTGAGGAGGGATGT	Addgene	N/A
Primers for full-length coding sequence of human *NR4A2*:<br>FWD:<br>CCCAAGCTTATGGACTACAAGGACGACGATGACAAGCCTTGTGTTCAGGC<br>GCAGTATGG<br>REV: TGCTCTAGATTAGAAAGGTAAAGTGTCCAGGAAA	BioSune	N/A
Primers for *NR4A2*-Mut1:<br>FWD: CTGCTTTTTGAATCAGCTTTCTTAGATCTGTTTGTCCTTCGATTA<br>REV: TAATCGAAGGACAAACAGATCTAAGAAAGCTGATTCAAAAAGCAG	BioSune	N/A
Primers for *NR4A2*-Mut2:<br>FWD: CACCGCCAGCAATAATTGACAAACTTTTCCTGGTCACTTTACCTT<br>REV: AAGGTAAAGTGACCAGGAAAAGTTTGTCAATTATTGCTGGCGGTG	BioSune	N/A
Primers for *NR4A2*-3'-UTR-WT:<br>FWD: TGCTCTAGAGACCTCCTCCCAAGCACTTCAAAGG<br>REV: TGCTCTAGAGTGCCTATAACATTTTCAACCTAGA	BioSune	N/A
Primers for *NR4A2*-Mut3:<br>FWD: CCCAAGCACTTCAAAGGATCTGGAATG<br>REV: CATTCCAGATCCTTTGAAGTGCTTGGG	BioSune	N/A
Primers for qRT-PCR of *NR4A2*:<br>FWD: GTTCAGGCGCAGTATGGGTC<br>REV: CTCCCGAAGAGTGGTAACTGT	BioSune	N/A
Primers for qRT-PCR of *ACTB*:<br>FWD: CATGTACGTTGCTATCCAGGC<br>REV: CTCCTTAATGTCACGCACGAT	BioSune	N/A
Critical Commercial Assays		
BCA Assay Kit	Thermo Scientific	23225
PrimeScript RT reagent Kit	Takara	RR047
FITC Annexin V Apoptosis Detection Kit	BD	556547
Cell Cycle Analysis Kit	Beyotime	C1052
Cell Counting Kit-8(CCK-8)	ShareBio	SB-CCK8
Ultrapure RNA Kit	CWBIO	CW0581
SYBR Premix Ex Taq Kit	Takara	RR390
NEBNext Poly(A) mRNA Magnetic Isolation Module	NEB	E7490
NEBNext rRNA Depletion Kit	NEB	E6310
Methylated RNA immune-precipitation (MeRIP) m^{6}A kit	GenSeq®	GS-ET-001
QuickMutation™Plus Site-Directed Mutagenesis Kit	Beyotime	D0208S

REAGENT or RESOURCE	SOURCE	IDENTIFIER
Promega Dual-Luciferase reporter system	Promega	E1910
RNA Immunoprecipitation (RIP) kit	GenSeq®	GS-ET-006
Experimental Models: Organisms/Strains		
Mouse: BALB/c-nude	Shanghai SLAC Laboratory Animal Co,Ltd	N/A
Mouse: C57BL/6	Shanghai SLAC Laboratory Animal Co,Ltd	N/A
Recombinant DNA		
Lenti-CRISPR-V2	Addgene	52961
psPAX2 packaging vector	Addgene	12260
pMD2.G packaging vector	Addgene	12259
pGL3-Promoter Vector	Promega	E1761
Software and Algorithms		
R language	Open source	https://www.r-project.org/
ImageJ software	Open source	https://imagej.nih.gov/ij/
CRISPR Guide RNA database	Open source	www.addgene.org/
Autodock	Open source	autodock.scripps.edu/

RESOURCE AVAILABILITY

Lead contact

Further information and requests for resources should be directed to and will be fulfilled by the lead contact, Wen-Lian Chen (chenwl8412@shutcm.edu.cn).

Materials availability

Materials and reagents used in this study are listed in the key resources table. Plasmids generated in this study are available from the lead contact upon request.

Data and code availability

- This paper reanalyzes the metabolomic and proteomic data of an ESCC discovery cohort which we reported previously [1]. The raw data are available at https://www.biosino.org/node with

accession numbers OEP002347 for metabolomic dataset and OEP002405 for proteomic dataset.

- RNA-seq and MeRIP-seq data generated by this study are deposited to the National Omics Data Encyclopedia (NODE) database (https://www.biosino.org/node) at Bio-Med Big Data Center affiliated with Shanghai Institute of Nutrition and Health, Chinese Academy of Sciences, with an accession number of OEP004978.
- This paper does not report original code.
- Any additional information required to reanalyze the data reported in this paper is available from the lead contact upon request.

EXPERIMENTAL MODEL AND SUBJECT DETAILS

Patient cohorts

We enrolled four ESCC cohorts into this study, including a discovery cohort, which we reported previously [1], and three validation cohorts, from the Affiliated Tumor Hospital of Nantong University (ATHNU) (Nantong, China) at different time periods: from 2013 to 2015 for validation cohort 1 (n = 94), from 2019 to 2020 for validation cohort 2 (n = 112), and from 2019 to 2022 for validation cohort 3 (n = 41). All patients were newly diagnosed and received no prior treatment for their disease. Notably, all participants provided informed written consent in accordance with the regulation of the Institutional Review Board of the ATHNU (Approval number 2019-022) in agreement with the Declaration of Helsinki.

The following pretreatment examinations were conducted for each patient: neck, thorax, and abdomen plain and contrast-enhanced computed tomography; and esophagogastroduodenoscopy, with ultrasound endoscopy. Optional examinations included bronchoscopy, which was used to

exclude tumor infiltration into the trachea or bronchial tree, positron emission tomography, and radionuclide bone imaging.

Disease staging was carried out according to the American Joint Committee on Cancer 8th edition staging system and the Comprehensive Cancer Network (NCCN) Guidelines for Esophageal and Esophagogastric Junction Cancers [2-4], while treatment protocol for each patient was established by referring to the NCCN Guidelines for Esophageal and Esophagogastric Junction Cancers [3-5].

Cellular models

Human ESCC cell lines (KYSE150, Eca109, KYSE450, and KYSE30), non-transformed esophageal epithelial cell line Het-1A, and HEK 293T were enrolled into this study. All cell lines were authenticated using short tandem repeat (STR) profiling. Of note, all cell lines (and their derivatives) were maintained in RPMI 1640 with 10% fetal bovine serum (FBS), 100 U/mL penicillin, and 100 mg/mL streptomycin at 37 ℃ in 5% CO_2.

***Ex vivo* culture model using primary tissues from patients**

In the ATHNU, when the patients with ESCC underwent esophagectomy, their fresh and primary tissues were quickly harvested and stored in the T-Store® Tissue Storage and Transportation Medium at room temperature (RT). These fresh tissues were immediately sent to the Cancer Institute, Longhua Hospital (CILH, Shanghai, China) at RT for *ex vivo* culture. The *ex vivo* culture model was established as we previously described [6]. In brief, fresh tissues from patients were washed twice in phosphate buffered saline (PBS) solution, cut, and weighed. Subsequently, the acquired tissue segments were cultured in RPMI 1640 medium with or without methionine, as indicated in the

corresponding figures or figure legends.

Mouse models

A subcutaneous xenograft ESCC model and an orthotopic ESCC model induced by a chemical carcinogen 4-nitroquinoline 1-oxide (4-NQO) were recruited in this study. The subcutaneous xenograft tumor model was established using female BALB/c-nude mice, while the orthotopic tumor model was constructed using female C57BL/6 mice. All mice were housed in well-ventilated cages at a constant temperature and humidity (23 ℃ ± 2 ℃, 50–60%) in a specific pathogen-free (SPF) controlled environment, with a standard 12-12 h light-dark cycle, and food and water were provided *ad libitum*. The weight and signs of diseases were measured in regular intervals. Subcutaneous tumor xenograft experiments were approved by the Institutional Animal Care and Use Committee of Shanghai University of Traditional Chinese Medicine (Approval number SYXK-Hu-2020-0009). Studies of carcinogen-induced ESCC mice were approved and conducted by Wuhan Servicebio Technology Co., Ltd (Approval number 2018015). There were at least 5 mice in each group (n = 5).

METHOD DETAILS

Acquisition of clinical specimens and clinical data

In the ATHNU, ESCC tumor tissues and paired normal adjacent tissues (NATs) were harvested from patients of four cohorts when they underwent esophagectomy. Tissue samples were either flash frozen in liquid nitrogen and then transferred to -80 ℃ freezer for long-term storage, or formalin-fixed paraffin-embedded for hematoxylin and eosin (H&E) staining and tissue microarray preparation. The H&E slides of each patient were independently reviewed by three board-certificated

pathologists to assess the pathological characteristics. Acceptable ESCC tissue segments were determined by the percent viable tumor nuclei (> 80%), total cellularity (> 50%) and necrosis (< 20%).

Clinical characteristics of each patient were carefully reviewed and recorded. Overall survival (OS) and disease-free survival (DFS) were obtained for validation cohort 1 and validation cohort 2. OS was computed using the time from the date of surgery to the date of death or the last follow-up. DFS was calculated using the time from the date of resection to the date of disease recurrence or death.

Cell culture

Cells were cultured in standard RPMI 1640 or methionine-free RPMI 1640 as indicated in the figures or figure legends, with 10% fetal bovine serum (FBS) or 10% dialyzed FBS (dFBS), 100 U/mL penicillin, and 100 mg/mL streptomycin at 37 °C in 5% CO_2. Methionine, SAM, SAH, or Hcy was supplemented into the methionine-free RPMI 1640 as demonstrated in the figures or figure legends.

***Ex vivo* culture of primary tissues derived from patients**

Ex vivo culture of fresh and primary tissues from patients was performed following the protocol which we depicted previously [6]. When the fresh tissues were received, they were washed twice in PBS solution, cut, and weighed. The obtained tissue segments were cultured in methionine-free RPMI 1640 medium with 10% FBS, 100 U/mL penicillin, and 100 mg/mL streptomycin at 37 °C in 5% CO_2 for 24 hours. Methionine, SAM, SAH, or Hcy was supplemented as indicated in the corresponding figures or figure legends.

Subcutaneous xenograft tumor mouse model

For the establishment of subcutaneous xenograft model, KYSE150, Eca109, or their derivatives were resuspended in PBS buffer at a density of 5×10^7 cells/mL. Subsequently, 100 μL cell suspension solution containing 5×10^6 cells was subcutaneously injected into the right flank of eight-week-old female BALB/c nude mice. For the investigation of the influence of dietary methionine on subcutaneous tumor growth, mice were randomly grouped as indicated in figures or figure legends after 7 days of injection. Customized diets containing distinct dosage of methionine (weight/weight), including 0.86% methionine (high dietary methionine), 0.35% methionine (medium dietary methionine), or 0.18% methionine (low dietary methionine) (Table S5), were orally administered to the corresponding groups.

For the investigation of the effect of celecoxib and cisplatin (CDDP) on xenograft tumor growth under high dietary methionine condition, these chemical compounds were administered at day 10 after ESCC cell implantation using the following protocols: (1) celecoxib was prepared as a suspension in 0.5% methylcellulose (w/v) and 0.1% Tween 80 (v/v) in sterile water and orally administered to the mice at a dose of 25 mg/kg/day until the mice were killed; (2) CDDP was dissolved in saline 0.9% solution at a dose of 2 mg/kg and intraperitoneally administered every two days until the mice were killed.

Subcutaneous tumors were measured every three or four days using caliper. The volumes of tumors were calculated using the formula $0.5 \times \text{length} \times \text{width}^2$. Mice were sacrificed three weeks after cell inoculation. Xenograft tumors were harvested for imaging and weighing. Subsequently, tumor tissues were flash frozen in liquid nitrogen and stored at -80 ℃ for further analysis.

Carcinogen-induced ESCC mouse model

An orthotopic ESCC mouse model was established by orally administering a well-known chemical carcinogen 4-nitroquinoline 1-oxide (4-NQO) to six-week-old C57BL/6 female mice as we previously illustrated [1, 7]. Briefly, 4-NQO stock solution was prepared in propylene glycol at 6 mg/mL and divided into aliquots for storage at -20 ℃. Model group was constructed by orally administering drinking water containing 4-NQO at a concentration of 100 μg/mL *ad libitum* for 16 weeks. Subsequently, model mice were randomly divided into two subgroups: one subgroup was provided with pure water *ad libitum* for 12 weeks (ESCC mice), while the other subgroup was supplied with water containing 10 mg/mL methionine *ad libitum* for 12 weeks (ESCC mice with Met admin). Healthy control group was established by orally administering drinking water containing 1.67% propylene glycol (vol/vol) *ad libitum* for 16 weeks and then pure drinking water for 12 weeks. At the end of the experiment, all mice were sacrificed and their esophageal tissues were collected and formalin-fixed paraffin-embedded for further analysis.

Cell viability and cell proliferation analysis

Cells were cultured in standard or methionine-free RPMI 1640 medium, with 10% FBS, 100 U/mL penicillin, and 100 mg/mL streptomycin at 37 ℃ in 5% CO_2. At the time points of cell culture as indicated in the figures or figure legends, cell viability and proliferation were measured using a Cell Counting Kit-8 (CCK-8) following the manufacturer’s instruction.

Cell cycle assay

The following protocol was used for cell cycle assay. Cells were digested with trypsin (0.25%) and

washed in PBS buffer for three times. Subsequently, each cell sample was resuspended in 200 μL PBS buffer. Next, cells were fixed in ice-cold 70% ethanol for 24 hours. After incubating with propidium iodide (PI)/RNase staining buffer for 30 minutes at RT, cell cycle feature of each sample was measured using a flow cytometer (Beckman Coulter, DxFLEX) and analyzed using the FlowJo software.

Hematoxylin & eosin and immunohistochemistry staining

Esophageal tissue samples from model mice, which were formalin-fixed paraffin-embedded, were employed for hematoxylin & eosin (H&E) and immunohistochemistry (IHC) staining as we previously depicted [1, 7, 8]. IHC staining was performed using antibodies against keratin-14, Ki-67, and NR4A2.

Tissue specimens from three validation cohorts were used to construct tissue microarrays as we previously described [1, 6-9]. Subsequently, tissue microarrays were stained with antibodies against SLC7A5, MAT2A, MAT2B, AHCY, METTL3, and NR4A2. Quantitative staining scores of tissue sections were determined by the percentage of positive cells and staining intensity. The range for percentage of positive cells was from 0% to 100%. While the staining intensity was defined as 0 for negative intensity, 1 for weak intensity, 2 for moderate intensity, and 3 for strong intensity. The normalized staining score of each antibody of each sample was acquired by multiplying the proportion value of positive cells and the intensity score of the tissue section.

Western blot assay

Supernatants of cell/tissue lysates were collected through centrifugation at 12,000 × g for 10 minutes.

The concentrations of the supernatants containing total proteins were determined using a BCA Assay Kit. Protein extracts were denatured by addition of sample loading buffer followed by boiling for 10 minutes. Next, proteins were separated by SDS-PAGE, and then transferred to a PVDF membrane. After incubation with primary antibodies overnight at 4 °C, the membranes were washed and incubated with secondary antibodies. Western blot images were captured using a ChemiDocTouch Imaging System (Bio-rad).

Quantitative reverse transcription-PCR

Cell or tissue samples were lysed in Trizol reagent and total RNAs were then extracted using an Ultrapure RNA Kit according to the manufacturer's recommendations. RNAs were reversely transcribed to complementary DNA (cDNA) using a PrimeScript RT reagent Kit. Quantitative reverse transcription-PCR (qRT-PCR) assay was performed with a SYBR Premix Ex Taq Kit on an Applied Biosystems Q5 PCR machine (Applied Biosystems, Foster City, CA, USA).

m^6A dot blot

Total RNAs were used for m^6A dot blot assay. For cellular RNAs, cells were lysed in the Trizol reagent and total RNAs were extracted using an Ultrapure RNA Kit according to the manufacturer's instructions. For tissue RNAs, clinical tissue samples were cut into small pieces and homogenized in the Trizol reagent on ice. Total RNAs of tissues were acquired by using the Ultrapure RNA Kit.

The concentrations of acquired RNA samples were determined by the NanoDrop machine. All RNA samples were diluted to 400 ng/μL in RNase-free water. Next, RNA samples were denatured at 72 °C to disrupt their secondary structures, followed by chilling on ice immediately. Subsequently, 1

μL purified RNA of each sample was dropped onto the nitrocellulose membrane, followed by crosslinking in a Stratalinker 2400 UV Crosslinker in the Autocrosslink mode (1,200 microjoules, 25-50 seconds). After being blocked in 5% non-fat milk solution, the membranes were incubated with anti-m^6A antibody overnight at 4 °C. Finally, the membranes were washed in TBST buffer three times and incubated with IgG-HRP. Dot blot images were captured by using a ChemiDocTouch Imaging System (Bio-rad).

Gene knockout by CRISPR-Cas9 approach

Two human ESCC cell lines, KYSE150 and Eca109, were used for gene knockout studies according to our previous protocols [1, 6, 8, 9]. Briefly, the guide RNA (gRNA) of each gene was inserted into a lenti-CRISPR-V2 plasmid which was digested by the BsmBI endonuclease. The lentivirus was packed as follows: lenti-CRISPR-V2 vector containing nontarget control (NC) gRNA or gRNAs of target genes was co-transfected with psPAX2 and pMD2.G plasmids into HEK 293T cell using the Lipofectamine 3000 reagent according to the manufacturer's recommendations. After 48 hours of cell culture, the lentivirus supernatant was collected by centrifugation at 3,000 × g and filtered using a 0.45 μm filter in order to thoroughly remove HEK 293T cells. Subsequently, ESCC cells were infected with the NC lentivirus or lentivirus expressing target gRNAs in the presence of 8 μg/mL polybrene. Infected ESCC cells were screened with an antibiotic, puromycin, for 48 hours. Of note, the following gRNAs were used in this study.

gRNA of NC: 5'-AAGAAGAATTGGGGATGATG-3';

gRNA#1 for *METTL3*: 5'- CAGACCAGAGACTAACGAAC-3';

gRNA#2 for *METTL3*: 5'- CTTAGATCTACGGAATCCAG-3';

gRNA#1 for *NR4A2*: 5'- ATTACCTGTATGCTAATCGA-3';

gRNA#2 for *NR4A2*: 5'- TTGTCCTTCGATTAGCATAC-3';

gRNA#1 for *IGF2BP2*: 5'- ACTGGCTTACCTCCCACTGC-3';

gRNA#2 for *IGF2BP2*: 5'- ACATCCCTCCTCACCTGCAG-3'.

NR4A2 overexpression

The wild-type (WT) full-length coding sequence of *NR4A2* was cloned by a pair of primers (HindIII-Flag-NR4A2-forward primer: 5'-CCCAAGCTTATGGACTACAAGGACGACGATGACAAGCCTTGTGTTCAGGCGCAGTATGG-3'; XbaI-NR4A2-reverse primer: 5'-TGCTCTAGATTAGAAAGGTAAAGTGTCCAGGAAA-3') when using the cDNA from KYSE150 cells as the template in the presence of the PrimeSTAR® Max DNA Polymerase. The sequence was then inserted into the pcDNA3.0 vector, which was pre-treated with HindIII and XbaI. Next, the negative control vector pcDNA3.0 and the recombinant vector pcDNA3.0-Flag-NR4A2 were transfected into ESCC cells, respectively, according to the manufacturer's instructions. The transfection was performed using a Lipofectamine 3000 Transfection Reagent following the manufacturer's protocol.

RNA stability assay

ESCC cells were cultured in distinct media containing 5 μg/mL actinomycin D for 0 hour, 4 hours, and 8 hours, respectively. Cells were lysed in the Trizol reagent and total RNAs were extracted using the Ultrapure RNA Kit. Quantitative RT-PCR was conducted to measure the remaining levels of *NR4A2* mRNA in cells, therefore assessing the RNA stability of *NR4A2* under different treatment

conditions.

RNA immunoprecipitation assay

RNA Immunoprecipitation assay (RIP) was performed using an RIP kit. Briefly, KYSE150 cells were lysed in the RIP lysis buffer. The supernatant of the RIP lysate was acquired and incubated with anti-IGF2BP1, anti-IGF2BP2, anti-IGF2BP3, or anti-IgG antibodies in the presence of protein A/G magnetic beads under the condition of continuous shaking at 4 ℃ overnight. The co-immunoprecipitated RNA samples were then purified with wash buffer, followed by incubation in 100% ethanol at -80 ℃ overnight. Input and co-immunoprecipitated RNAs were analyzed by qRT-PCR. Finally, the enrichment of *NR4A2* by each antibody was calculated by normalizing to the input *NR4A2*.

Site-directed mutagenesis

Wild-type full-length coding sequence of *NR4A2* (*NR4A2*-WT) was cloned using PrimeSTAR® Max DNA Polymerase and inserted into pcDNA3.0 vector according to manufacturer's instruction. Two *NR4A2* mutants with A-T substitution in exon 6 (*NR4A2*-Mut1) and exon 8 (*NR4A2*-Mut2), respectively (shown in Figure 5K), were generated using the QuickMutation™Plus Site-Directed Mutagenesis Kit. Briefly, different primer pairs and *NR4A2*-WT-pcDNA3.0 as a template were mixed to perform PCR using BeyoAmp™ Extra-long DNA Polymerase and BeyoAmp™ Buffer. PCR products were digested with DpnI enzyme and then inserted into the pcDNA3.0 vector. The recombinant plasmids were transformed into *E.coli DH5α* competent cells for amplification and sequencing. The primer pairs used for cloning were listed as follows.

Primer pair for *NR4A2*-WT:

Forward

5'-CCCAAGCTTATGGACTACAAGGACGACGATGACAAGCCTTGTGTTCAGGCGCAGTATGG-3';

Reverse 5'-TGCTCTAGATTAGAAAGGTAAAGTGTCCAGGAAA-3';

Primer pair for *NR4A2*-Mut1:

Forward 5'-CTGCTTTTTGAATCAGCTTTCTTAGATCTGTTTGTCCTTCGATTA-3';

Reverse 5'-TAATCGAAGGACAAACAGATCTAAGAAAGCTGATTCAAAAAGCAG-3';

Primer pair for *NR4A2*-Mut2:

Forward 5'-CACCGCCAGCAATAATTGACAAACTTTTCCTGGTCACTTTACCTT-3';

Reverse 5'-AAGGTAAAGTGACCAGGAAAAGTTTGTCAATTATTGCTGGCGGTG-3'.

Dual-luciferase reporter assay

Wild-type 3'-UTR fragment of *NR4A2* was cloned using the primer pair of *NR4A2*-3'-UTR-WT in the presence of PrimeSTAR® Max DNA Polymerase. By using *NR4A2*-3'-UTR-WT as the template, a mutant 3'-UTR of *NR4A2* (*NR4A2*-Mut3) with A-T substitution as shown in Figure 5K was synthesized using a QuickMutation™Plus Site-Directed Mutagenesis Kit according to the manufacturer's instructions. Furthermore, the promoter sequence of *NR4A2* was cloned using the primer pair of *NR4A2*-promoter. To generate the firefly luciferase (F-luc) reporter construct, *NR4A2*-3'-UTR-WT, and *NR4A2*-Mut3 were inserted into the XbaI site of the pGL3-Promoter luciferase reporter plasmid, respectively. While *NR4A2*-promoter was inserted into PGL3-basic luciferase reporter plasmid. Next, 50 ng recombinant luciferase reporter plasmid with

NR4A2-3'-UTR-WT, *NR4A2*-Mut3, or *NR4A2*-promoter and 50 ng TK-Renilla luciferase (R-luc) control plasmids were co-transfected into HEK 293T cells using a Lipofectamine 2000 kit. F-luc and R-luc activities were measured 48 hours later with the Promega Dual-Luciferase reporter system (Promega, cat#E1910) according to the supplier's instructions. The relative luciferase activity was computed by dividing F-luc by R-luc and normalized to individual control for each assay. The primer pairs used for cloning were listed as follows.

Primer pair for *NR4A2*-3'-UTR-WT:

Forward 5'-TGCTCTAGAGACCTCCTCCCAAGCACTTCAAAGG-3';

Reverse 5'-TGCTCTAGAGTGCCTATAACATTTTCAACCTAGA-3';

Primers for *NR4A2*-Mut3:

Forward 5'-CCCAAGCACTTCAAAGGATCTGGAATG-3';

Reverse 5'-CATTCCAGATCCTTTGAAGTGCTTGGG-3';

Primers for *NR4A2*-promoter:

Forward 5'-CGGGGTACCTCCTTCTCCCCAGCTCTCCAAA-3';

Reverse 5'-CCCAAGCTTGGCTGGACAGGCAAAAGGGA-3'.

Molecular docking

The structure file of NR4A2 protein (ID: 5Y41) was acquired from Protein Data Bank, while the structure of celecoxib was obtained from PubChem (CAS ID: 169590-42-5). NR4A2 and celecoxib were designated as the receptor and ligand, respectively, to conduct molecular docking using Autodock software (vision 4.2.6). The docking box encompassed the entire protein as well as the ligand-receptor docking structures of NR4A2. The genetic algorithm was employed with 50 semiflexible docking iterations. Other parameters of Autodock were

assigned at default settings. Subsequently, the LamarckianGA (4.2) option was selected to generate the docking parameter file before executing Autodock program. Finally, the docking result was visualized by using PyMOL (vision 2.5).

Cellular thermal shift assay (CETSA)

The CETSA was performed as previously illustrated [10]. In brief, ESCC cells were incubated with vehicle or celecoxib (40 μM) for 3 hours, respectively. Cell precipitations were then acquired by digestion with 0.5% trypsin. After washing twice with PBS, the cell precipitations of each group were divided into 5 aliquots in PBS buffer. All cell suspensions were frozen at -80 ℃ and thawed for three times, then sequentially incubated at 37 ℃, 42 ℃, 47 ℃, 52 ℃, and 57 ℃ for 3 minutes in a thermal cycler, respectively. Subsequently, all cell suspensions were centrifuged at a speed of 12,000g at 4 ℃ for 10 minutes to obtain cell supernatants. NR4A2 protein levels in all samples were assessed by using western blot assay.

Untargeted metabolomic profiling

Clinical tissue samples from the discovery cohort were measured using the Pegasus High-Throughput Gas Chromatography with Time-of-Flight Mass Spectrometer system as we previously described [1]. While clinical tissue samples from validation cohort 1 and validation cohort 2 were examined using the Ultra High Performance Liquid Chromatography (1290 Infinity LC, Agilent Technologies) coupled to a tandem Mass Spectrometer system (TripleTOF 6600, AB Sciex) (UHPLC-MS/MS) by APT Biotechnology Company (Shanghai, China).

Sample preparation A total of 206 pairs of ESCC and normal adjacent tissues from validation cohort

1 (n = 94) and cohort2 (n = 112) were enrolled for metabolomic profiling. Sample preparation was conducted as previously illustrated [11]. Briefly, tissues were cut to pieces on dry ice and then homogenized in ultrapure water. Subsequently, for each tissue sample, 200 μL supernatant was collected and thoroughly mixed with 800 μL metabolite extraction solution (methanol/acetonitrile, 1:1, vol/vol), followed by centrifugation at a speed of 14,000 × g at 4 °C for 20 minutes. Next, the acquired supernatant was dried in vacuum centrifuge. Finally, the residual was re-dissolved in 100 μL solvent (acetonitrile/water, 1:1, vol/vol), centrifuged at a speed of 14,000 × g at 4°C for 20 minutes, and then injected for UHPLC-MS/MS analysis.

UHPLC-MS/MS Samples were separated in a hydrophilic interaction chromatography (HILIC) column (2.1 mm × 100 mm ACQUIY UPLC BEH Amide 1.7 µm; Waters, Ireland) installed in the ultra-high performance liquid chromatography system. For each sample, 2 μL volume was injected into the column at 25°C and at a flow rate of 0.5mL/min. The mobile phase, consisting of buffer A (25 mM ammonium acetate and 25 mM ammonia hydroxide dissolved in ultrapure water) and buffer B (acetonitrile), was employed in both ESI positive and negative modes. The gradient elution procedure was set as follows: 95% buffer B for 0.5 min, linearly reduced to 65% buffer B in 6.5 min, reduced to 40% buffer B in 1 min and kept for 1 min, and then increased to 95% buffer B in 0.1 min, with a 3 min re-equilibration period employed.

The ESI source conditions were set as follows: ion source gas 1 as 60, ion source gas 2 as 60, curtain gas as 30, source temperature at 600 °C, and ionspray voltage floating at ± 5,500 V. In MS only acquisition, the instrument was set to acquire over the m/z range from 60 Da to 1,000 Da, and the accumulation time for TOF MS scan was set at 0.20 s/spectra. In auto MS/MS acquisition, the instrument was set to acquire over the m/z range from 25 Da to 1,000 Da, and the accumulation time

for product ion scan was set at 0.05 s/spectra. The product ion scan was obtained using information dependent acquisition with high sensitivity mode selected. The parameters were set as follows: the collision energy at 35 V with ± 15 eV, de-clustering potential as 60 V (+) and −60 V (−), exclude isotopes within 4 Da, and candidate ions to monitor per cycle at 10.

Targeted metabolomic measurement

Tissue preparation Frozen tissue samples stored in liquid nitrogen were taken out and accurately weighed on dry ice. For each tissue sample, approximately 50 mg tissue was taken and homogenized in 500 μL organic mixture [Formic acid (FA):H_2O:Acetonitrile (ACN):Methyl alcohol (MeOH), 0.2:20:40:40, vol/vol/vol/vol] for metabolite extraction. The supernatant was acquired by centrifugation at a speed of 13,000 rpm at 4 °C, and then stored at -80 °C for further analysis.

Medium and cell preparation For metabolite extraction of cell and medium samples, a mixture of FA:ACN:MeOH (0.2:50:50, vol/vol/vol) and a mixture of FA:H_2O:ACN:MeOH (0.2:20:40:40, vol/vol/vol/vol), which were pre-cooled at -80 °C overnight, were used as the precipitating and quenching reagents, respectively. For each culture medium sample, 200 μL of medium was added into EP tube containing 800 μL pre-cooled FA:ACN:MeOH (0.2:50:50, vol/vol/vol) and promptly centrifugated at 13,000 rpm at 4°C for 15 minutes. Subsequently, 800 μL supernatant was acquired and quickly frozen at -80 °C for further analysis. For the preparation of each cell sample, spent medium was discarded and culture dishes containing cells were quickly washed three times with PBS buffer. Next, 1 mL of pre-cooled FA:H_2O:ACN:MeOH (0.2:20:40:40, vol/vol/vol/vol) was added into each dish for rapid quenching and precipitating cell samples. After incubating at 4 °C for 15 minutes, cells were scraped down and then lysed by ultrasonication. Of note, during ultrasonication,

samples were cooled for 10 seconds on ice after every 10 seconds of ultrasonication to avoid overheating. After 6 cycles of ultrasonication, lysate in culture dish was transferred into a new EP tube and centrifugated at 13,000 rpm at 4 ℃ for 15 minutes. The acquired supernatant was stored at -80 for further analysis.

Standard curves for quantitative measurement of target metabolites Standard curves were established for quantitative measurement of metabolites of interest, including methionine, SAM, SAH, and Hcy. To evaluate the linearity of the solutions of each standard compound, the standard curve was constructed by using the concentration ratios between the compound and its corresponding internal control over concentration gradient as horizontal coordinate and peak area ratios between the compound and its corresponding internal control over concentration gradient as vertical coordinate, and then the correlation coefficient value was calculated. Notably, correlation coefficient values for all standard curves were larger than 0.99, indicating good linearity of these curves.

UHPLC-MS Samples were separated and analyzed using an ultra-high performance liquid chromatography (UHPLC) (Shimadzu LC-30AD system, Shimadzu Technologies) coupled to a Triple Quad 5500 Mass Spectrometer (Sciex, Applied Biosystems) by Shanghai BioNext Technologies Company (Shanghai, China). A Waters ACQUITY UPLC ®BEH Amide 1.7 μm 2.1 × 50 mm column was installed in the UHPLC. The composition of the mobile phase was listed as follows: solution A as a water/acetonitrile solution containing 0.1% formic acid and 1 mM ammonium acetate (H_2O/ACN, 95:5, vol/vol) and solution B as an acetonitrile/water solution containing 0.1% formic acid and 1 mM ammonium acetate (ACN/H_2O, 95:5, vol/vol). For each sample, 2 μL volume was injected into the column at 40 ℃. The flow rate was set at 400 μL/min. The gradient elution procedure was set as follows: 0-4 min, solution B was linearly reduced to 60%

from 95%; 4-5 min, buffer B was reduced to 60% from 55%; 5.0-5.1 min, buffer B was reduced to 50% from 55%; 5.1-5.6 min, buffer B was kept at 50%; 5.6-5.7 min, buffer B was increased to 95% from 50%; 5.7-6.5 min, buffer B was kept at 95%. To detect and assess the stability and repeatability of the measurement system, a set of standard curves were set at the beginning and the end of the experiment, and these standard chemical samples were also used as quality controls. The standard compounds in standard curves were used to correct the retention time of the chromatography.

Mass spectrometry analysis was performed on a Triple Quad 5500 instrument (Scienx, Applied Biosystems) with ESI ion source in positive mode. The mass spectrometry parameters were set as follows: source temperature at 500 °C, ion source gas 1 at 20 psi, ion source gas 2 at 20 psi, curtain gas at 25 psi, ionspray voltage floating at -5,500 V. Then, the ion pairs were detected using multiple reaction monitoring mode. The Q1/Q3 ion pairs for the metabolites of interest were listed as follows: methionine, 150.2/132.9; SAM, 399.2/250.2; SAH, 385.3/133.6; Hcy, 136.1/55.8.

Peak area and retention time for each target metabolite were extracted using the Analyst software (Applied Biosystems Sciex). Standard chemical compounds were used to correct the retention time of peaks and identify metabolites.

RNA-sequencing

RNA-sequencing (RNA-seq) assay was performed by Cloud-Seq Biotech Inc. (Shanghai, China) following our previous procedure [6]. Total RNAs were extracted from fresh frozen tissues using the Trizol reagent and purified by removing rRNAs with a NEBNext® rRNA Depletion Kit following the manufacturer's instructions. All RNA analytes were assessed for fragment size, integrity, purity and concentration. Subsequently, RNA libraries were constructed using a NEBNext® Ultra™ II

Directional RNA Library Prep Kit according to the manufacturer's instructions. Libraries were controlled for quality and quantified using the Bioanalyzer 2100 system (Agilent Technologies, Inc., USA). Library sequencing was performed on an illumina Hiseq 4000 sequencer with 150 bp paired end mode.

MeRIP-sequencing

Cellular mRNAs were extracted using a NEBNext Poly(A) mRNA Magnetic Isolation Module and a NEBNext rRNA Depletion Kit. Fragmented RNAs (~200 nucleotides) were incubated with m^6A antibody for immunoprecipitation using a methylated RNA immunoprecipitation (MeRIP) m^6A kit according to the supplier's instructions. Both the input mRNAs without immunoprecipitation and the m^6A-immunoprecipitated mRNAs containing m^6A peaks were used for library construction using a NEBNext Ultra RNA library prep kit, and then sequenced with an Illumina NovaSeq instrument with 150bp paired-end reads by Cloud-Seq Biotech Ltd. Co. (Shanghai, China).

QUANTIFICATION AND STATISTICAL ANALYSIS

Untargeted metabolomic data analysis

The raw data were converted to mzXML files using ProteoWizard software, and then imported into freely available XCMS software to pick peaks. During the calculation, parameters were set as follows: centWave m/z = 10 ppm, peakwidth = c (10, 60), prefilter = c (10, 100). For peak grouping, bw = 5, mzwid = 0.025, minfrac = 0.5. The R package, CAMERA (Collection of Algorithms of MEtabolite pRofile Annotation), was applied for annotating isotopes and adducts. In the extracted ion features, only the variables having more than 50% of nonzero measurement values in at least one

group were kept. Metabolites were identified by comparing the accuracy m/z value (<10 ppm) and MS/MS spectra of peaks with an in-house database established with available authentic chemical standards. Heatmaps of metabolites between two groups were generated using the R package ComplexHeatmap [12].

RNA-sequencing data analysis

The quality of raw reads was assessed by Q30 values. After removing sequencing adaptors, short reads (length < 35 bp) and low-quality reads with software of Cutadapt (v1.18) and Trimmomatic (v0.35), high-quality clean reads were obtained. Subsequently, by using HISAT2 software (v2.1.0), clean reads of human data were aligned to the human genome (assembly GRCh38). For the raw count data of genes, the R package edgeR (v3.26.8) [13] was used to filter lowly expressed items and to execute normalization to get reads per kilobase per million (RPKM) expression values. Differentially expressed mRNAs were identified by the cuts of Benjamini-Hochberg adjusted $p < 0.05$ and fold change (FC) > 2 or FC < -2.

MeRIP-sequencing data analysis

Paired-end reads were harvested from Illumina NovaSeq 6000 sequencer. Data quality was evaluated using Q30 values. The 3'-adaptors were trimmed and low-quality reads were removed using cutadapt software (v1.9.3). Clean reads of all libraries were aligned to the reference genome (GRCh37/hg19 at http://genome.ucsc.edu/cgi-bin/hgGateway) by Hisat2 software (v2.0.4). Subsequently, methylated sites on RNAs (peaks) and differentially methylated sites were identified by MACS and diffReps software, respectively. Differentially methylated RNA sites were identified by the cuts of $p < 0.005$

and FC > 2 or FC < -2. The differential peaks, overlapping with exons of mRNAs, were figured out and selected by home-made scripts.

Differential abundance scores of metabolic pathways

First, all measured metabolites were mapped onto KEGG pathways. After determining which metabolites were significantly increased/decreased in abundance, differential abundance (DA) scores of metabolic pathways were calculated using an algorithm as depicted previously [14]. DA captures the tendency for a pathway to have increased/decreased levels of metabolites, compared to a control group. DA score is defined as DA = (No. of metabolites increased － no. of metabolites decreased) / No. of measured metabolites in pathway.

Prognosis-risk score of the methionine cycle metabolites

To evaluate the association between the methionine cycle activity and ESCC patient prognosis, four intratumoral metabolites (homocysteine, SAM, methionine, and SAH) from validation cohort 1 and validation cohort 2, which are involved in the methionine cycle and presented in Figure 1D, were log_2-tramsformed and then used to generate prognosis-risk score (PRS) for each patient. According to the algorithm we reported previously [15], a predictive principal component analysis (PCA) model was fitted in the validation cohort 1 using these four metabolites, and this PCA model was used to generate PRS for each patient in validation cohort 1 and to predict PRS for each case in validation cohort 2. The first principal component (a weighted average expression of four methionine cycle metabolites) was employed as PRS, as it accounted for the largest variability of the data. The 70th percentile of PRS values in validation cohort 1 was used as the cutoff value to divide patients from

validation cohort 1 and validation cohort 2 into two groups: those with low and high PRS.

Download and analysis of the public GEO microarray dataset

An GEO dataset, GSE23400, was downloaded from the NCBI Gene Expression Omnibus (http://www.ncbi.nlm.nih.gov/geo/). This dataset was normalized using the R function rma in the Bioconductor package affy and then $\log_2$-transformed.

Statistical analysis

Significant differences between two groups were computed using Wilcoxon's rank-sum test for data with skewed distribution or Student's t test for data with normal distribution. Survival analysis was carried out by means of Kaplan-Meier method followed with log-rank test. Multivariate Cox regression models were fitted using enter method to obtain hazard ratio values.

Statistical analysis was conducted with R software (R version 3.6.1, http://www.r-project.org). All statistical tests were 2-tailed, and p values less than 0.05 were considered significant.

References

1. X. Jin, L. Liu, J. Wu, X. Jin, G. Yu, L. Jia *et al.*, A multi-omics study delineates new molecular features and therapeutic targets for esophageal squamous cell carcinoma. *Clin Transl Med* **11**, e538 (2021).

2. M. B. Amin, F. L. Greene, S. B. Edge, C. C. Compton, J. E. Gershenwald, R. K. Brookland *et al.*, The Eighth Edition AJCC Cancer Staging Manual: Continuing to build a bridge from a population-based to a more "personalized" approach to cancer staging. *CA Cancer J Clin* **67**, 93-99 (2017).

3. V. E. Strong, T. A. D'Amico, L. Kleinberg, J. Ajani, Impact of the 7th Edition AJCC staging classification on the NCCN clinical practice guidelines in oncology for gastric and esophageal cancers. *J Natl Compr Canc Netw* **11**, 60-66 (2013).

4. J. A. Ajani, T. A. D'Amico, D. J. Bentrem, J. Chao, C. Corvera, P. Das *et al.*, Esophageal and Esophagogastric Junction Cancers, Version 2.2019, NCCN Clinical Practice Guidelines in Oncology. *J Natl Compr Canc Netw* **17**, 855-883 (2019).

5. J. A. Ajani, T. A. D'Amico, D. J. Bentrem, D. Cooke, C. Corvera, P. Das *et al.*, Esophageal and Esophagogastric Junction Cancers, Version 2.2023, NCCN Clinical Practice Guidelines in Oncology. *J Natl Compr Canc Netw* **21**, 393-422 (2023).

6. W. L. Chen, X. Jin, M. Wang, D. Liu, Q. Luo, H. Tian *et al.*, GLUT5-mediated fructose utilization drives lung cancer growth by stimulating fatty acid synthesis and AMPK/mTORC1 signaling. *JCI Insight* **5**, (2020).

7. L. Liu, J. Wu, M. Shi, F. Wang, H. Lu, J. Liu *et al.*, New Metabolic Alterations and A Predictive Marker Pipecolic Acid in Sera for Esophageal Squamous Cell Carcinoma. *Genomics Proteomics Bioinformatics* **20**, 670-687 (2022).

8. D. Liu, C. Dong, F. Wang, W. Liu, X. Jin, S. L. Qi *et al.*, Active post-transcriptional regulation and ACLY-mediated acetyl-CoA synthesis as a pivotal target of Shuang-Huang-Sheng-Bai formula for lung adenocarcinoma treatment. *Phytomedicine* **113**, 154732 (2023).

9. D. Liu, X. Jin, G. Yu, M. Wang, L. Liu, W. Zhang *et al.*, Oleanolic acid blocks the purine salvage pathway for cancer therapy by inactivating SOD1 and stimulating lysosomal proteolysis. *Mol Ther Oncolytics* **23**, 107-123 (2021).

10. P. Luo, D. Liu, Q. Zhang, F. Yang, Y. K. Wong, F. Xia *et al.*, Celastrol induces ferroptosis in activated HSCs to ameliorate hepatic fibrosis via targeting peroxiredoxins and HO-1. *Acta Pharm Sin B* **12**, 2300-2314 (2022).

11. F. Kou, B. Zhu, W. Zhou, C. Lv, Y. Cheng, H. Wei, Targeted metabolomics reveals dynamic portrayal of amino acids and derivatives in triple-negative breast cancer cells and culture media. *Mol Omics* **17**, 142-152 (2021).

12. Z. Gu, R. Eils, M. Schlesner, Complex heatmaps reveal patterns and correlations in multidimensional genomic data. *Bioinformatics* **32**, 2847-2849 (2016).

13. M. D. Robinson, D. J. McCarthy, G. K. Smyth, edgeR: a Bioconductor package for differential expression analysis of digital gene expression data. *Bioinformatics* **26**, 139-140 (2010).

14. A. A. Hakimi, E. Reznik, C. H. Lee, C. J. Creighton, A. R. Brannon, A. Luna *et al.*, An Integrated Metabolic Atlas of Clear Cell Renal Cell Carcinoma. *Cancer cell* **29**, 104-116 (2016).

15. W. L. Chen, J. H. Wang, A. H. Zhao, X. Xu, Y. H. Wang, T. L. Chen *et al.*, A distinct glucose metabolism signature of acute myeloid leukemia with prognostic value. *Blood* **124**, 1645-1654 (2014).

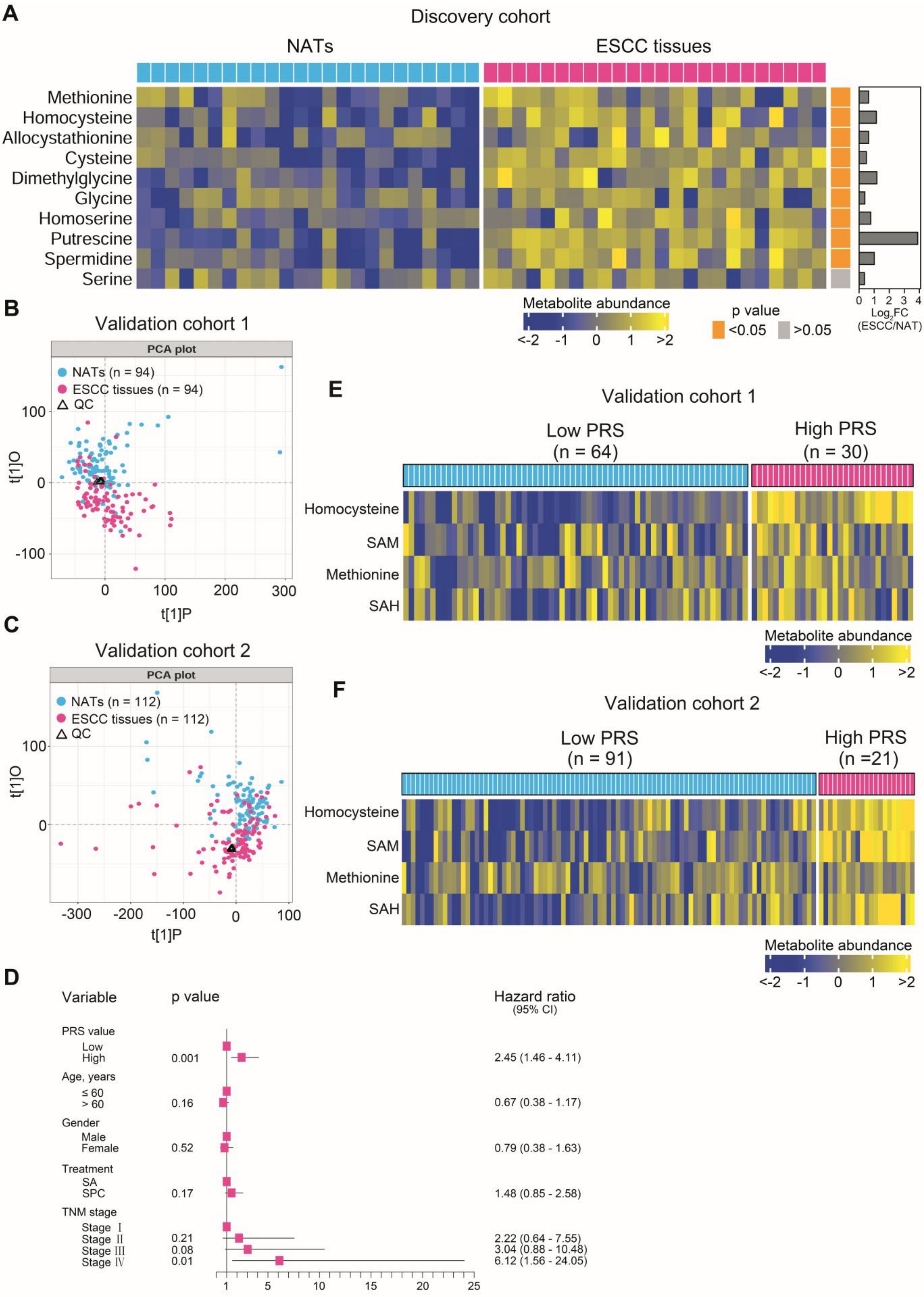
A
Discovery cohort
NATs
ESCC tissues
Methionine
Homocysteine
Allocystathionine
Cysteine
Dimethylglycine
Glycine
Homoserine
Putrescine
Spermidine
Serine
Metabolite abundance
<-2 -1 0 1 >2
p value
<0.05 >0.05
0 1 2 3 4
Log2FC (ESCC/NAT)
B
Validation cohort 1
PCA plot
NATs (n = 94)
ESCC tissues (n = 94)
QC
t[1]O
t[1]P
C
Validation cohort 2
PCA plot
NATs (n = 112)
ESCC tissues (n = 112)
QC
t[1]O
t[1]P
D
Variable
p value
Hazard ratio (95% CI)
PRS value
Low
High 0.001 2.45 (1.46 - 4.11)
Age, years
≤ 60
> 60 0.16 0.67 (0.38 - 1.17)
Gender
Male
Female 0.52 0.79 (0.38 - 1.63)
Treatment
SA
SPC 0.17 1.48 (0.85 - 2.58)
TNM stage
Stage Ⅰ
Stage Ⅱ 0.21 2.22 (0.64 - 7.55)
Stage Ⅲ 0.08 3.04 (0.88 - 10.48)
Stage Ⅳ 0.01 6.12 (1.56 - 24.05)
E
Validation cohort 1
Low PRS (n = 64)
High PRS (n = 30)
Homocysteine
SAM
Methionine
SAH
Metabolite abundance
F
Validation cohort 2
Low PRS (n = 91)
High PRS (n =21)
Homocysteine
SAM
Methionine
SAH
Metabolite abundance

Figure S1. Related to Figure 1. Activity and prognostic significance of the methionine cycle in clinical ESCC tissues of patients.

(A) Comparison of the metabolites involved in methionine metabolism between tumor tissues and matched NATs derived from the discovery cohort, which consisted of 24 treatment-naive patients with ESCC.

(B and C) PCA score plots of metabolomic data of validation cohort 1 (B) and validation cohort 2 (C). The quality control samples in both validation cohorts are clustered together with low dispersion, indicating low systemic variation during the measurement.

(D) Forest plot showing DFS hazard ratios of PRS and several well-known clinical parameters derived from a multivariate Cox regression model fitted using the combined validation cohort 1 and validation cohort 2.

(E and F) Heatmaps showing the intratumoral levels of four metabolites involved in the methionine cycle between patients with high and low PRS values in validation cohort 1 (E) and validation cohort 2 (F).

$^{**}p < 0.01$, $^{***}p < 0.001$ (Wilcoxon rank-sum test).

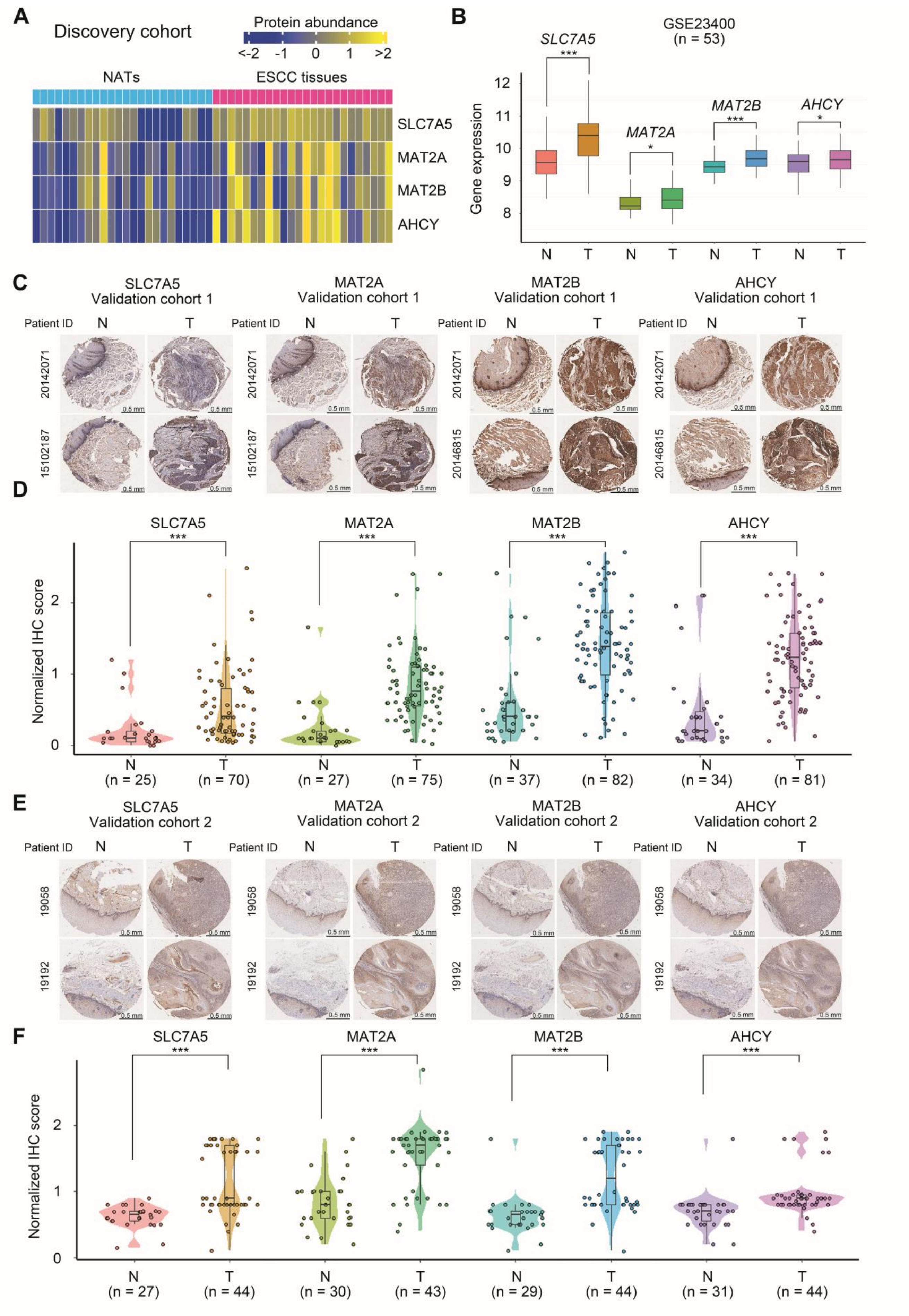

A
Discovery cohort
Protein abundance
<-2 -1 0 1 >2
NATs
ESCC tissues
SLC7A5
MAT2A
MAT2B
AHCY
B
GSE23400
(n = 53)
SLC7A5
MAT2A
MAT2B
AHCY
Gene expression
N T N T N T N T
C
SLC7A5 Validation cohort 1
MAT2A Validation cohort 1
MAT2B Validation cohort 1
AHCY Validation cohort 1
Patient ID
20142071
15102187
20146815
0.5 mm
D
Normalized IHC score
N (n = 25) T (n = 70) N (n = 27) T (n = 75) N (n = 37) T (n = 82) N (n = 34) T (n = 81)
E
SLC7A5 Validation cohort 2
MAT2A Validation cohort 2
MAT2B Validation cohort 2
AHCY Validation cohort 2
19058
19192
F
N (n = 27) T (n = 44) N (n = 30) T (n = 43) N (n = 29) T (n = 44) N (n = 31) T (n = 44)

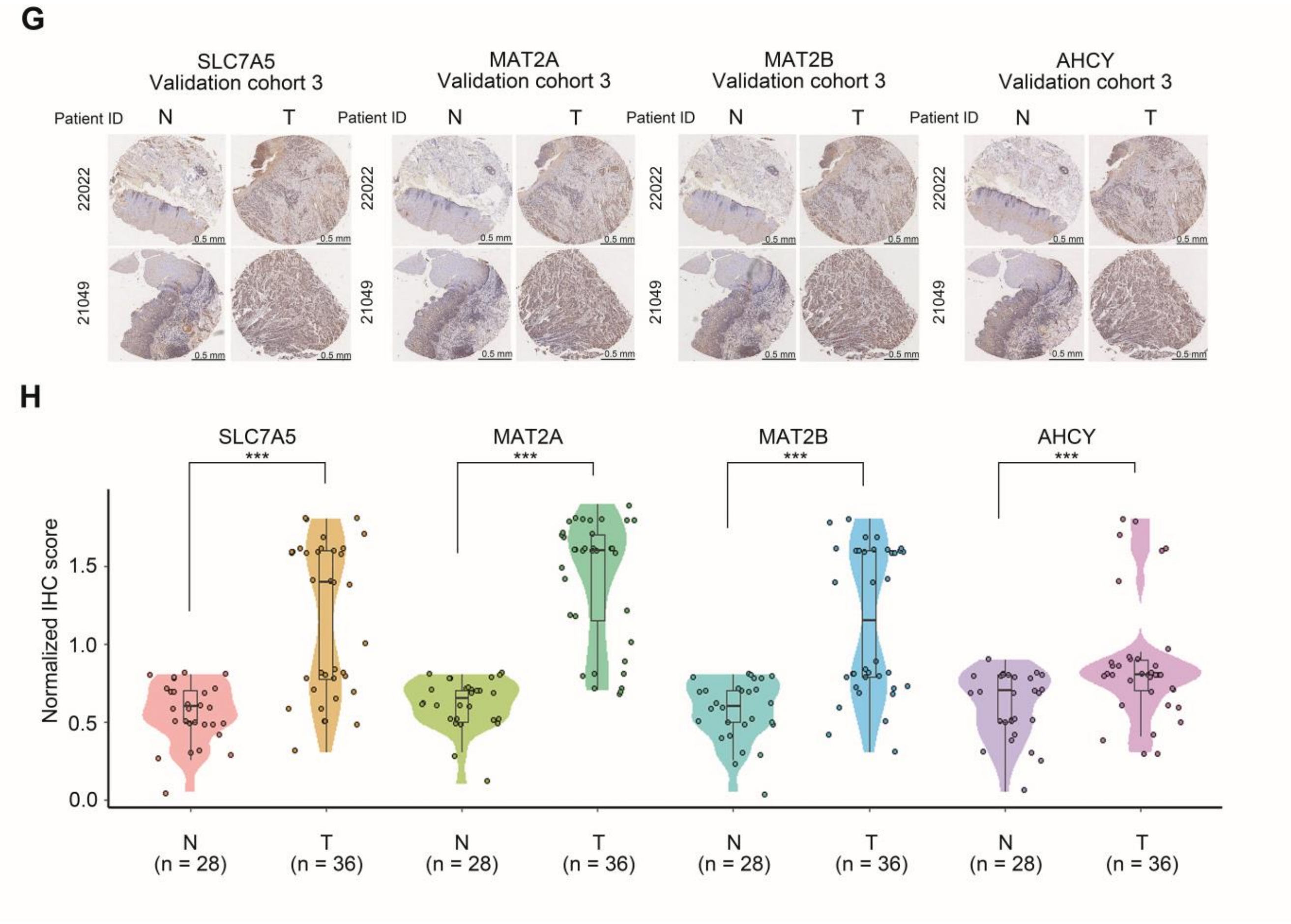

Figure S2. Related to Figure 1. Increased expression of methionine transporter and metabolic enzymes in clinical ESCC tissues.

(A) Proteomic profiling showing the protein abundance of SLC7A5, MAT2A, MAT2B, and AHCY between clinical ESCC tissues and matched NATs from the discovery cohort.

(B) Comparison of mRNA levels of *SLC7A5*, *MAT2A*, *MAT2B*, and *AHCY* between clinical ESCC tissues and matched NATs using the public gene expression dataset GSE23400.

(C-H) Protein levels of SLC7A5, MAT2A, MAT2B, and AHCY between clinical ESCC tissues and NATs derived from validation cohort 1 (C and D), validation cohort 2 (E and F) and validation cohort 3 (G and H), respectively. Representative IHC images (C, E and G) and statistical violin-box-scatter plots (D, F and H) were presented.

$^{*}p < 0.05$, $^{***}p < 0.001$ (Wilcoxon rank-sum test).

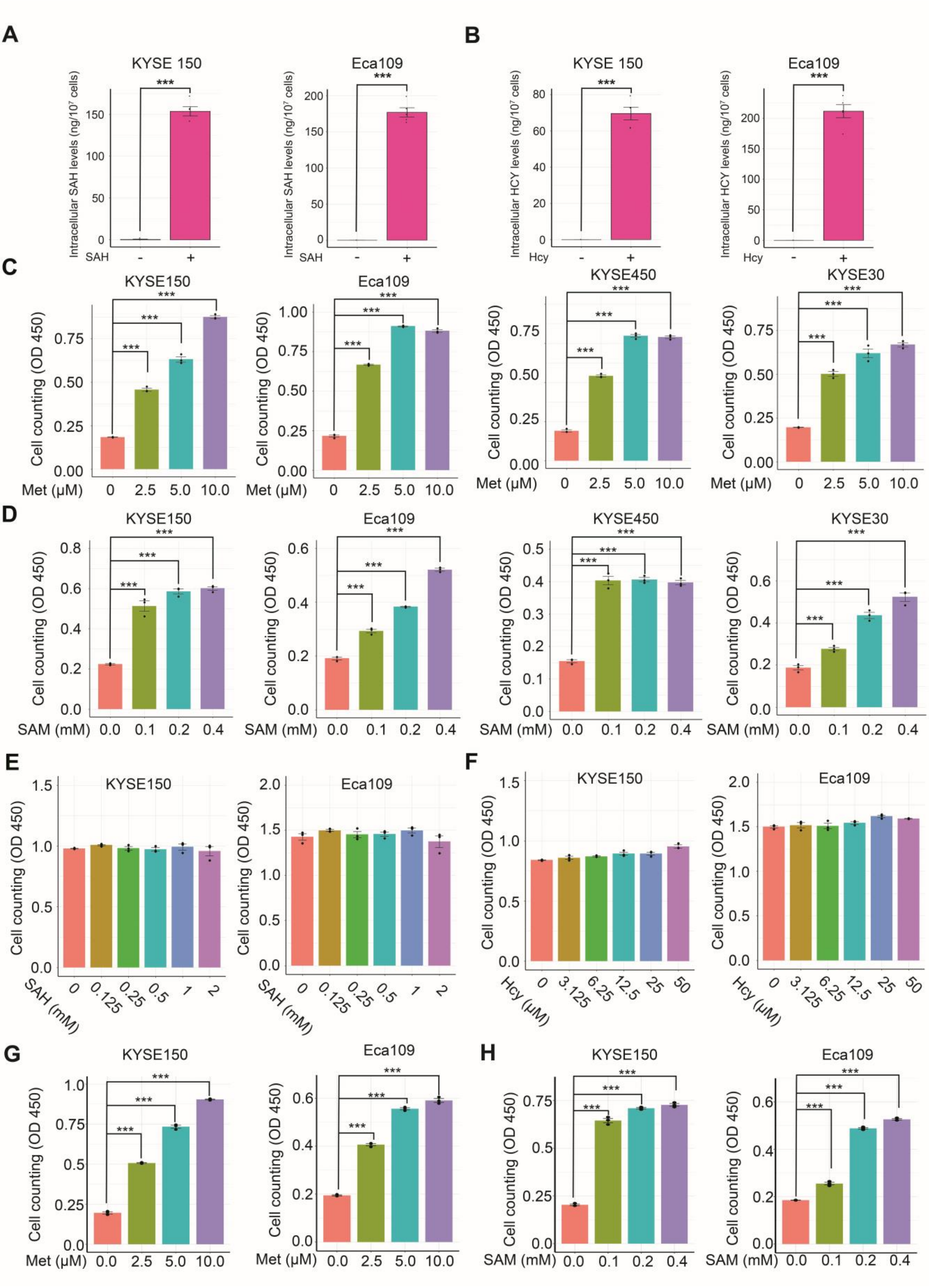
A
KYSE 150
Eca109
Intracellular SAH levels (ng/10⁷ cells)
SAH
-
+
***
B
KYSE 150
Eca109
Intracellular HCY levels (ng/10⁷ cells)
Hcy
C
KYSE150
Eca109
KYSE450
KYSE30
Cell counting (OD 450)
Met (μM)
0
2.5
5.0
10.0
D
KYSE150
Eca109
KYSE450
KYSE30
SAM (mM)
0.0
0.1
0.2
0.4
E
KYSE150
Eca109
SAH (mM)
0
0.125
0.25
0.5
1
2
F
KYSE150
Eca109
Hcy (μM)
0
3.125
6.25
12.5
25
50
G
KYSE150
Eca109
Met (μM)
0.0
2.5
5.0
10.0
H
KYSE150
Eca109
SAM (mM)
0.0
0.1
0.2
0.4

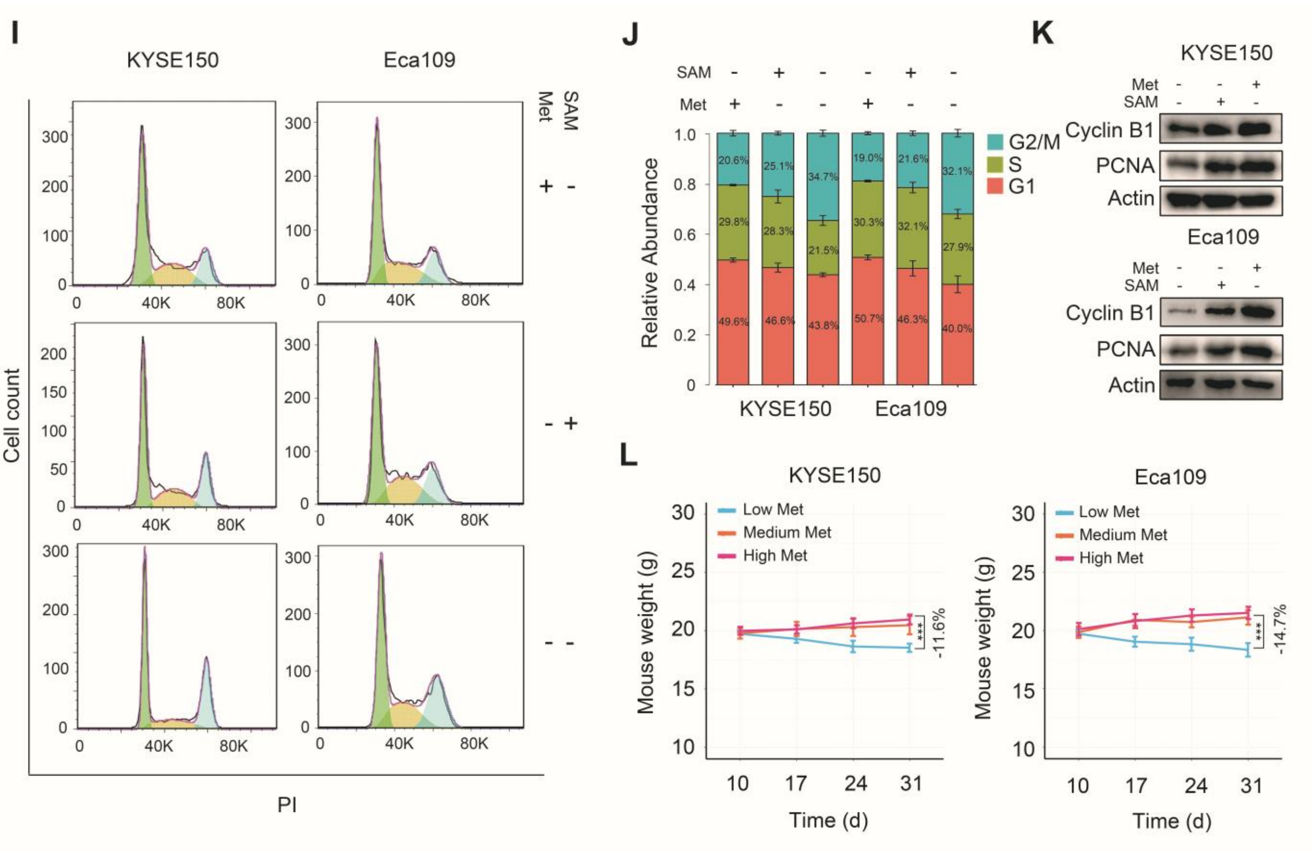

Figure S3. Related to Figure 2. Exogenous SAH and Hcy uptake by ESCC cells, influence of methionine and its downstream metabolites on ESCC cell growth and cell cycle progression, and impact of dietary methionine on the body weight of mice.

(A and B) Comparison of intracellular SAH (C) and Hcy (D) levels between ESCC cells cultured in medium with 0.5 mM SAH or 50 µM Hcy and methionine/SAH/Hcy-free medium for 24 hours.

(C-F) Methionine and SAM (C and D), but not SAH and Hcy (E and F), promoted ESCC cell proliferation in a dose-dependent manner *in vitro*.

(G and H) Methionine (G) and SAM (H) boosted ESCC cell propagation in a dose-dependent manner in the dialyzed medium *in vitro*.

(I) Influence of methionine and SAM on cell cycle of ESCC cells.

(J) Quantification of the percentage values of ESCC cells at different cell cycle phases from (I).

(K) Western blot assays showing the influence of methionine and SAM on the expression of a G2/M progression marker, cyclin B1, and a cell proliferation marker PCNA.

(L) Impact of different levels of dietary methionine on the body weight of mice harboring subcutaneous KYSE150 or Eca109 tumors.

Error bars represent mean ± SEM. $^{*}p < 0.05$, $^{**}p < 0.01$, $^{***}p < 0.001$ (Student's t test).

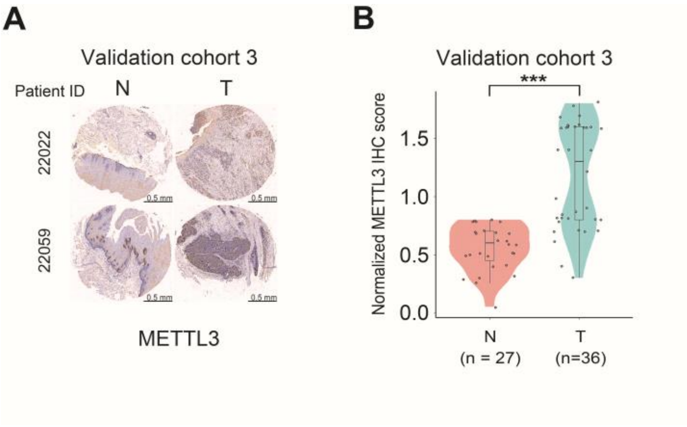

Figure S4. Related to Figure 3. Comparison of METTL3 expression between ESCC tissues and NATs from validation cohort 3.

(A) Representative IHC images.

(B) Statistical violin-box-scatter plots.

***$p < 0.001$ (Wilcoxon rank-sum test).

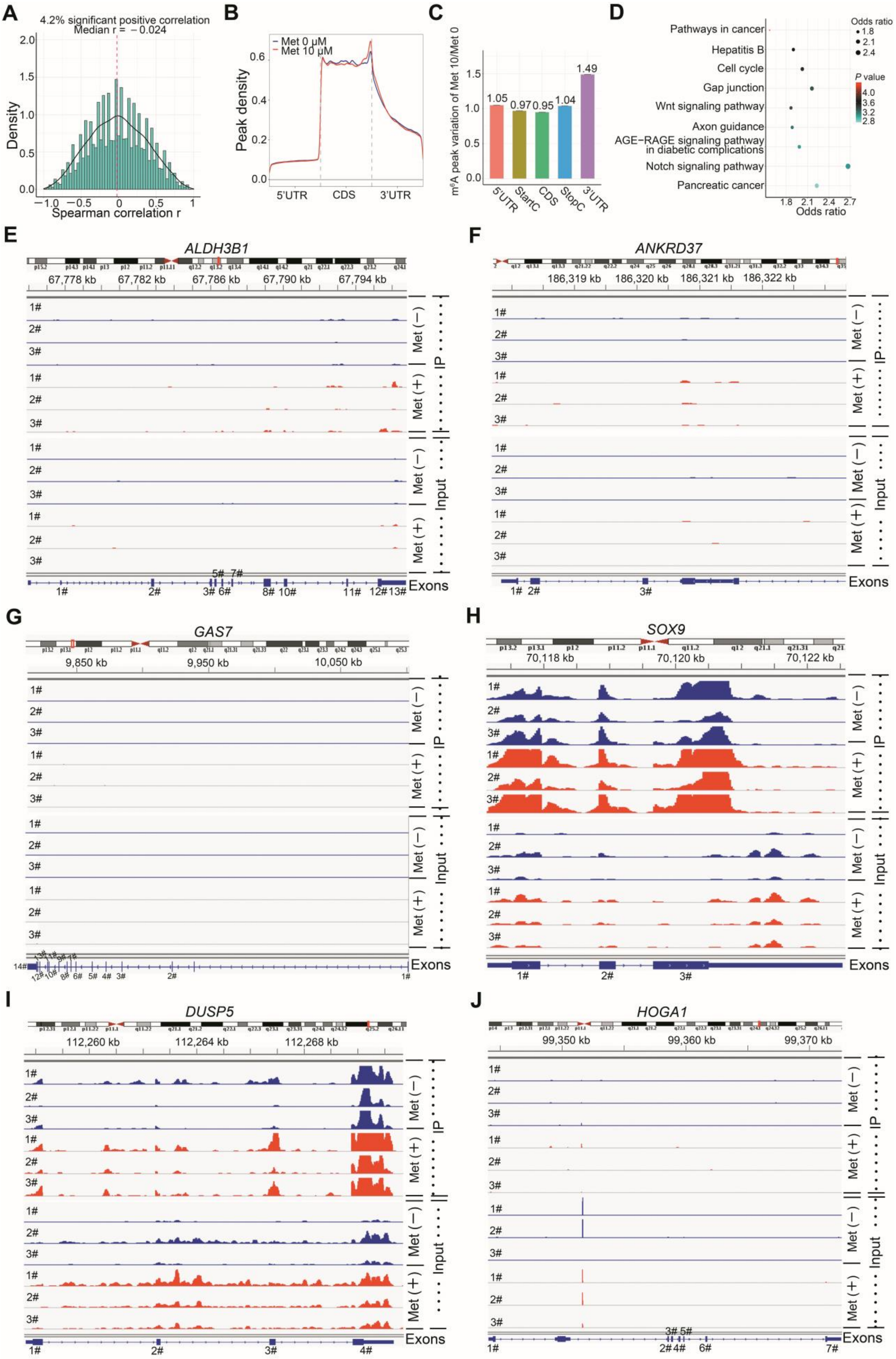

A
4.2% significant positive correlation
Median r = − 0.024
Density
Spearman correlation r
B
Met 0 μM
Met 10 μM
Peak density
5'UTR
CDS
3'UTR
C
m6A peak variation of Met 10/Met 0
1.05
0.97
0.95
1.04
1.49
5'UTR
StartC
CDS
StopC
3'UTR
D
Pathways in cancer
Hepatitis B
Cell cycle
Gap junction
Wnt signaling pathway
Axon guidance
AGE−RAGE signaling pathway in diabetic complications
Notch signaling pathway
Pancreatic cancer
Odds ratio
P value
E
ALDH3B1
F
ANKRD37
G
GAS7
H
SOX9
I
DUSP5
J
HOGA1
Met (−)
Met (+)
IP
Input
Exons

Figure S5. Related to Figure 4. Correlation analysis between the gene expression levels in the two different experimental settings, and the influence of methionine on mRNA m^{6}A methylation of KYSE150 cells.

(A) Density plot of Spearman correlation coefficient r values between genes in methionine-treated KYSE150 cells *in vitro* and esophageal tissues from 4-NQO-induced mice with methionine administration. Notably, only 4.2% of genes exhibit a significant positive correlation, and the median correlation coefficient r value registers at a relatively low -0.24.

(B) Distribution of m^{6}A peaks at different mRNA regions between KYSE150 cells cultured in medium with 10 μM methionine and methionine-free medium.

(C) Bar plots displaying the ratios of percentage values of m^{6}A peaks at different mRNA regions in KYSE150 cells. The ratio value at a specific mRNA region was calculated by dividing the percentage of m^{6}A peak number of KYSE150 cells treated with methionine by the percentage of m^{6}A peak number of KYSE150 cells treated without methionine.

(D) KEGG pathway enrichment analysis of gene transcripts with significantly perturbed m^{6}A modification.

(E-J) Analysis of m^{6}A peaks in *ALDH3B1*, *ANKRD37*, *GAS7*, *SOX9*, *DUSP5*, and *HOGA1* mRNAs between KYSE150 cells cultured in medium with 10 μM methionine and methionine-free medium.

UTR, untranslated regions; CDS, coding sequence.

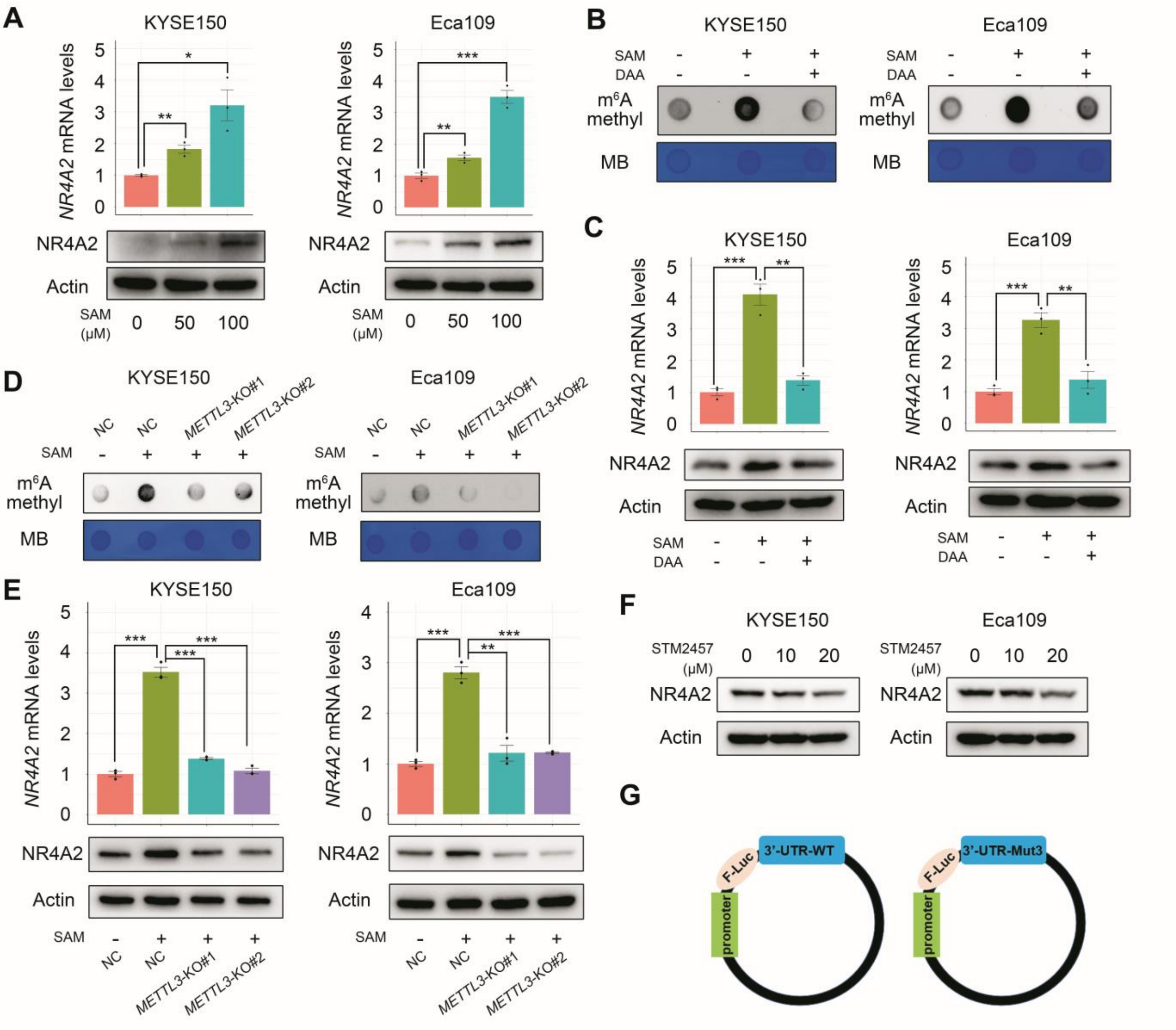

Figure S6. Related to Figure 5. SAM eliciting NR4A2 expression in ESCC cells via METTL3-mediated RNA m^{6}A methylation, and construction of the fusion expression of wild-type or mutant 3'-UTR fragment of *NR4A2* with firefly luciferase reporter.

(A) SAM eliciting NR4A2 expression at both mRNA and protein levels in KYSE150 and Eca109 cells. Cells were cultured in methionine-free medium for 24 hours.

(B and C) Treatment with the methylation inhibitor DAA at 10 μM for 24 hours influencing SAM-induced RNA m^{6}A methylation (B) and NR4A2 expression at both mRNA and protein levels (C) in ESCC cells. Cells were cultured in methionine-free medium.

(D) Impact of *METTL3* abrogation on SAM-induced RNA m^{6}A methylation in ESCC cells. Cells

were cultured in methionine-free medium.

(E) Negative influence of SAM-induced NR4A2 expression at both mRNA and protein levels in ESCC cells by *METTL3* ablation. Cells were cultured in methionine-free medium.

(F) Influence of STM2457 treatment on NR4A2 expression in ESCC cells. Cells were cultured in complete medium.

(G) Schematic diagram showing the fusion of wild-type or mutant 3'-UTR fragment of *NR4A2* with firefly luciferase reporter. The mutant 3'-UTR fragment contains an A-T substitution in m^6A consensus sequence as depicted in Figure 5K.

Error bars represent mean ± SEM. $^{*}p < 0.05$, $^{**}p < 0.01$, $^{***}p < 0.001$ (Student's t test).

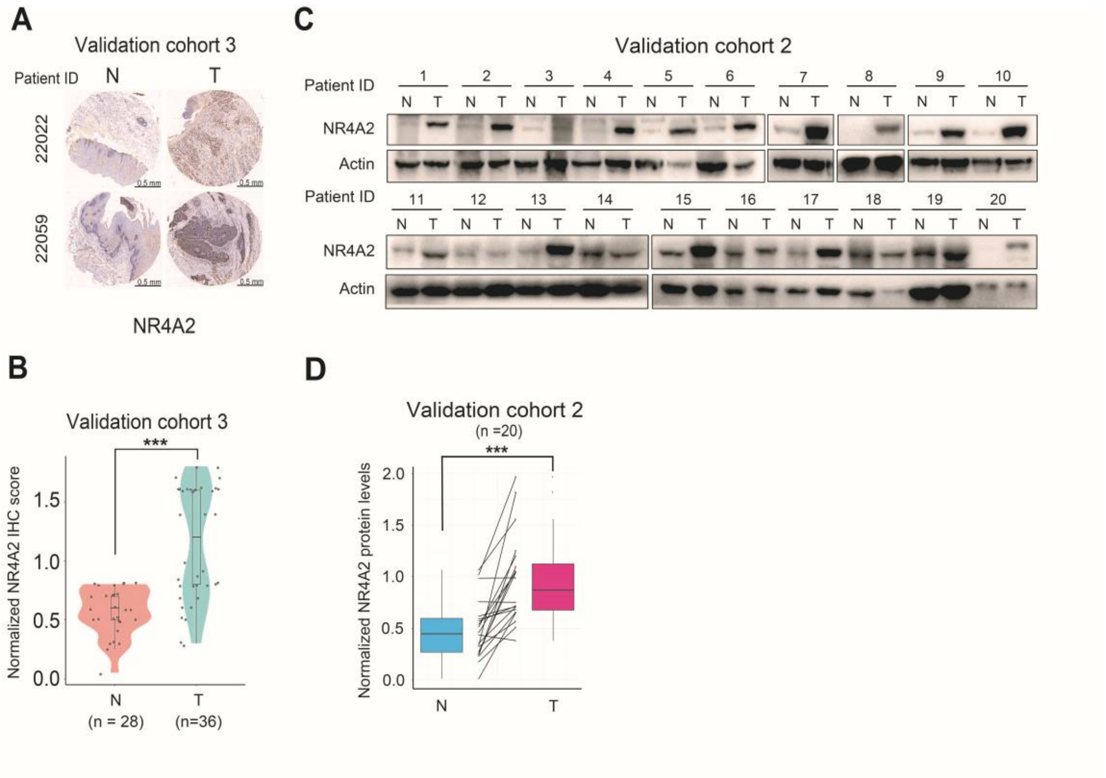

Figure S7. Related to Figure 7. Increased expression of NR4A2 in clinical ESCC tissues.

(A and B) IHC staining analysis comparing NR4A2 protein levels between clinical ESCC tissues and NATs derived from validation cohort 3. Representative IHC images (A) and statistical violin-box-scatter plots (B) are shown.

(C and D) Western blot assay comparing NR4A2 protein abundance between clinical ESCC tissues and paired NATs derived from validation cohort 2. Statistical analysis is shown in (D).

$^{***}p < 0.001$ (Wilcoxon rank-sum test).

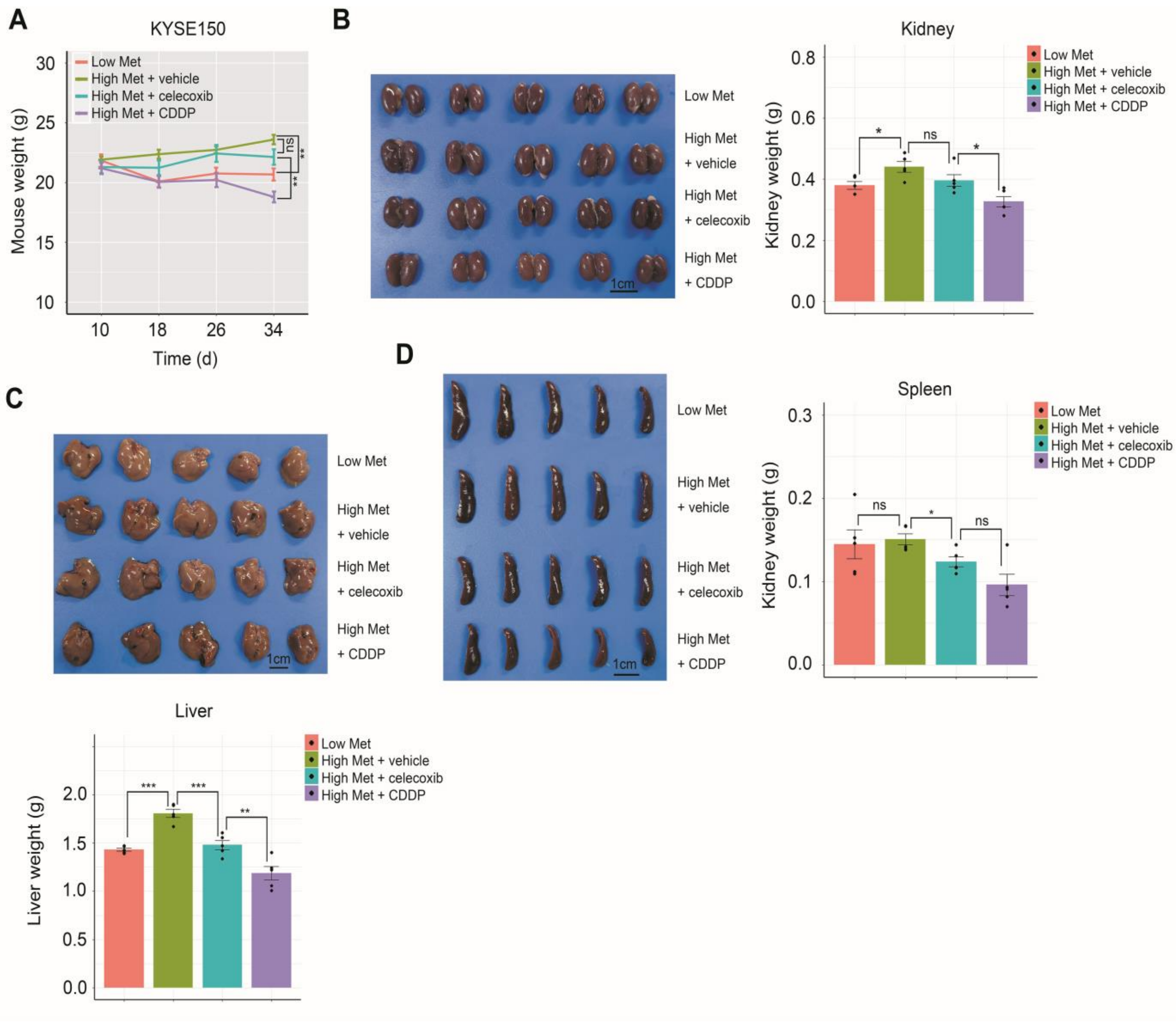

Figure S8. Related to Figure 8. Side effect analysis of oral celecoxib administration in mice harboring KYSE150 xenograft tumors.

(A) Comparison of the mouse weight over time among four treatment groups.

(B) Comparison of the kidney weight among four treatment groups.

(C) Comparison of the liver weight among four treatment groups.

(D) Comparison of the spleen weight among four treatment groups.

Ns, no significance.

Error bars represent mean ± SEM. $^{*}p < 0.05$, $^{**}p < 0.01$, $^{***}p < 0.001$ (Student's t test).

Table S1. Basic characteristics of the discovery cohort, related to Figure 1.

Characteristic	ESCC (n = 24)
Age, years	
Median (range)	68 (50-76)
≥ 60 years, no. (%)	21 (87.5)
< 60 years, no. (%)	3 (12.5)
Sex, no. (%)	
Male	15 (62.5)
Female	9 (37.5)
Alcohol consumption (habitual), no. (%)	
Never	22 (91.7)
Previous	2 (8.3)
Smoking status, no. (%)	
Never smoker	20 (83.3)
Ex-smoker	4 (16.7)
Hypertension, no. (%)	
Yes	5 (20.8)
No	19 (79.2)
Diabetes, no. (%)	
Yes	3 (12.5)
No	21 (87.5)
Histological type, no. (%)	
Squamous-cell carcinoma	24 (100)
Other	0
Treatment protocol, no. (%)	
Surgery alone	19 (79.2)
Surgery plus adjuvant chemotherapy	5 (20.8)
Clinical T stage, no. (%)	
T1	1 (4.2)
T2	3 (12.5)
T3	20 (83.3)
Clinical N stage, no. (%)	
N0	14 (58.3)
N1	8 (33.3)
N2	0
N3	2 (8.3)
Clinical stage group, no. (%)	
I	1 (4.2)
II	14 (58.3)
III	7 (29.2)
IV	2 (8.3)

Table S2. Basic characteristics of the validation cohort 1, related to Figure 1.

Characteristic	ESCC (n = 94)
Age, years	
Median (range)	64 (49-79)
≥ 60 years, no. (%)	70 (74.5)
< 60 years, no. (%)	24 (25.5)
Sex, no. (%)	
Male	71 (75.5)
Female	23 (24.5)
Alcohol consumption (habitual), no. (%)	
Never	56 (59.6)
Previous	38 (40.4)
Smoking status, no. (%)	
Never smoker	53 (56.4)
Ex-smoker	41 (43.6)
Hypertension, no. (%)	
Yes	18 (19.1)
No	76 (80.9)
Diabetes, no. (%)	
Yes	4 (4.3)
No	90 (95.7)
Histological type, no. (%)	
Squamous-cell carcinoma	94 (100)
Other	0
Treatment protocol, no. (%)	
Surgery alone	68 (72.3)
Surgery plus adjuvant chemotherapy	26 (27.7)
Clinical T stage, no. (%)	
T1	7 (7.4)
T2	24 (25.5)
T3	63 (67.0)
Clinical N stage, no. (%)	
N0	53 (56.4)
N1	26 (27.7)
N2	12 (12.8)
N3	3 (3.2)
Clinical stage group, no. (%)	
I	4 (4.3)
II	52 (55.3)
III	35 (37.2)
IV	3 (3.2)

Table S3. Basic characteristics of the validation cohort 2, related to Figure 1.

Characteristic	ESCC (n = 112)
Age, years	
Median (range)	68 (53-80)
≥60 years, no. (%)	93 (83.0)
<60 years, no. (%)	19 (17.0)
Sex, no. (%)	
Male	81 (72.3)
Female	31 (27.7)
Alcohol consumption (habitual), no. (%)	
Never	98 (87.5)
Previous	14 (12.5)
Smoking status, no. (%)	
Never smoker	76 (67.9)
Ex-smoker	36 (32.1)
Hypertension, no. (%)	
Yes	32 (28.6)
No	80 (71.4)
Diabetes, no. (%)	
Yes	13 (11.6)
No	99 (88.4)
Histological type, no. (%)	
Squamous-cell carcinoma	112 (100)
Other	0
Treatment protocol, no. (%)	
Surgery alone	73 (65.2)
Neoadjuvant chemotherapy	3 (2.7)
Surgery plus adjuvant chemotherapy	35 (31.3)
Chemotherapy only	1 (0.8)
Clinical T stage, no. (%)	
T1	16 (14.3)
T2	22 (19.6)
T3	61 (54.5)
T4	11 (9.8)
Unknow	2 (1.8)
Clinical N stage, no. (%)	
N0	58 (51.8)
N1	31 (27.7)
N2	16 (14.3)
N3	5 (4.5)
Unknow	2 (1.8)
Clinical stage group, no. (%)	
I	18 (16.1)
II	33 (29.5)

Characteristic	ESCC (n = 112)
III	46 (41.1)
IV	13 (11.6)
Unknow	2 (1.8)

Table S4. Basic characteristics of the validation cohort 3, related to Figure S2.

Characteristic	ESCC (n = 41)
Age, years	
Median (range)	66 (46-78)
≥ 60 years, no. (%)	35 (85.4)
< 60 years, no. (%)	6 (14.6)
Sex, no. (%)	
Male	27 (65.9)
Female	14 (34.1)
Alcohol consumption (habitual), no. (%)	
Never	37 (90.2)
Previous	4 (9.8)
Smoking status, no. (%)	
Never smoker	32 (78.0)
Ex-smoker	9 (22.0)
Hypertension, no. (%)	
Yes	13 (31.7)
No	28 (68.3)
Diabetes, no. (%)	
Yes	2 (4.9)
No	39 (95.1)
Histological type, no. (%)	
Squamous-cell carcinoma	41 (100)
Other	0
Treatment protocol, no. (%)	
Surgery alone	38 (92.7)
Surgery plus adjuvant chemotherapy	3 (7.3)
Clinical T stage, no. (%)	
T1	1 (2.4)
T2	10 (24.4)
T3	27 (65.9)
T4	3 (7.3)
Clinical N stage, no. (%)	
N0	16 (39.0)
N1	13 (31.7)
N2	10 (24.4)
N3	2 (4.9)
Clinical stage group, no. (%)	
I	3 (7.3)
II	25 (61.0)
III	13 (31.7)

Table S5. The ingredients of high methionine (0.86%), medium methionine (0.35%), and low methionine (0.17%) diets for mouse, related to Figure 2.

Mouse diet with 0.86% methionine

Ingredient	gram	kcal
Arginine	11.2	45
Glutamic Acid	27	108
Glycine	23.3	93
Histidine-HCl-H2O	3.3	13
Isoleucine	8.2	33
Leucine	11.1	44
Lysine-HCl	18	72
Methionine	8.6	34
Phenylalanine	11.6	46
Threonine	8.2	33
Tryptophan	1.8	7
Valine	8.2	33
Maltodextrin	50	200
Cornstarch	432.5	1730
Dextrose	200	800
Corn Oil	80	720
Cellulose	50	0
Mineral Mix#200000	35	0
Vitamin Mix#300050	10	40
Choline Bitartrate	2	0
Yellow Dye	0.05	0

Mouse diet with 0.35% methionine

Ingredient	gram	kcal
Arginine	11.2	45
Glutamic Acid	32.1	128
Glycine	23.3	93
Histidine-HCl-H2O	3.3	13
Isoleucine	8.2	33
Leucine	11.1	44
Lysine-HCl	18	72
Methionine	3.5	7
Phenylalanine	11.6	46
Threonine	8.2	33
Tryptophan	1.8	7
Valine	8.2	33
Maltodextrin	50	200

Ingredient	gram	kcal
Cornstarch	432.5	1730
Dextrose	200	800
Corn Oil	80	720
Cellulose	50	0
Mineral Mix#200000	35	0
Vitamin Mix#300050	10	40
Choline Bitartrate	2	0
Yellow Dye	0.05	0

Mouse diet with 0.17% methionine

Ingredient	gram	kcal
Arginine	11.2	45
Glutamic Acid	33.88	136
Glycine	23.3	93
Histidine-HCl-H2O	3.3	13
Isoleucine	8.2	33
Leucine	11.1	44
Lysine-HCl	18	72
Methionine	1.72	7
Phenylalanine	11.6	46
Threonine	8.2	33
Tryptophan	1.8	7
Valine	8.2	33
Maltodextrin	50	200
Cornstarch	432.5	1730
Dextrose	200	800
Corn Oil	80	720
Cellulose	50	0
Mineral Mix#200000	35	0
Vitamin Mix#300050	10	40
Choline Bitartrate	2	0
Yellow Dye	0.05	0
