# Supplementary figures and images for: Unveiling the methionine cycle: a key metabolic signature and NR4A2 as a methionine-responsive oncogene in esophageal squamous cell carcinoma
Source: Cell Death Differ. 2024 Apr 3;31(5):558–73. doi: 10.1038/s41418-024-01285-7 (PMC11094133; doi:10.1038/s41418-024-01285-7)

2F

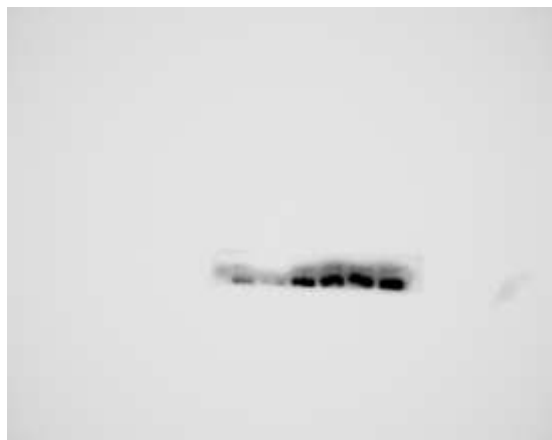

2G

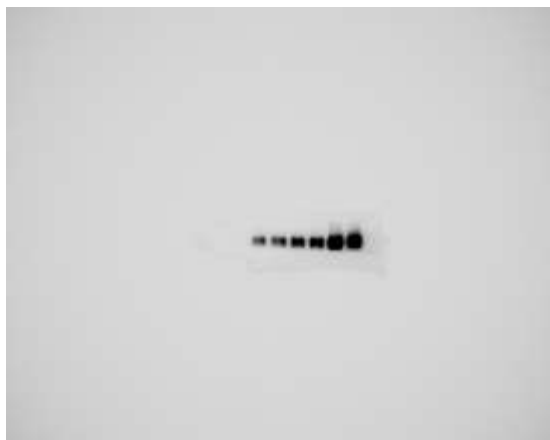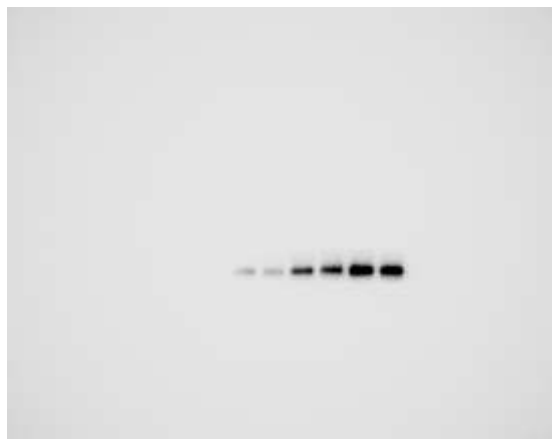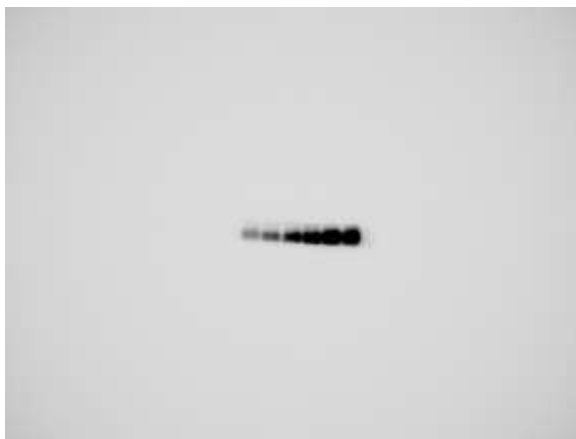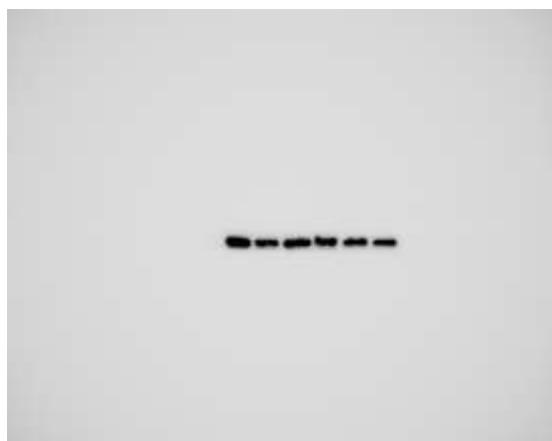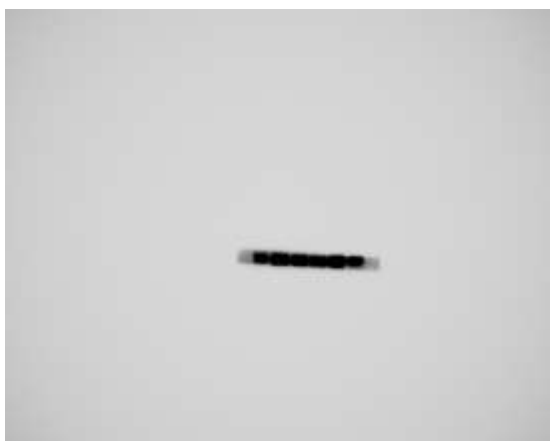

3G

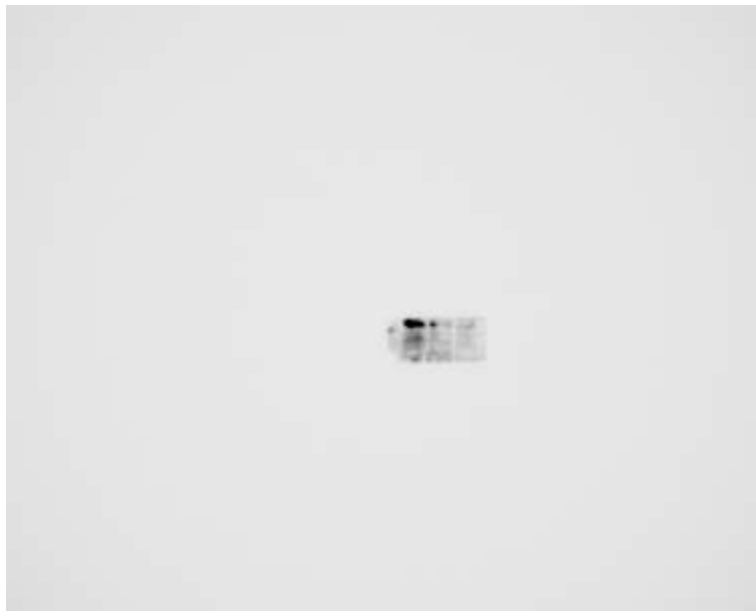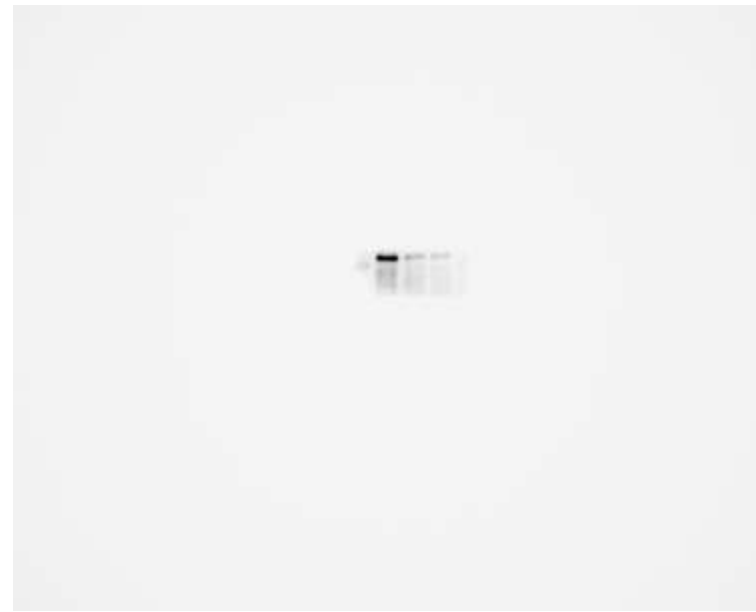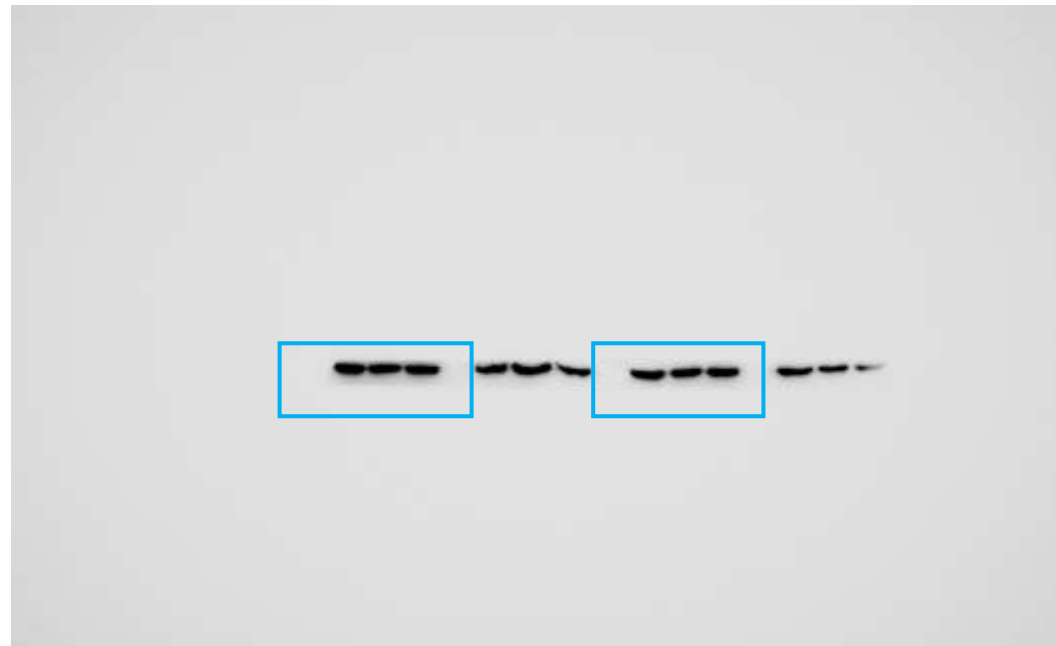

5A

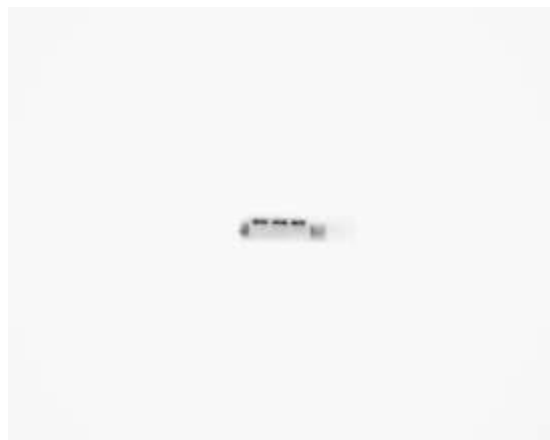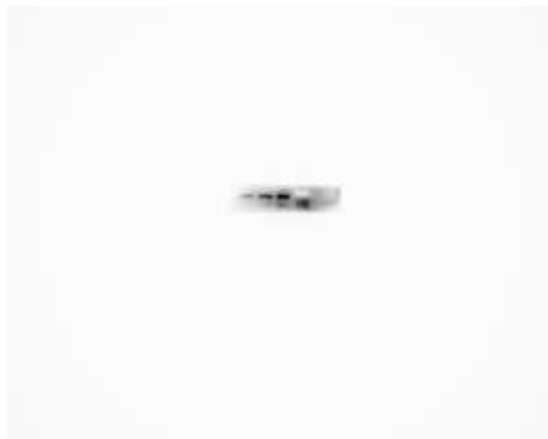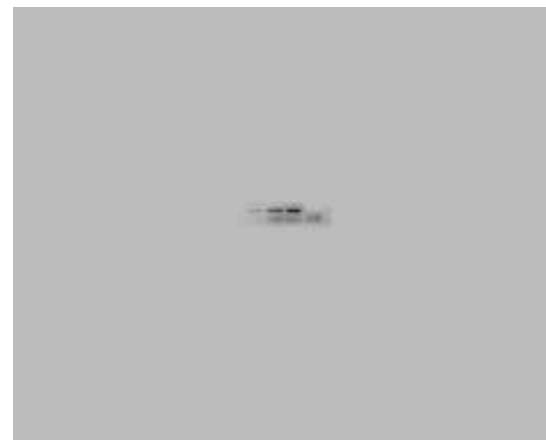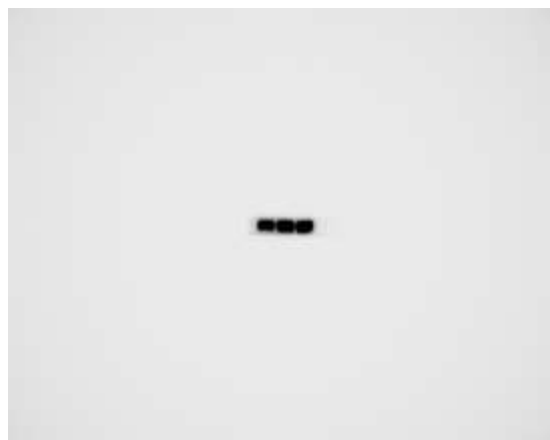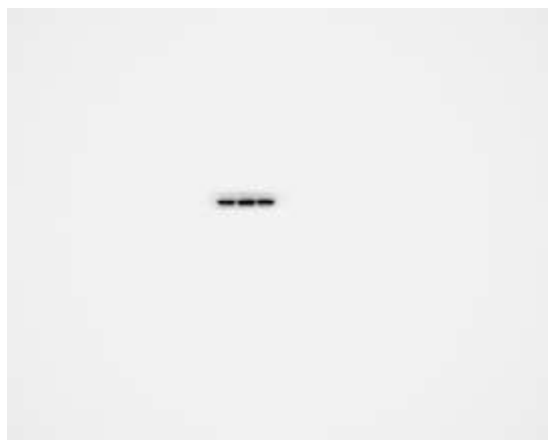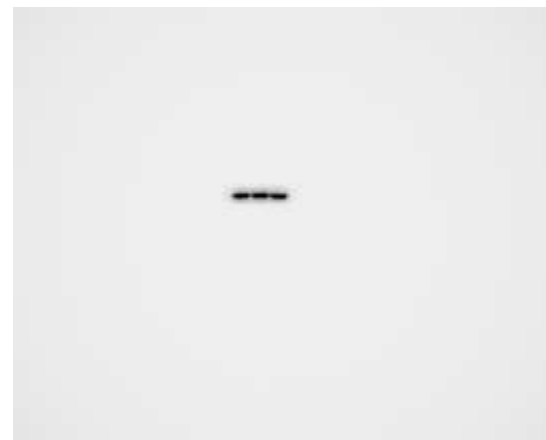

5D

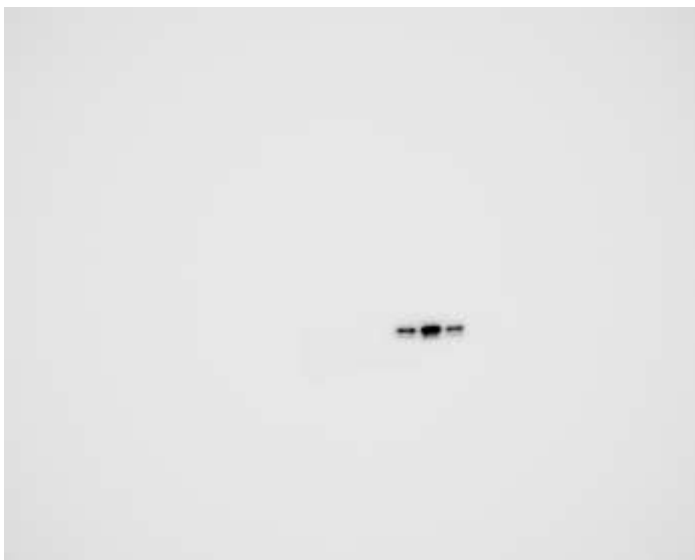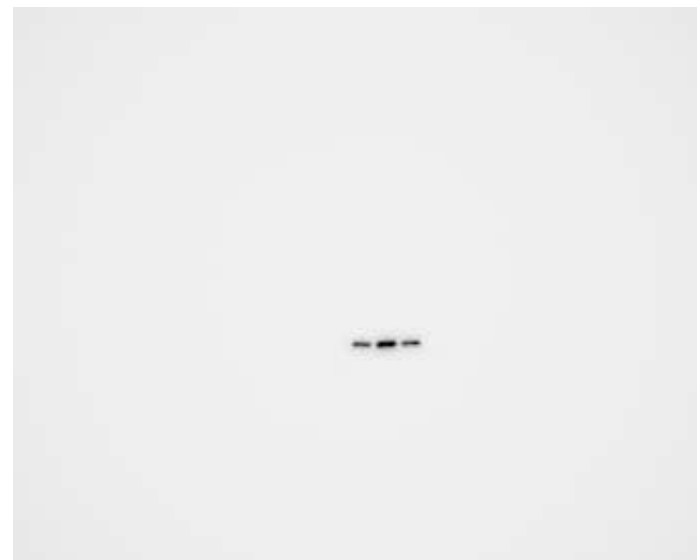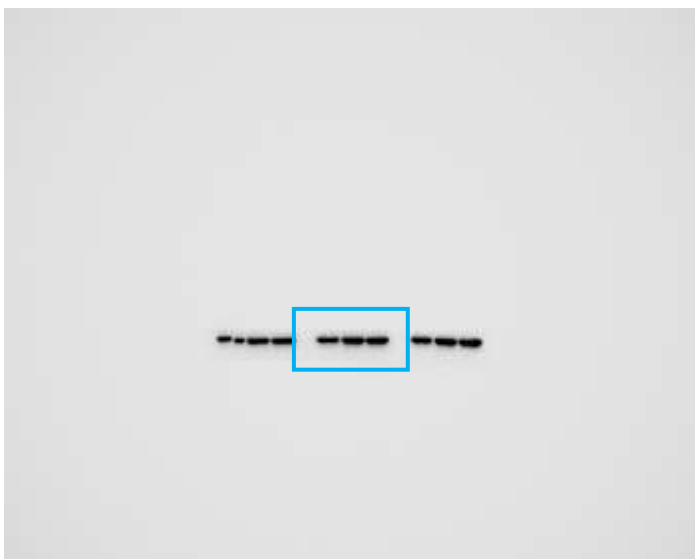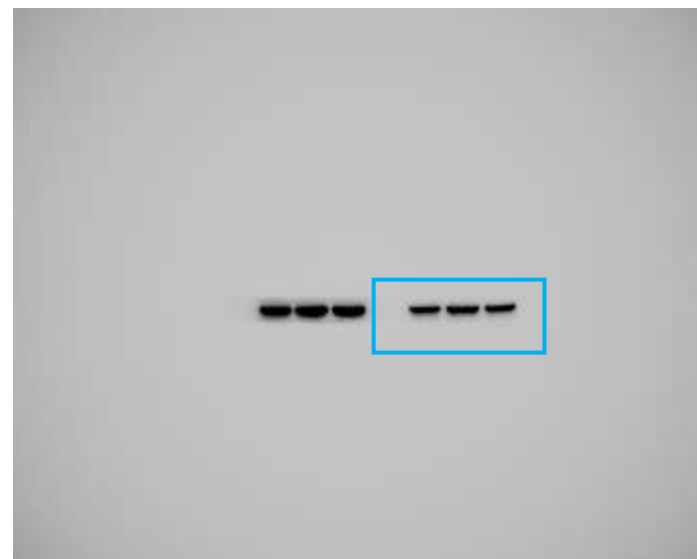

5E

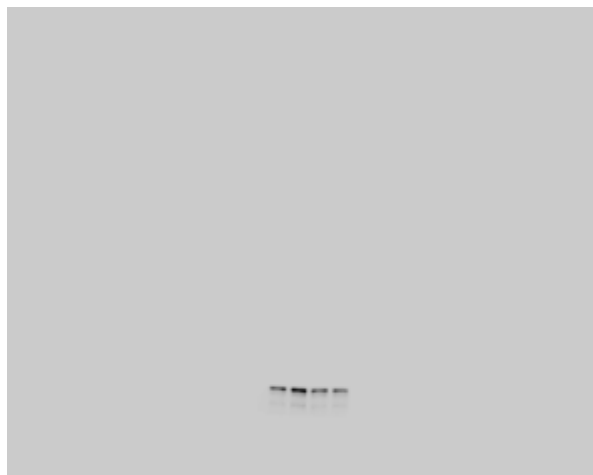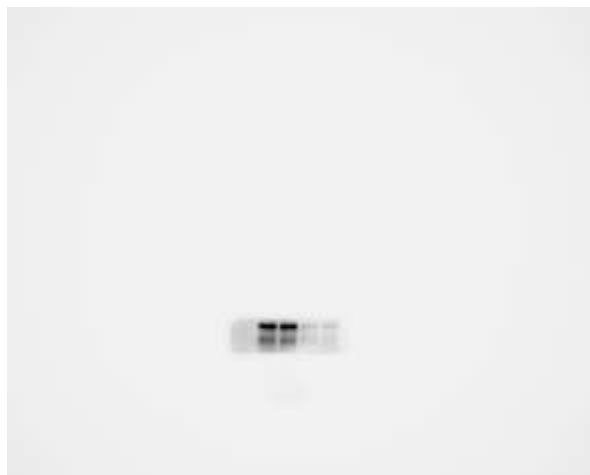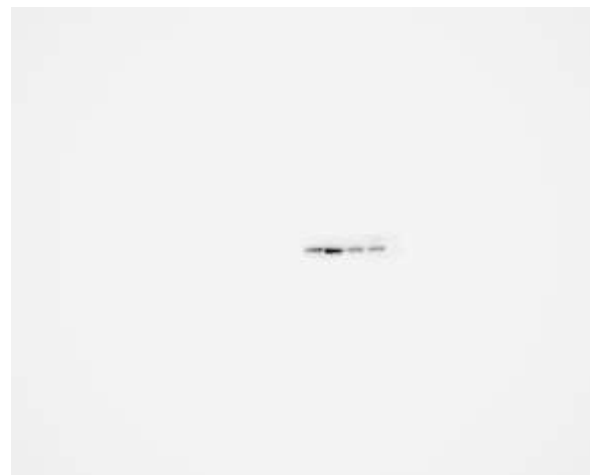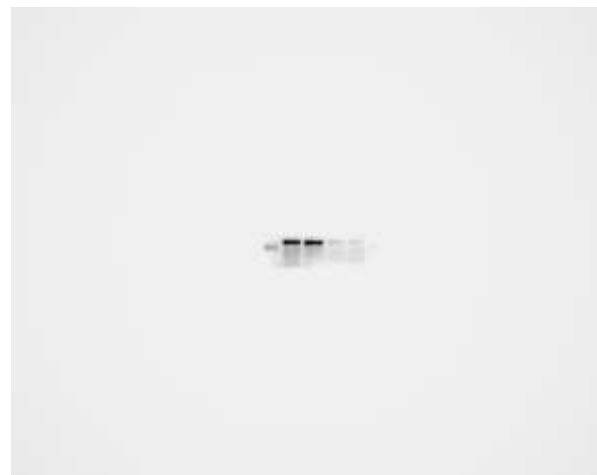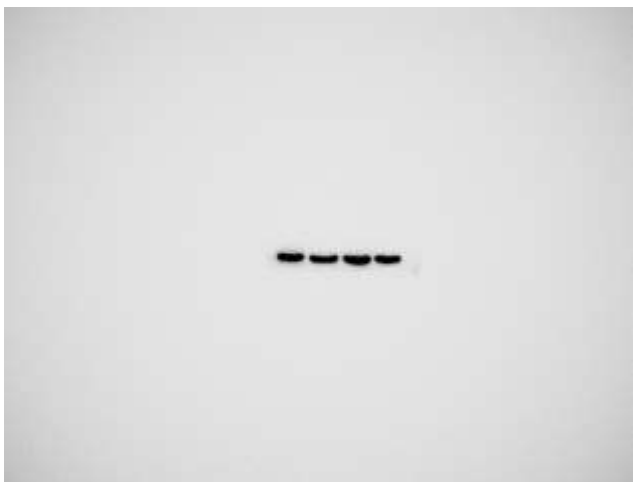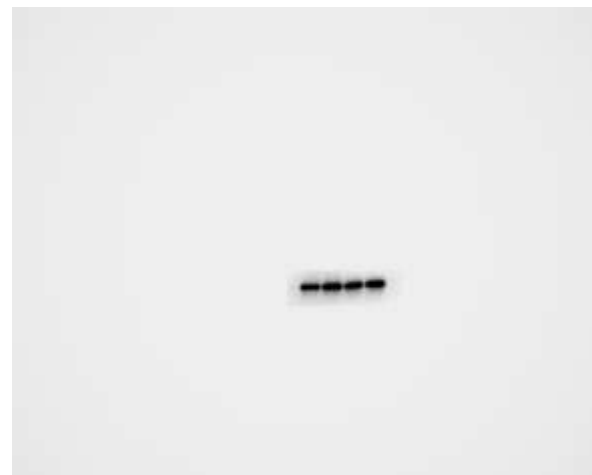

5J

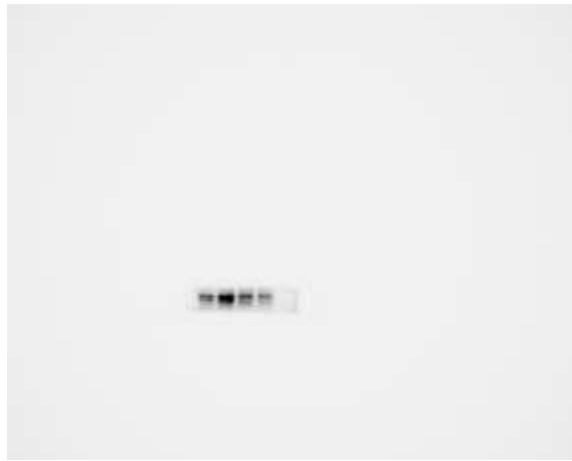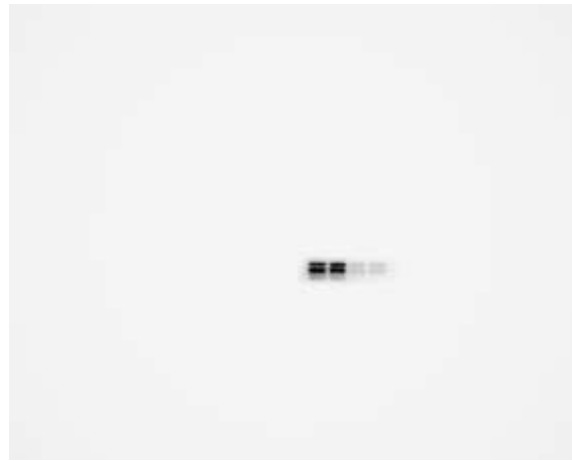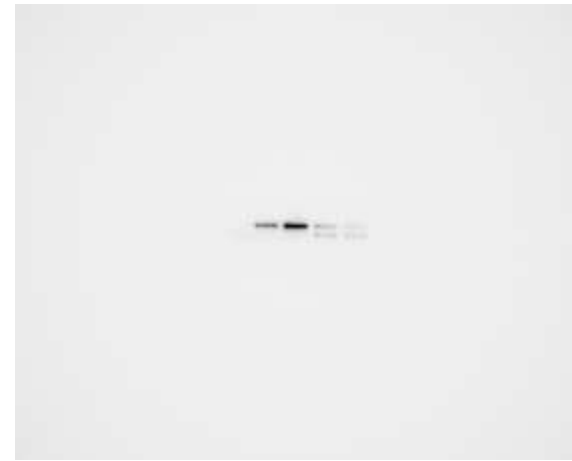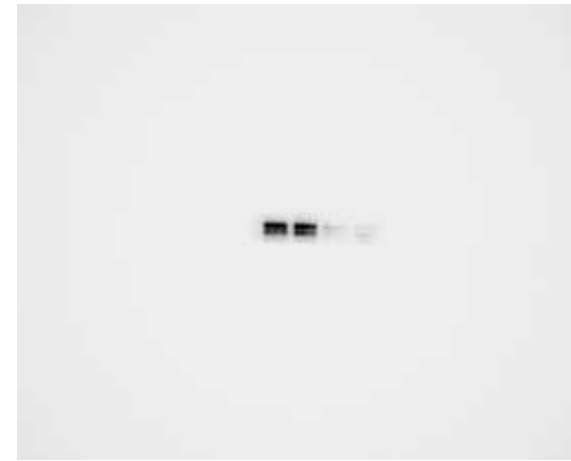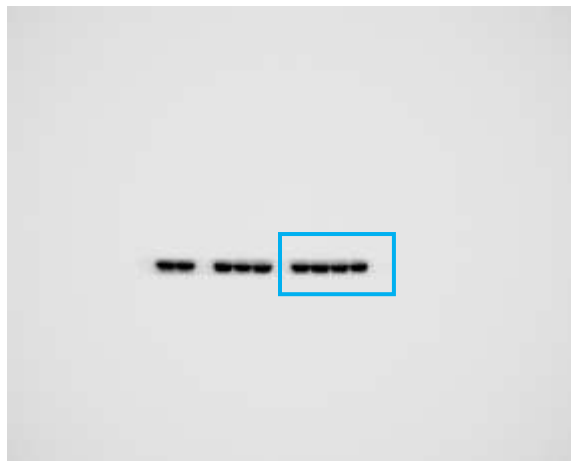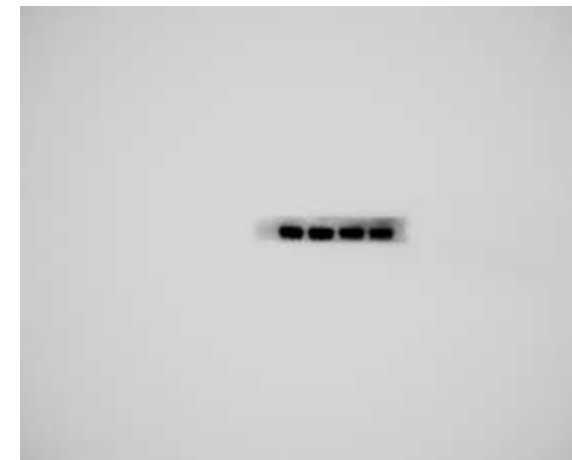

6A

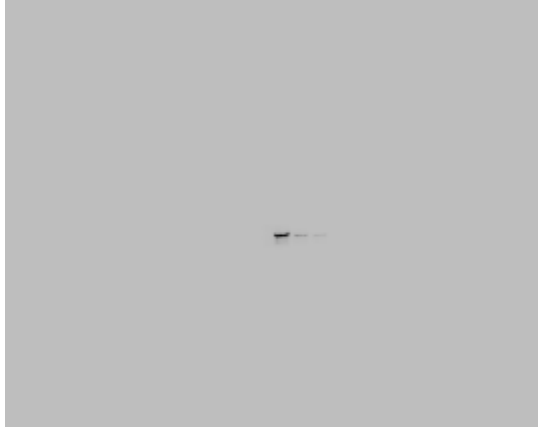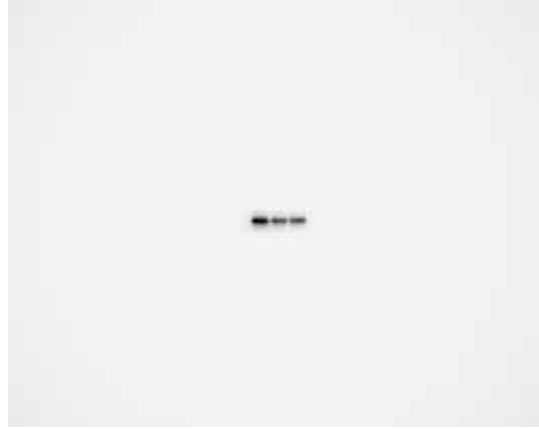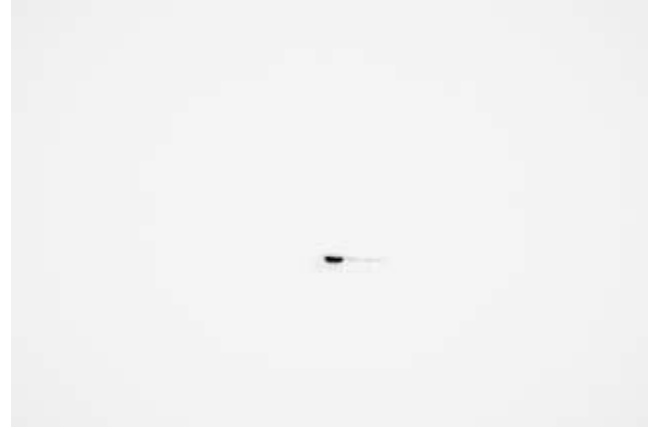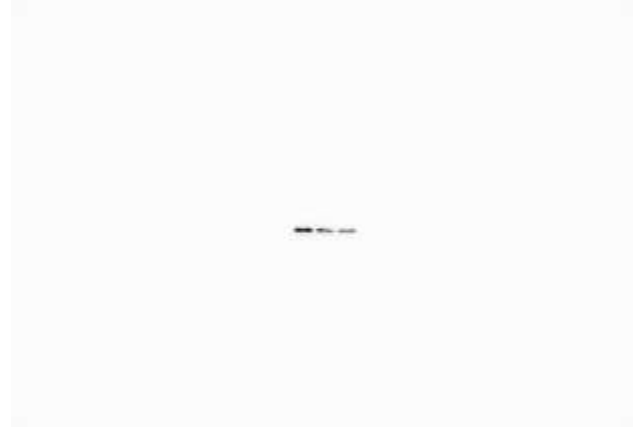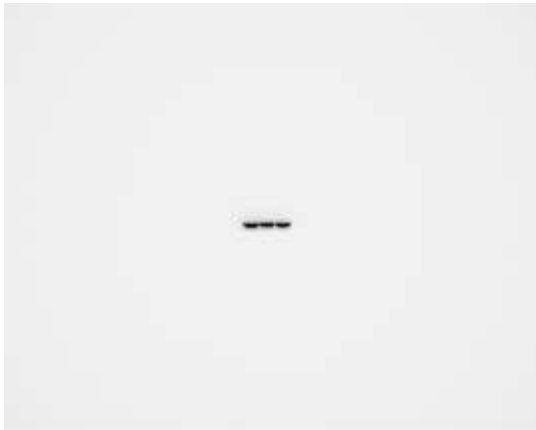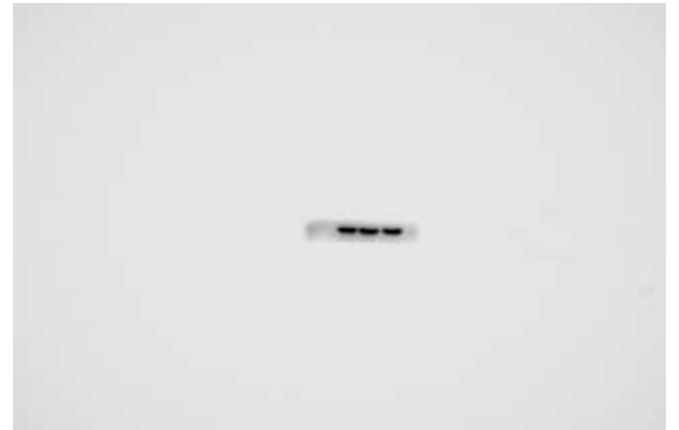

6B

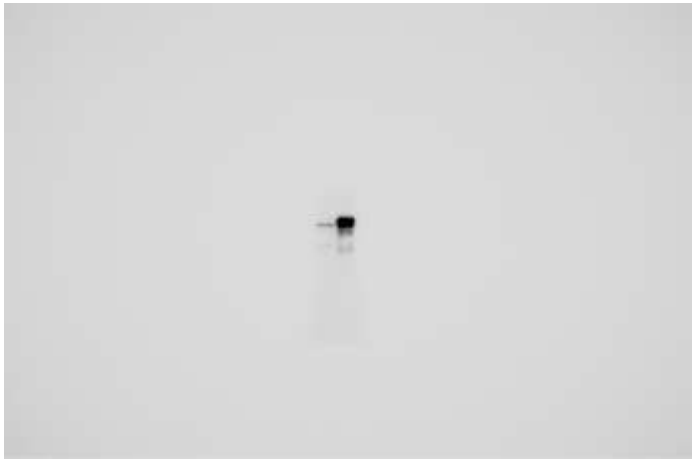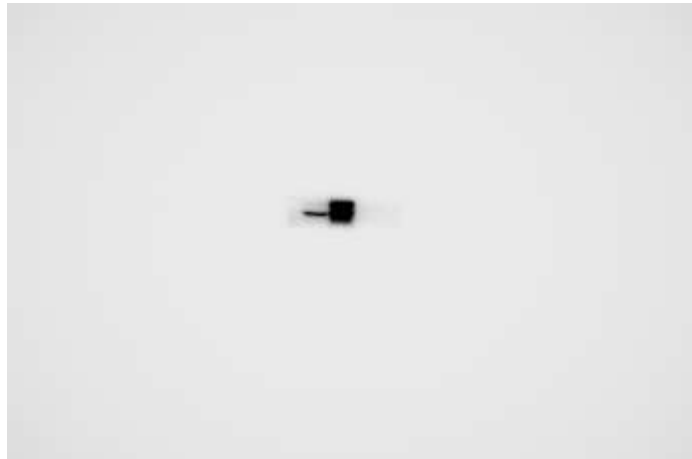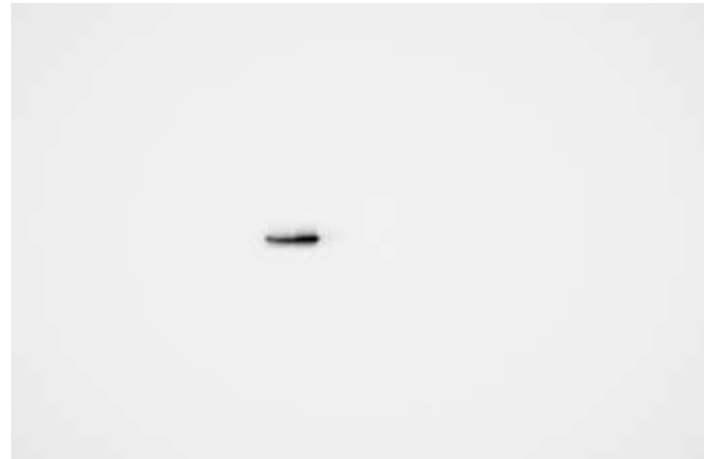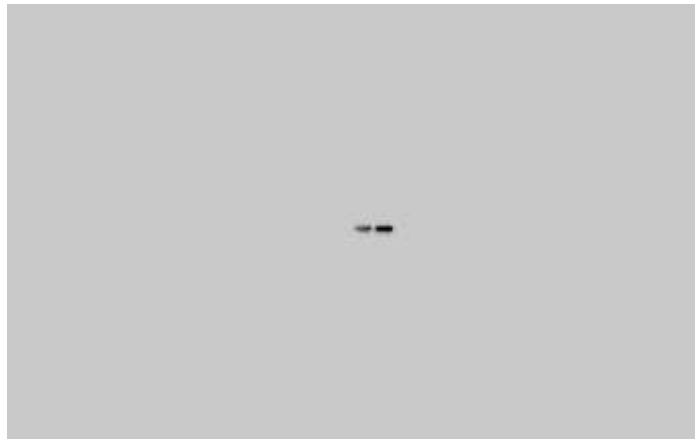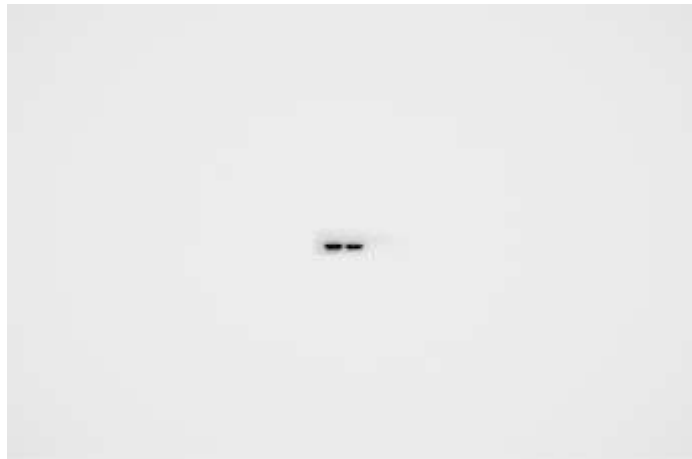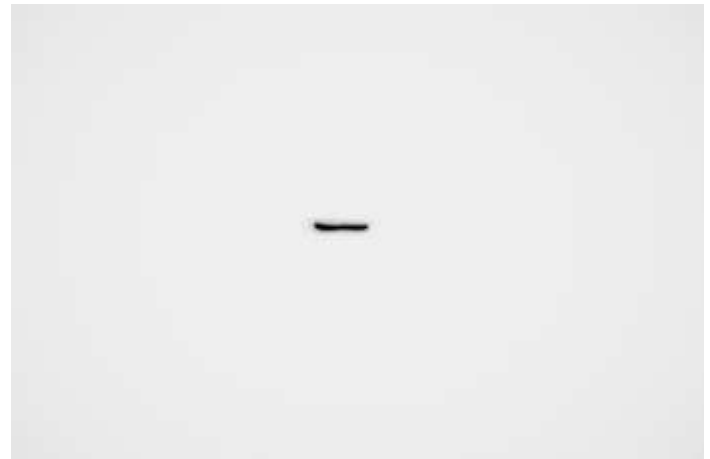

6J

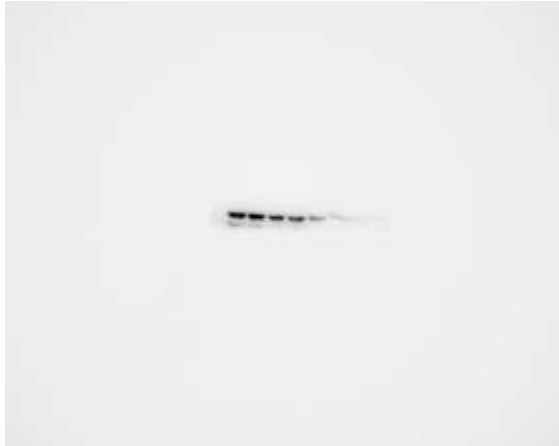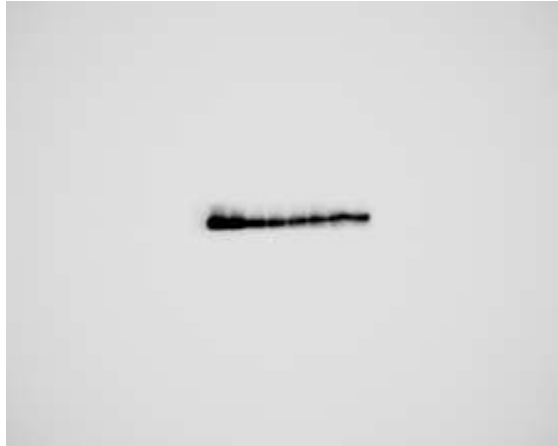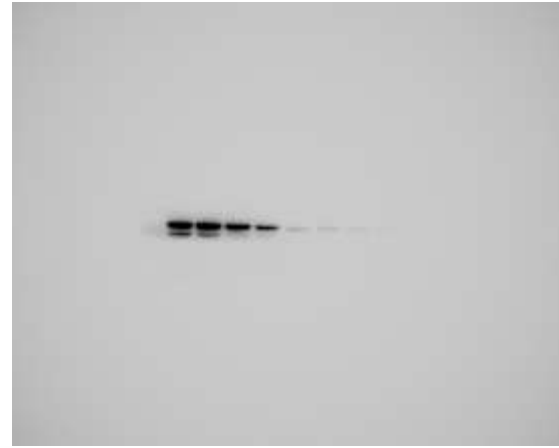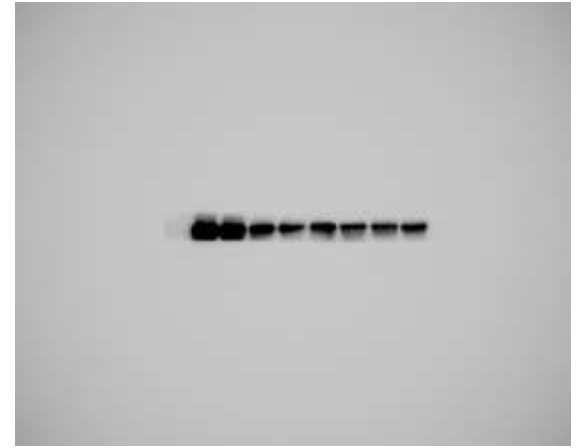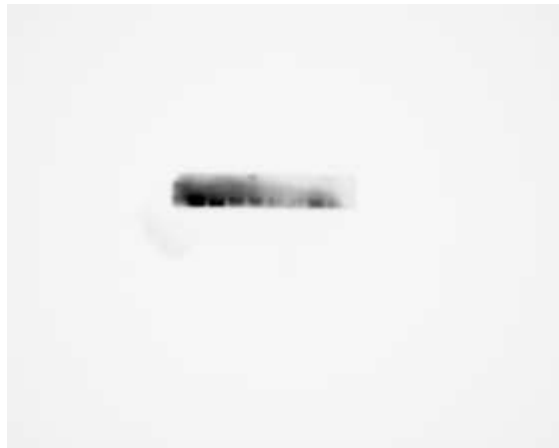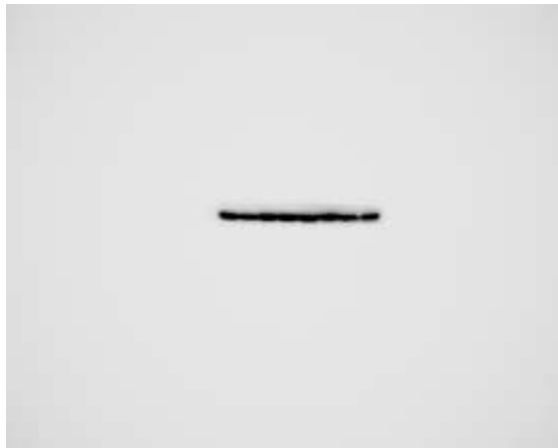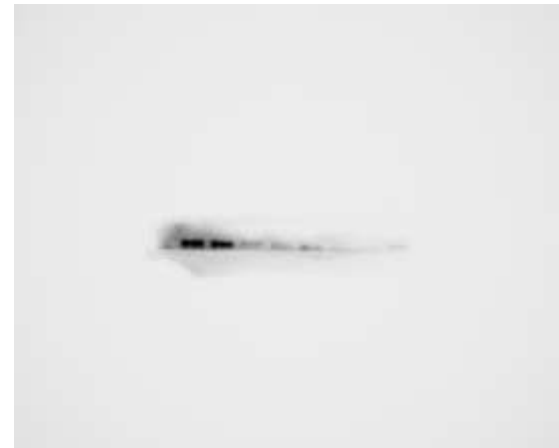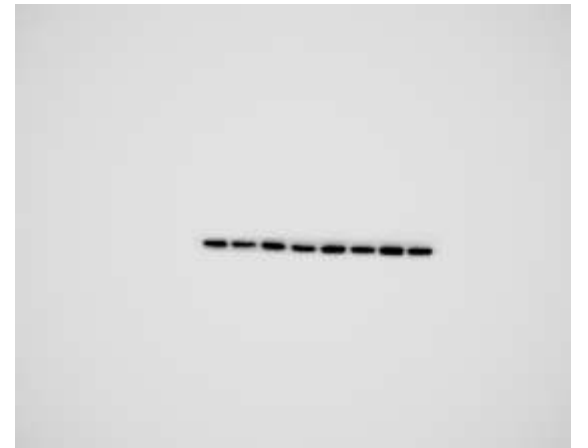

8A

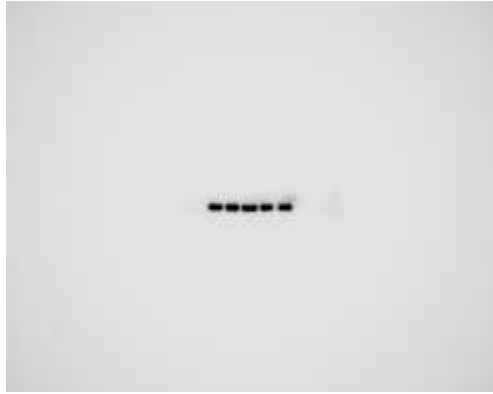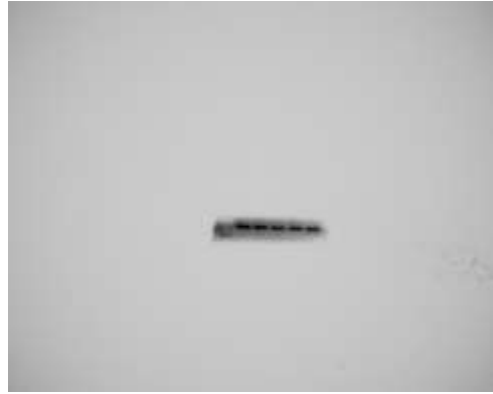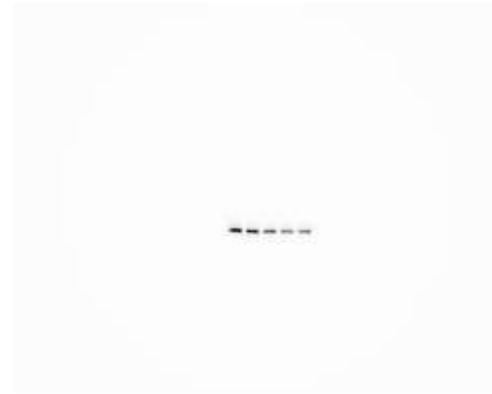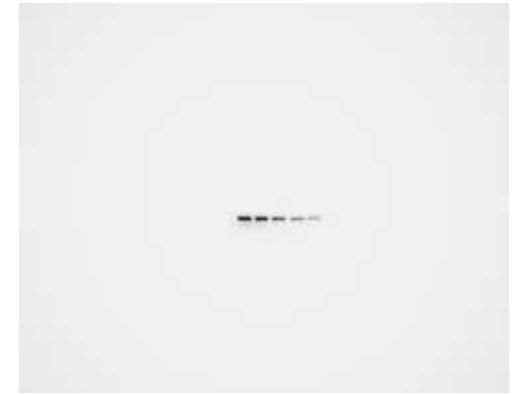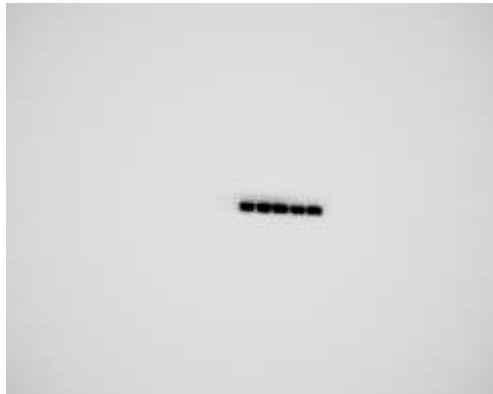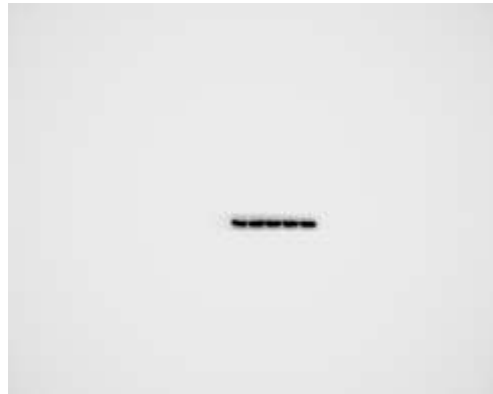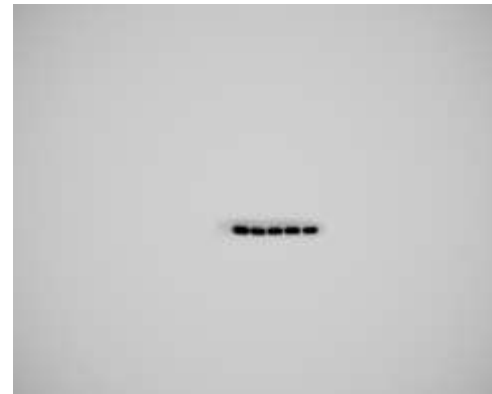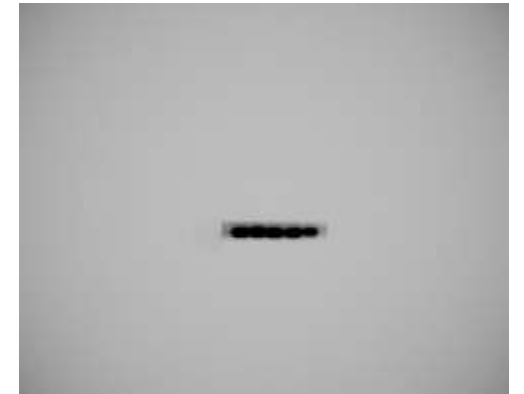

8C

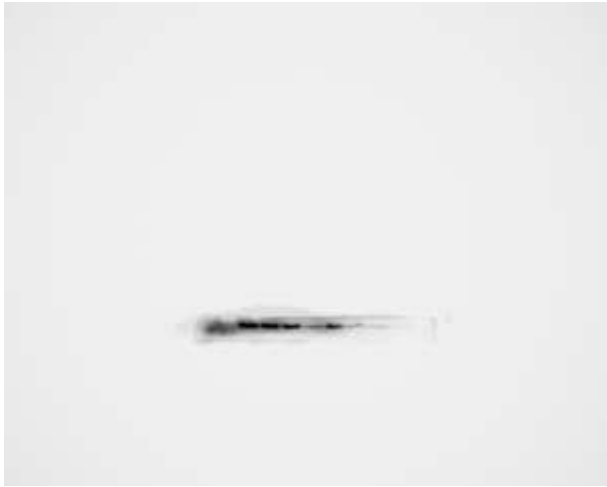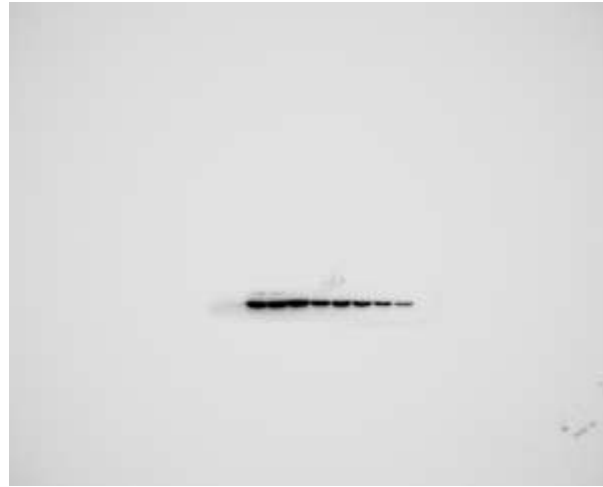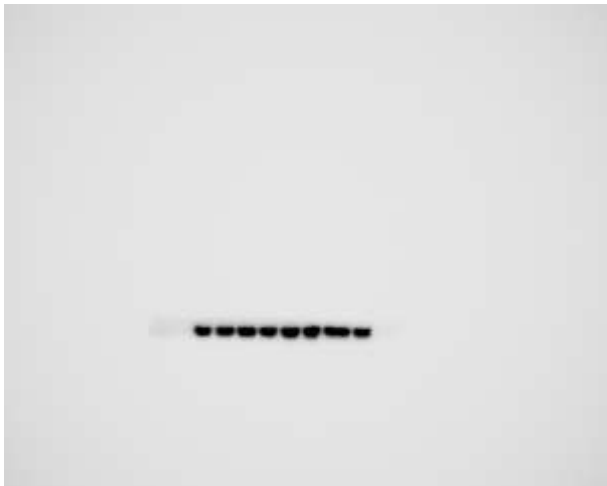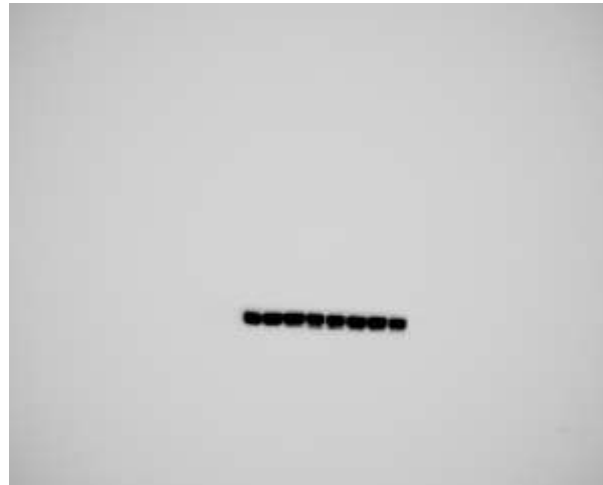

8E

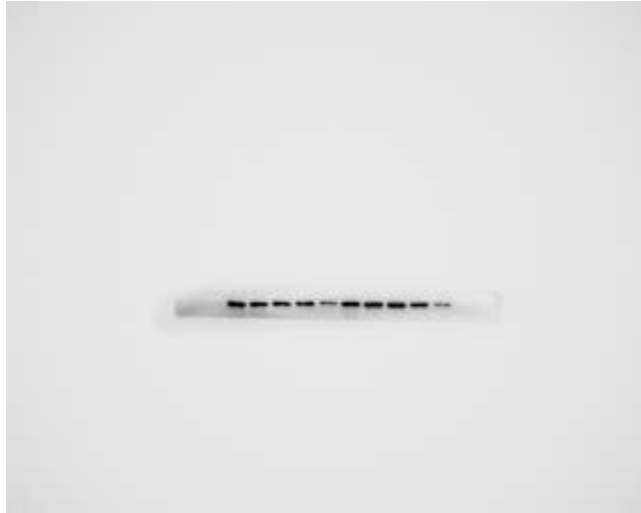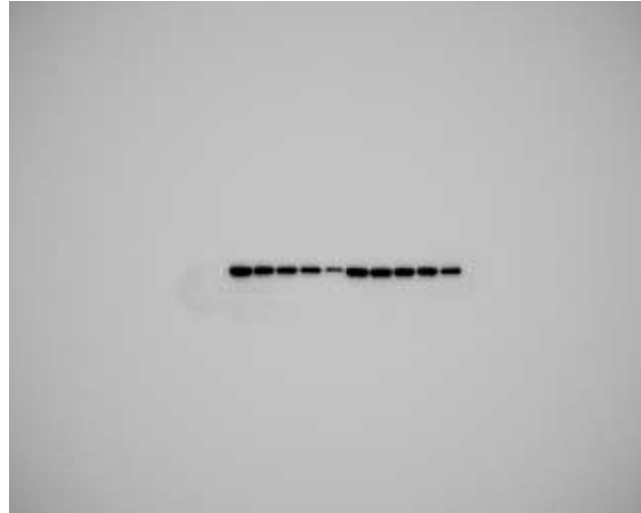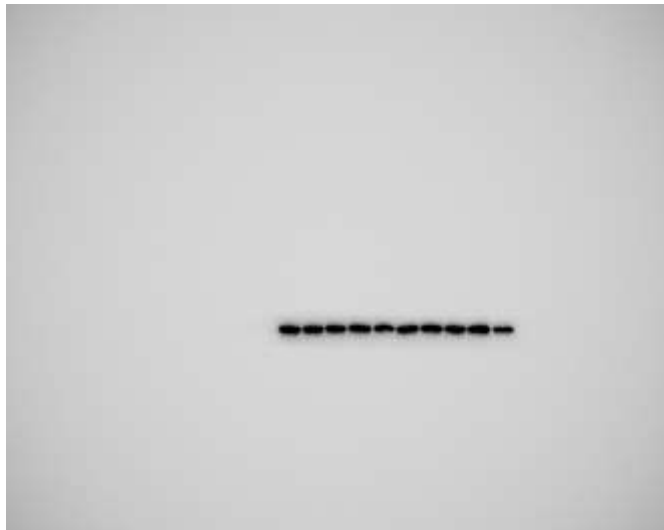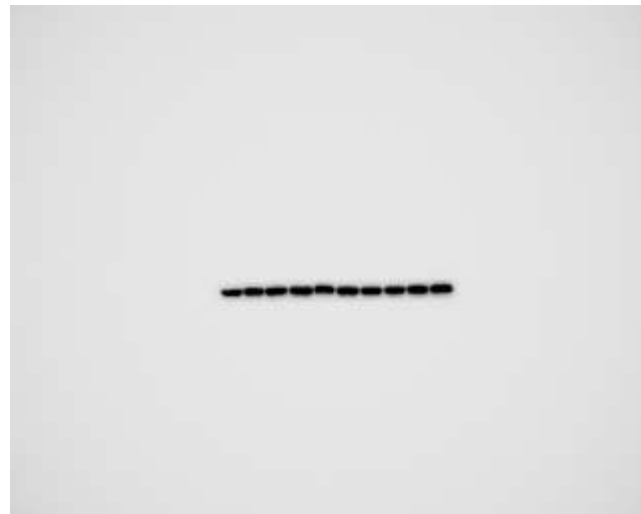

8J

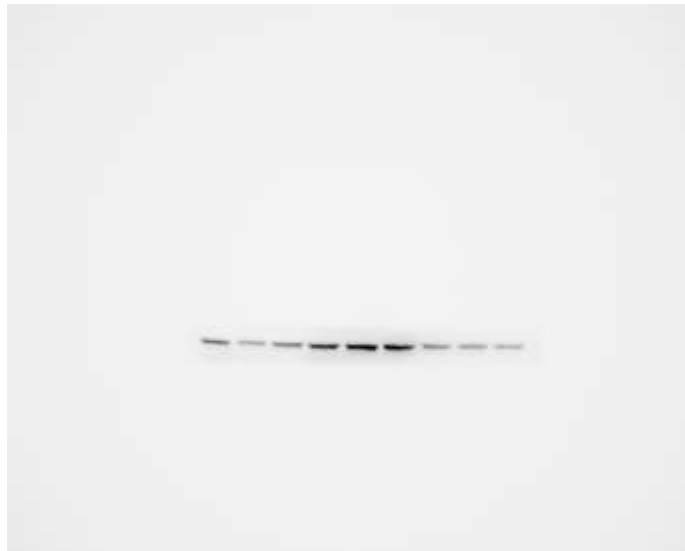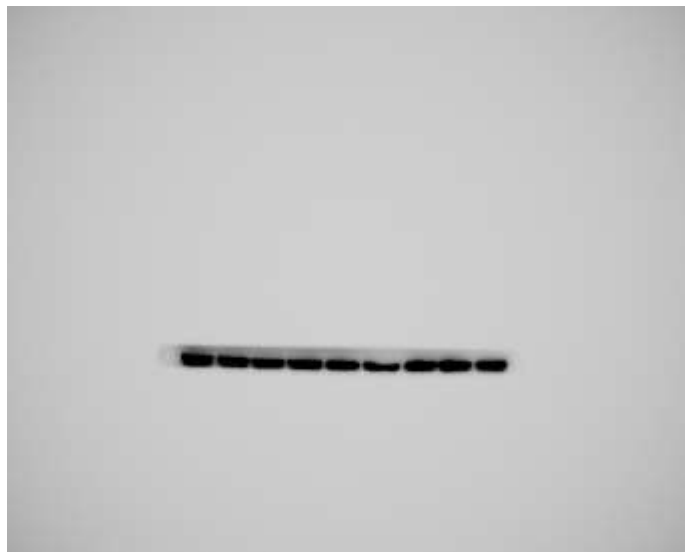

S3G

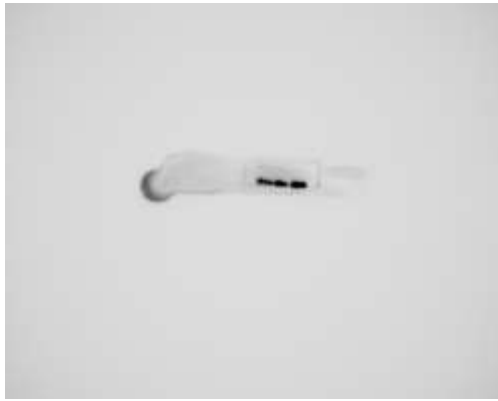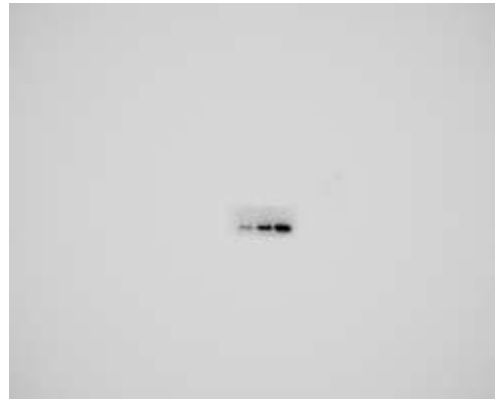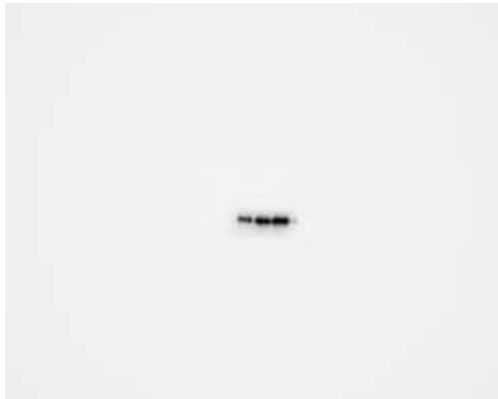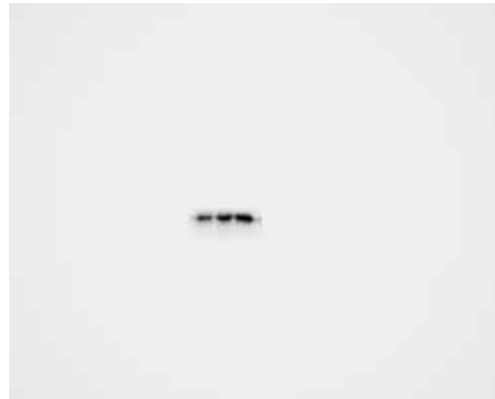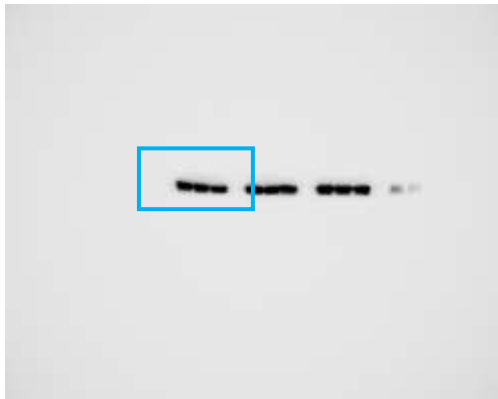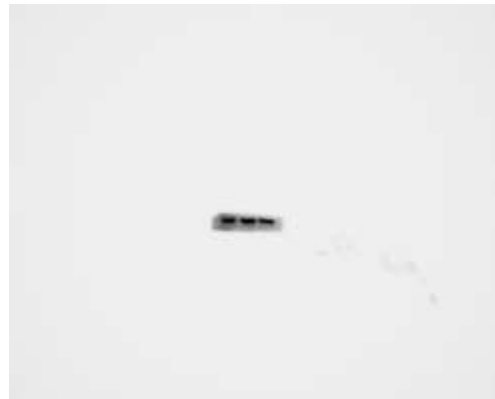

**S6A**

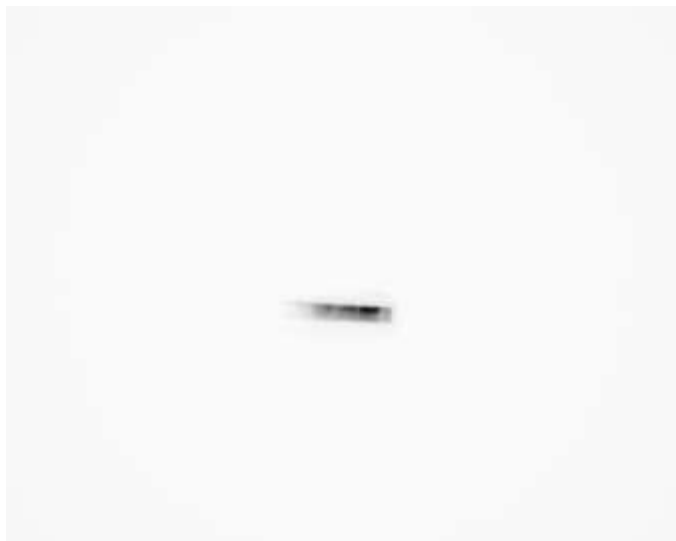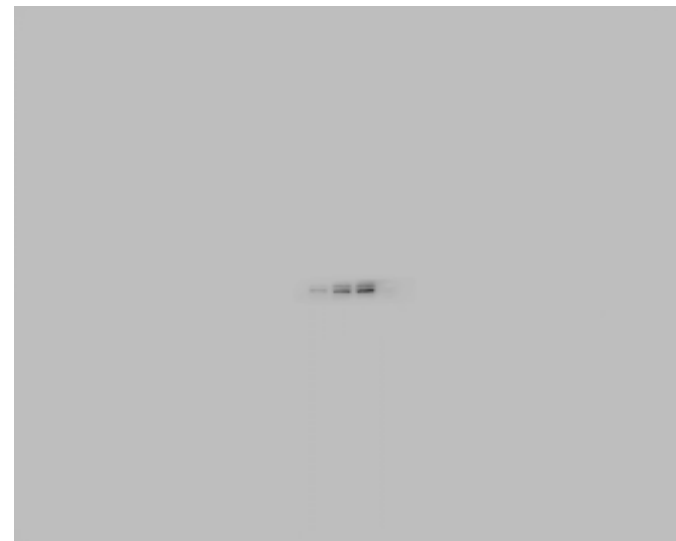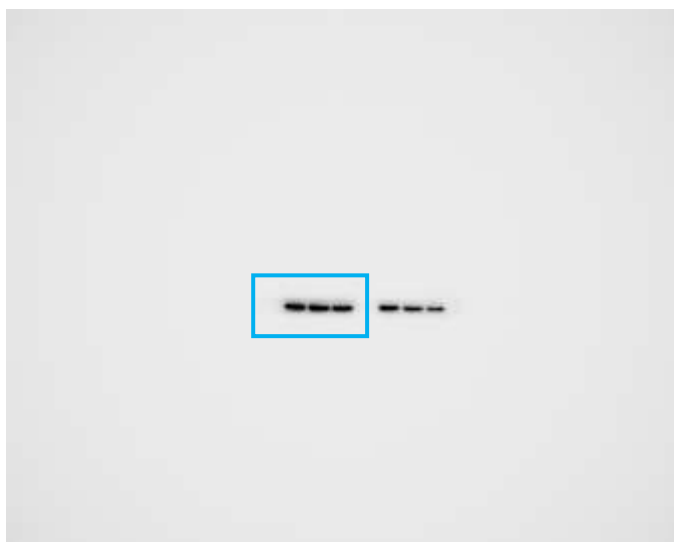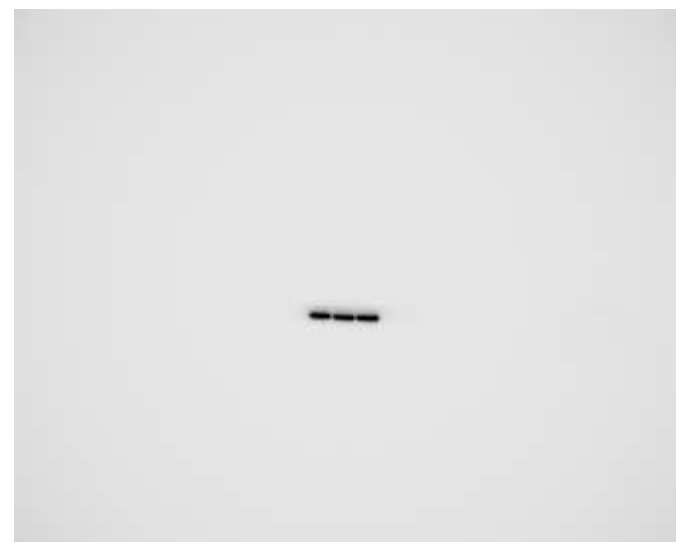

**S6C**

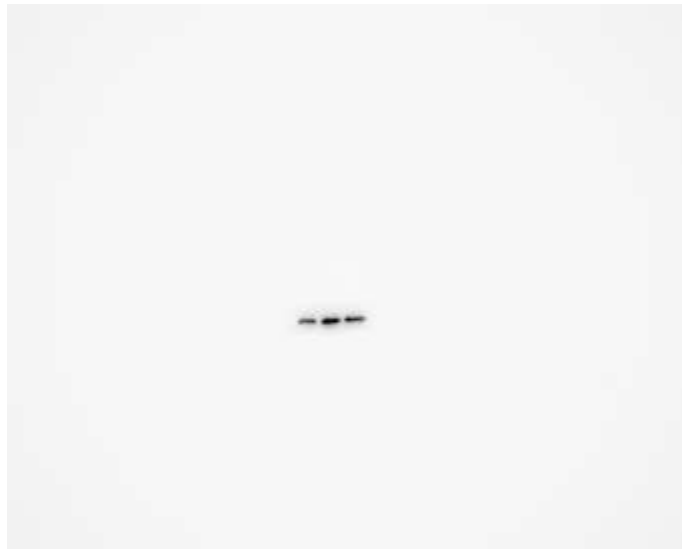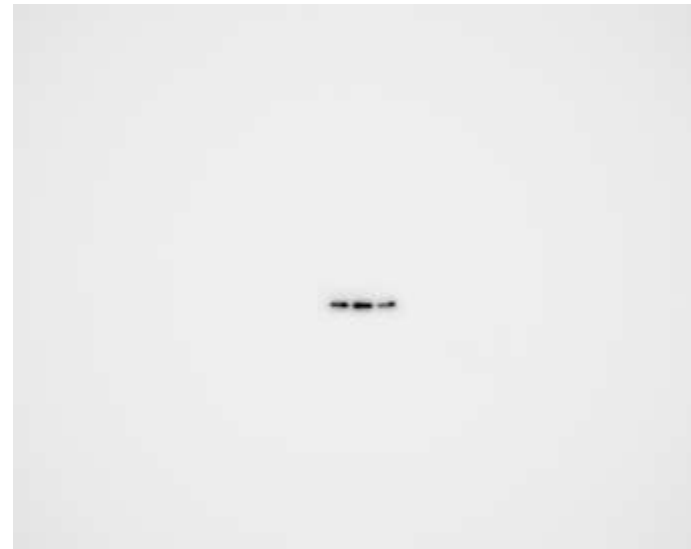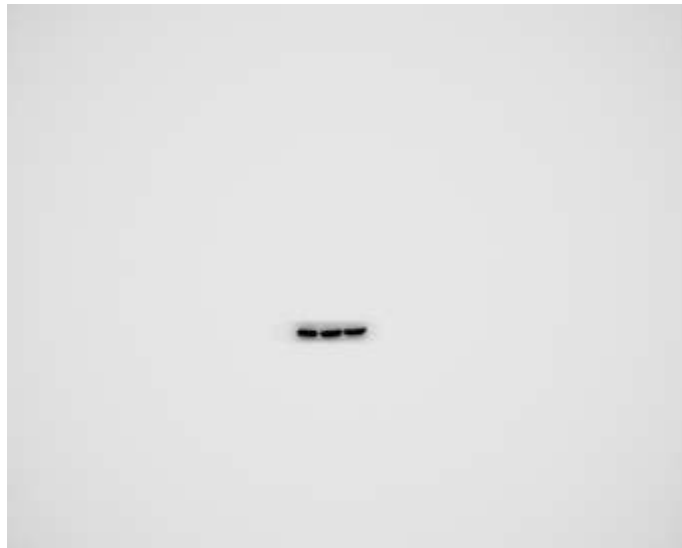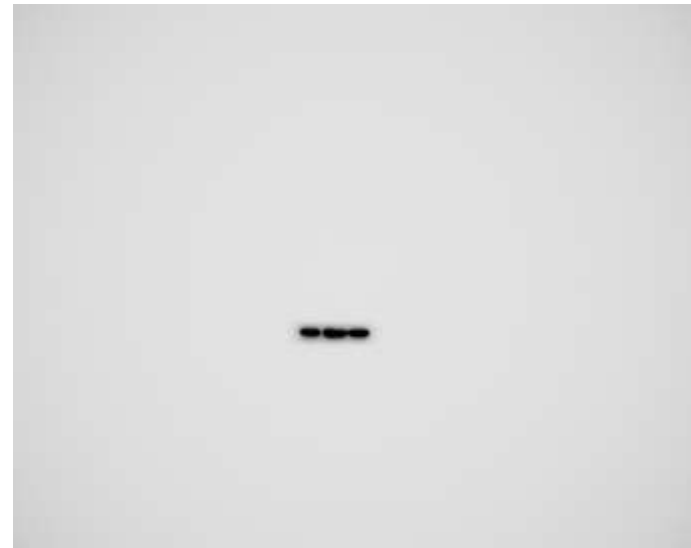

**S6E**

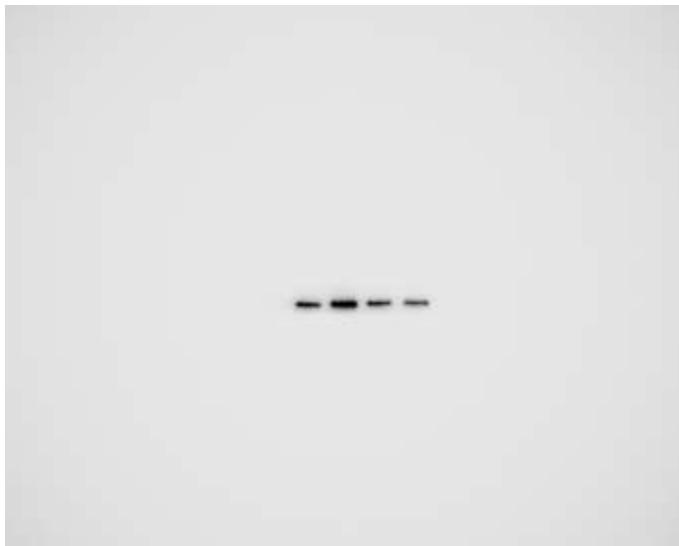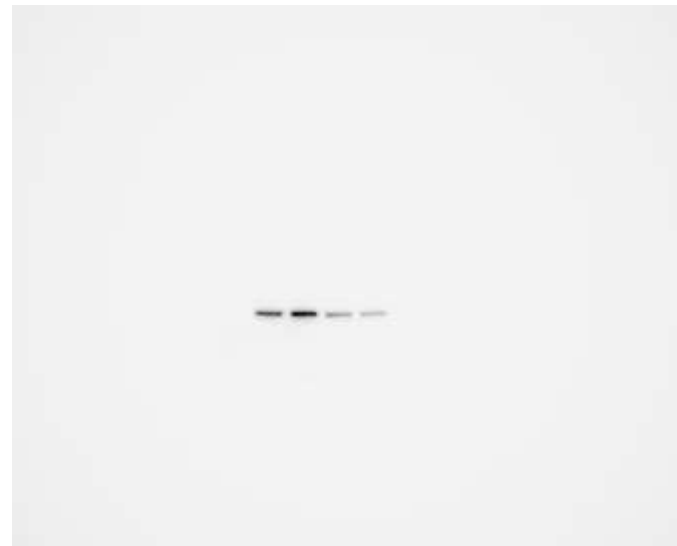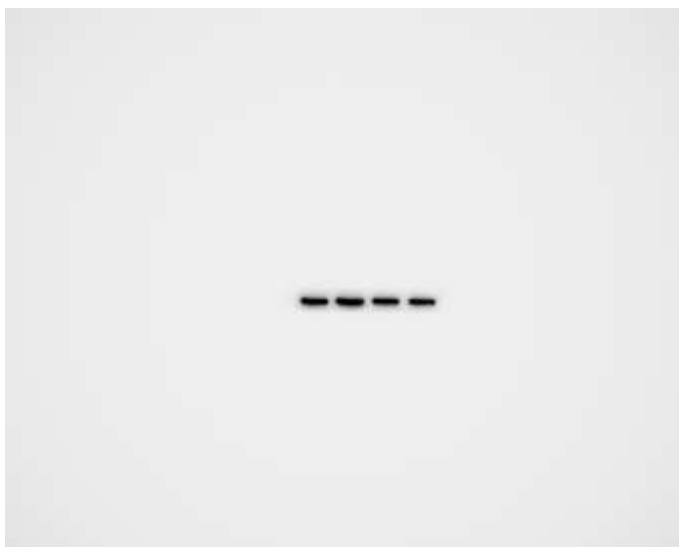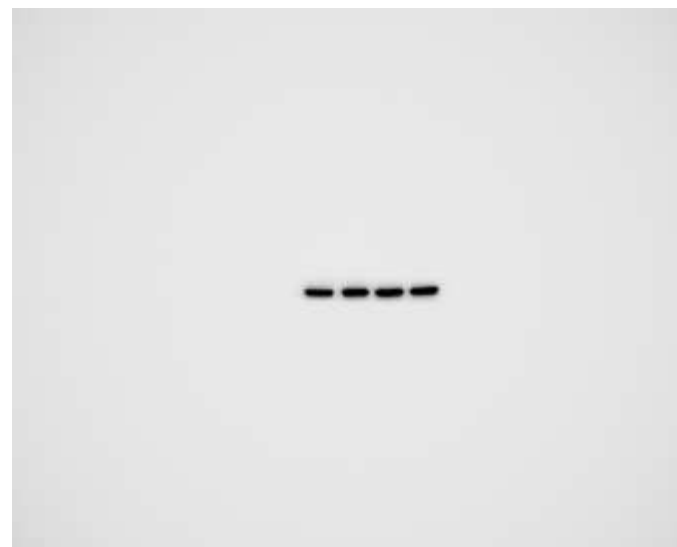

**S6F**

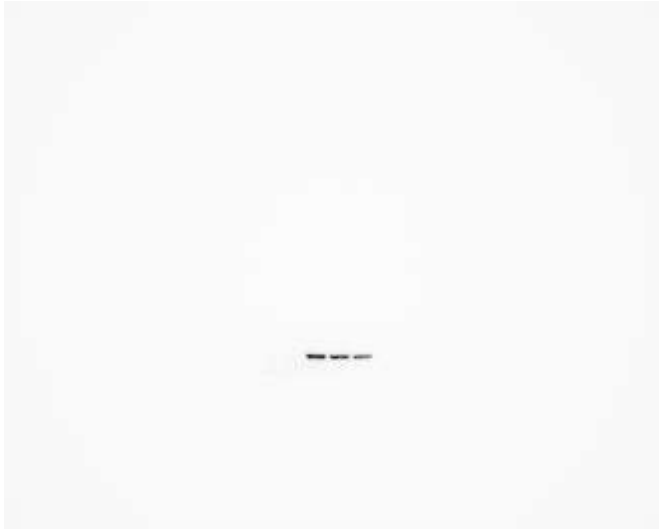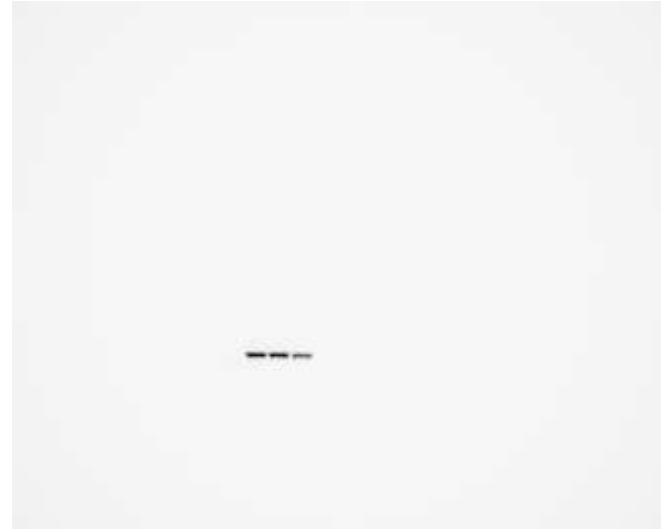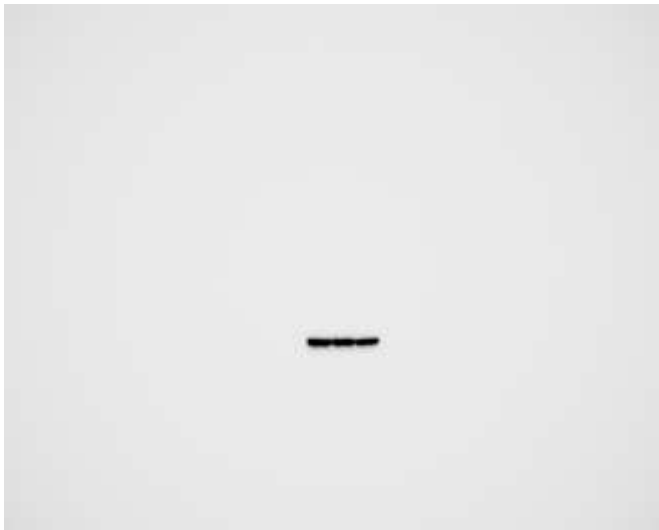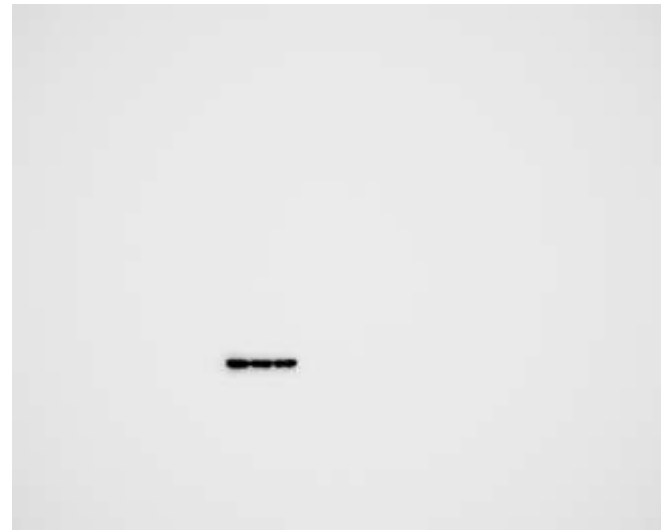

S7C

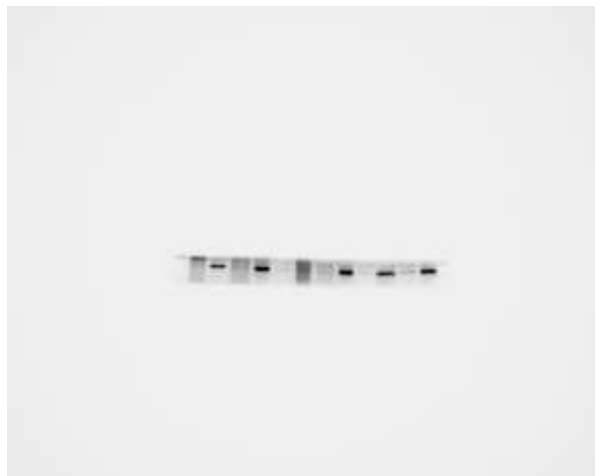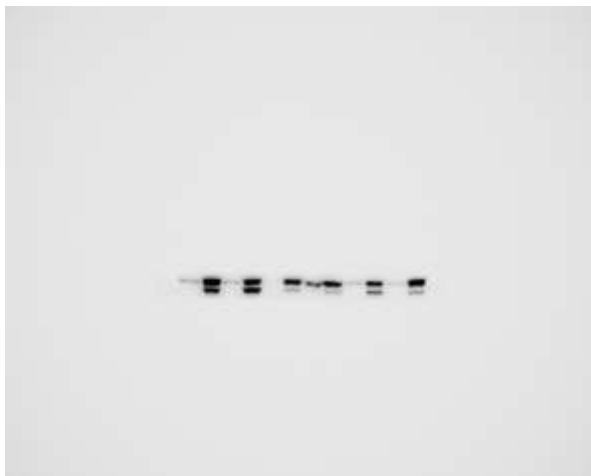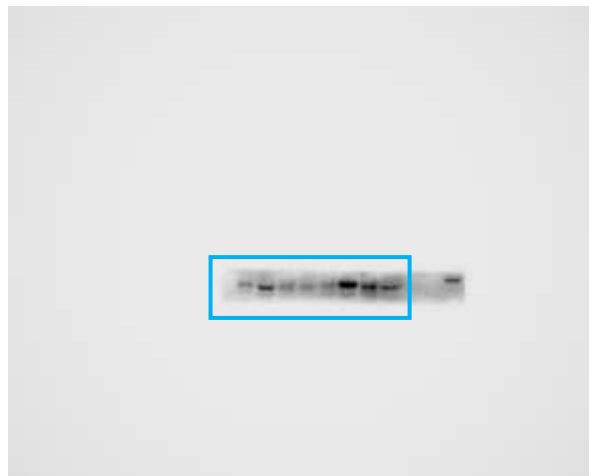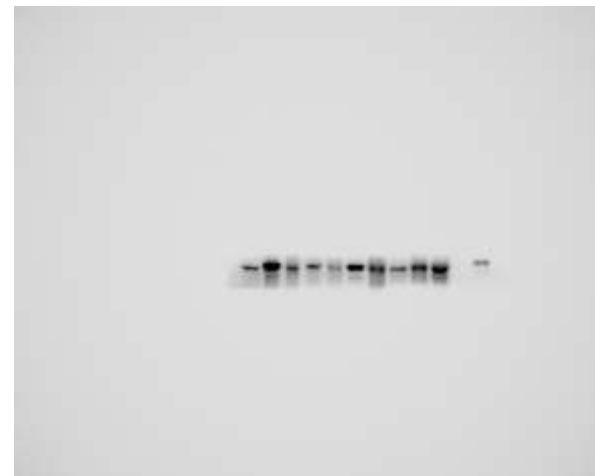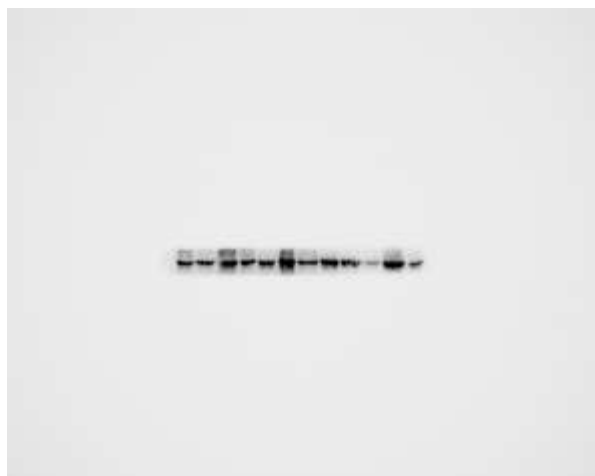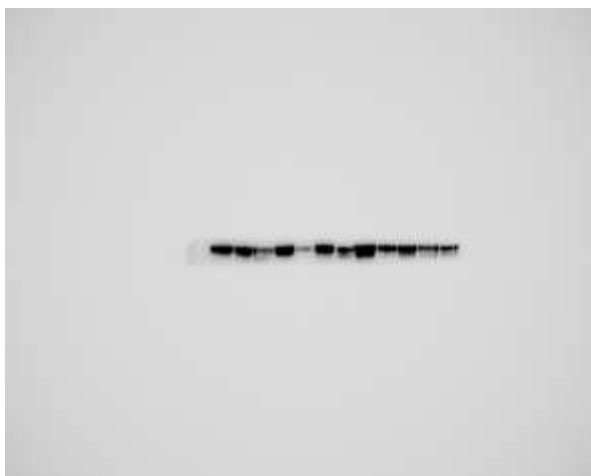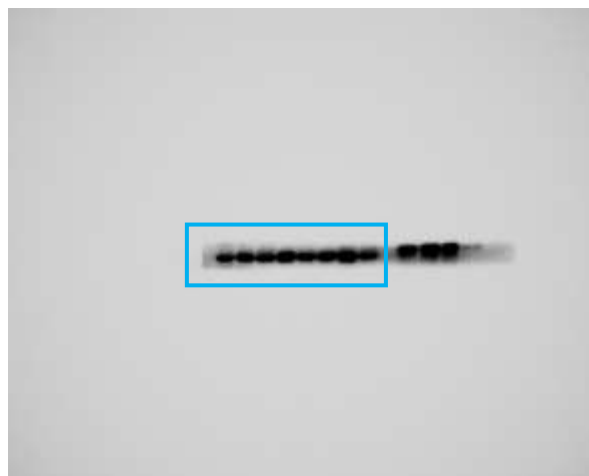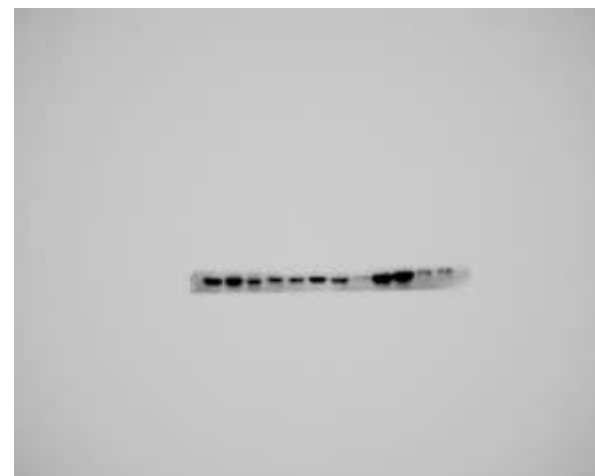

Supplement: Supplementary file 3 — Original WB Data File [file 41418_2024_1285_MOESM3_ESM.pdf]
